# Daphmacropomines A–E: Five Daphniphyllum Alkaloids from *Daphniphyllum macropodum* Miq.

**DOI:** 10.3390/molecules31111943

**Published:** 2026-06-03

**Authors:** Lili Xu, Zhenpeng Niu, Yu Zhang, Hong Liang, Qian Zhao, Sheng Li, Duozhi Chen, Xiao Ding, Xiaojiang Hao

**Affiliations:** 1 State Key Laboratory of Phytochemistry and Natural Medicines, Kunming Institute of Botany, Chinese Academy of Sciences, Kunming 650201, China; 2 University of Chinese Academy of Sciences, Beijing 100049, China; 3 Research Unit of Chemical Biology of Natural Anti-Virus Products, Chinese Academy of Medical Sciences, Beijing 100730, China; 4 Yunnan Characteristic Plant Extraction Laboratory, Kunming 650106, China; 5 Southwest United Graduate School, Kunming 650092, China

**Keywords:** *Daphniphyllum* alkaloids, *Daphniphyllum macropodum* Miq., Structural elucidation, Induce lysosomal biogenesis

## Abstract

Five new *Daphniphyllum* alkaloids, daphmacropomines A–E (**1**–**5**), were isolated from *Daphniphyllum macropodum Miq*. and structurally characterized. Compound **1** is an unprecedented *Daphniphyllum* alkaloid that features a unique 6/9/7/5/5 ring with a rare N-nitroso group. Compound **2** is the second *Daphniphyllum* alkaloid featuring a highly rearranged 6/6/6/7/5/6-fused skeleton. Compounds **3** and **4** are new yuzurimine-type alkaloids, and their structures were determined by extensive techniques including HRESIMS, NMR, ECD, and single-crystal X-ray. A plausible biosynthetic pathway for **1**–**4** is proposed. Furthermore, compounds **3** and **4** can induce lysosomal biogenesis and promote autophagic flux.

## 1. Introduction

**Figure 1:**
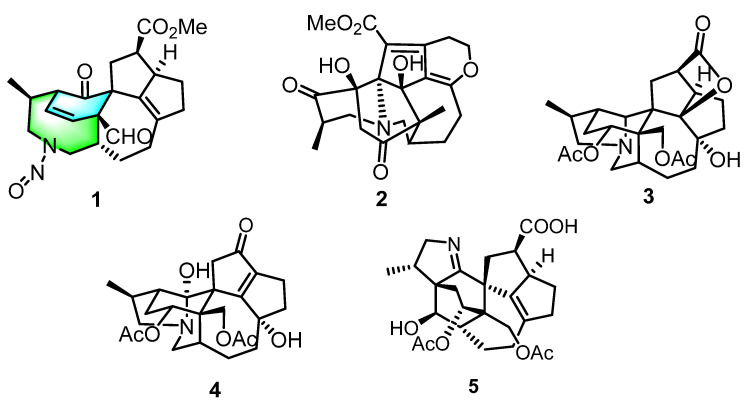
The chemical structures of compounds **1**–**5**.

*Daphniphyllum* alkaloids (>340 members) are natural products isolated from plants of the family *Daphniphyllaceae*. They feature azapolycyclic cage-like frameworks and are known for both their intriguing structures and significant synthetic challenges. Chemists have continuously researched these alkaloids due to their structural complexity and diverse carbon skeletons [1,2,3,4,5,6]. These alkaloids are associated with a wide array of biological activities, including anti-HIV [7], anticancer [8], and vasorelaxant functions [9]. As a traditional folk medicine in China, *Daphniphyllum macropodum* Miq. has been used to treat sores, boils, abscesses, asthma, cough, rheumatism, inflammation, fever due to colds, hepatomegaly and splenomegaly, fractures, and snakebite injuries [10]. Fortunately, our research team discovered in previous studies that daphmacrimine K in *D. macropodum Miq*. has the effect of promoting lysosome biogenesis [11]. In our ongoing search for unique *Daphniphyllum* alkaloids with the ability to promote lysosome biogenesis, we have conducted in-depth research on the stems and leaves of *D. macropodum Miq*. We identified one unusual *Daphniphyllum* alkaloid named daphmacropomine (**1**) from the stems and leaves of *D. macropodum*, together with four previously unreported daphmacropomines B–E (**2**−**5**) (Figure 1). Daphmacropomine A (**1**) is an unprecedented *Daphniphyllum* alkaloid that features a unique 6/9/7/5/5 ring with a rare N-nitroso group. Daphmacropomine B (**2**) is the second *Daphniphyllum* alkaloid featuring a highly rearranged 6/6/6/7/5/6-fused skeleton. Additionally, we found that daphmacropomines C (**3**) and D (**4**) can induce lysosomal biogenesis and promote autophagic flux.

## 2. Results and Discussion

The five new alkaloids were obtained from the crude alkaloid extract by a combination of sequential column chromatography and semipreparative HPLC.

Daphmacropomine A (**1**) was obtained as a colorless crystal. Its molecular formula was determined as C_23_H_28_N_2_O_5_ based on NMR data (Table 1) and a positive HRMS (ESI) peak at *m*/*z* 435.1891 [M + Na]^+^ (calcd for C_23_H_28_N_2_O_5_Na, 435.1890), consistent with 11 degrees of unsaturation. The IR absorptions of **1** implied the presence of nitroso-group (1384 cm^−1^) functionality. All 23 carbon signals observed in the ^13^C NMR and DEPT spectra of **1** could be classified into two methyl groups [*δ*_C_ 51.8 and 16.9], seven methylenes [*δ*_C_ 59.1, 53.9, 42.1, 39.8, 31.0, 28.0, and 26.9], eight methines [*δ*_C_ 205.5, 131.8, 128.2, 53.7, 49.5, 47.3, 41.5, and 31.9], and six quaternary carbons [*δ*_C_ 206.7, 175.0, 140.5, 139.8, 60.2, and 58.7]. Among them, two methylenes (*δ*_C_ 53.7 and 59.1) were assigned to nitrogen-bound methylene groups.

**Figure 2:**
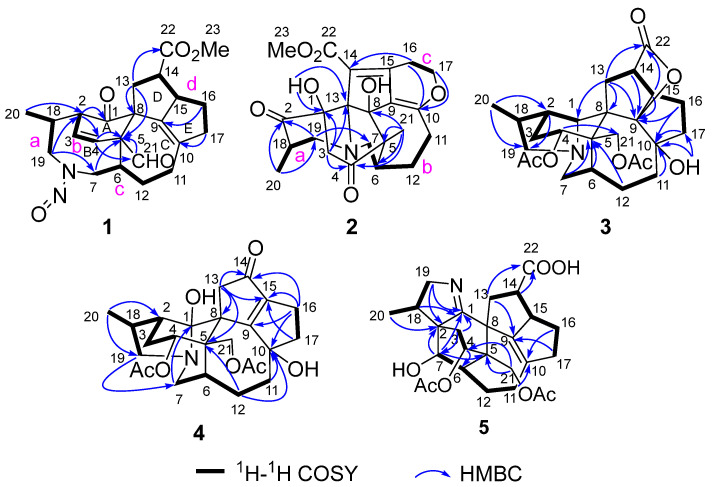
^1^H−^1^H COSY and key HMBC correlations of **1**–**5**.

**Figure 3:**
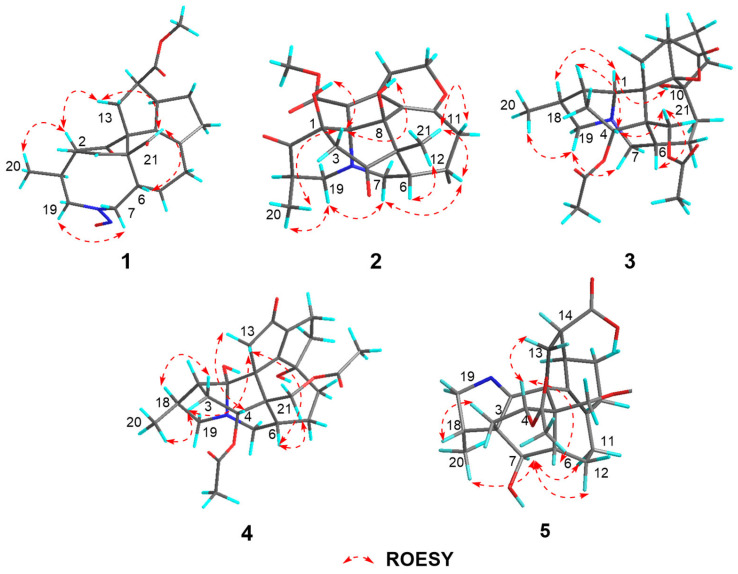
ROESY correlations of **1**–**5**.

The following structural fragments were established through ^1^H−^1^H COSY data, which enabled the establishment of four spin coupling systems (a−d), as shown in Figure 2. Two moieties were identified in compound **1** through detailed 2D NMR analysis. The right moiety (rings C, D and E) contained three fused ring systems (two 5-membered rings and one 7-membered ring) with an aldehyde at C-5 (*δ*_C_ 60.2), a double bond between C-9 (*δ*_C_ 140.5) and C-10 (*δ*_C_ 139.8), a carbomethoxy group at C-14 (*δ*_C_ 41.5), and a methylene at C-6 (*δ*_C_ 47.3), which was identical to that of daphhimalenine B [12,13]. In the left moiety (rings A and B), the HMBC correlations of H-19b (*δ*_H_ 3.86, dd, *J* = 13.0, 4.5 Hz) to C-7 (*δ*_C_ 59.1) confirmed that the nitrogen atom linked C-7 and C-19. The HMBC cross-peaks of H-2 (*δ*_H_ 3.26, t, *J* = 3.9, 1.9 Hz) to C-1 (*δ*_C_ 206.7) and C-4 (*δ*_C_ 131.8) and of H_3_-20 (*δ*_H_ 1.08, d, *J* = 7.2 Hz) to C-2 (*δ*_C_ 49.5) established the connectivity of spin systems **a** and **b**. Moreover, the HMBC correlations of H-13a/C-8 and H-4/C-5 suggested C-1-C-8 and C-4-C-5 bond linkages between both moieties. Thus, the planar structure of daphhimalenine A, which was established as shown in Figure 1, has a unique 6/9/7/5/5 ring system that contains *N*-nitroso. Moreover, daphhimalenine A is the first isolated *Daphniphyllum* alkaloid with an *N*-nitroso group to date. The relative configuration of compound **1** was determined via ROESY analysis. Cross-peaks between H-21/H-12b, H-20/H-19b, H-19b/H-7b, and H-7a/H-11a indicated that H_3_-20, H-19b, H-7b, and H-21b are cofacial (Figure 3).

**Figure 4:**
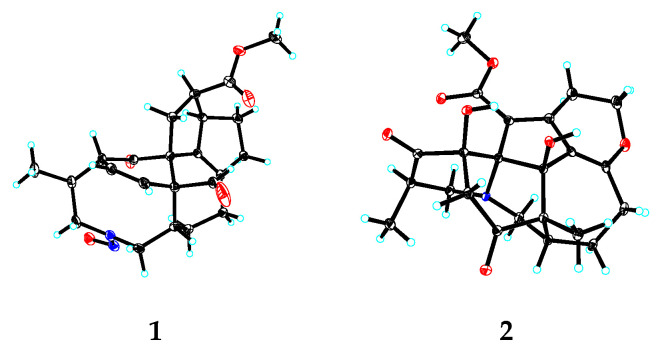
ORTEP drawing of **1** and **2**. Thermal ellipsoids are shown at 50% probability. Color code: blue, N; gray, C; red, O.

Fortunately, high-quality crystals of **1** [Figure 4; Flack parameter = 0.062(12)] were obtained, and the structure was unambiguously confirmed through a subsequent single-crystal X-ray study (CCDC 2296227), which revealed that the absolute configuration of **1** was 2*R*, 5*R*, 6*S*, 8*R*, 14*R*, 15*R*, 18*S* [14,15]. Importantly, daphmacropomine A (**1**) represents the first *Daphniphyllum* alkaloid reported to contain an N-nitroso moiety.

Daphmacropomine B (**2**) was obtained as colorless crystals, and its molecular formula was determined to be C_23_H_27_NO_7_ with eleven IHDs based on its HRESIMS and ^13^C NMR data (Table 1). The NMR data of **2** (Table 1) were very similar to those of dcalycinumines A [13], except for the CH_3_-20 peaks (*δ*_H_ 1.35, *δ*_C_ 19.7). However, the detailed 2D NMR data demonstrated that **2** is a diastereoisomer of dcalycinumine A.

Key ROESY cross-peaks (H_3_-21/H-6/H-12b, H-12a/H-7a, H-7a/H-19a, and H-19a/H_3_-20) established the relative stereochemistry of compound **2** (Figure 3). Its absolute configuration was unambiguously determined as 1*R*, 5*S*, 6*S*, 8*S*, 13*S*, 18*S* by single-crystal X-ray diffraction (XRD) analysis (Figure 4), with a Flack parameter of 0.09(4). The crystallographic data have been deposited at the Cambridge Crystallographic Data Centre (CCDC 2296251) [14,15].

**Figure 5:**
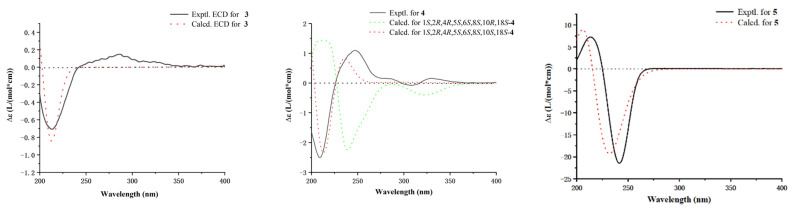
Comparison of the experimental and calculated ECD spectra of compounds **3**, **4**, and **5**.

Compound **3** (daphmacropomine C) was assigned the molecular formula C_26_H_35_NO_7_ based on NMR data (Table 1) and an HRESIMS ion at *m*/*z* 474.2490 [M + H]^+^ (calcd 474.2486), indicating 10 degrees of unsaturation. Its NMR spectrum (Table 1) was similar to that of yuzurimine [16]. The absence of a C-9/C-2 ether linkage was confirmed by 2D NMR. The HMBC correlations from H-13 (*δ*_H_ 3.04, m), H-14 (*δ*_H_ 2.78, d, *J* = 3.0 Hz), and H-15 (*δ*_H_ 3.06, m) to C-9 (*δ*_C_ 111.0) revealed a linkage between C-9 and C-22, forming a six-membered lactone. In addition, a tertiary hydroxyl group appears at C-10. The HMBC correlations (Figure 2) from H-16a (*δ*_H_ 1.82, m), H-17 (*δ*_H_ 2.27, m) and OH-10 (*δ*_H_ 8.91, s) to C-10 (*δ*_C_ 76.7) confirmed this assignment. Therefore, the planar structure of **3** was established. The relative configuration of **3** was assigned by inspection of its ROESY data (Figure 3). The OH-10 was restricted to an *α*-orientation based on the ROESY cross-peaks of H-1/OH-10. Finally, the absolute configuration (1*S*, 2*R*, 4*R*, 5*S*, 6*S*, 8*R*, 9*R*, 10*S*, 14*R*, 15*R*, 18*S*) was assigned by compariso n of experimental and calculated ECD curves (Figure 5). 

Daphmacropomine D (**4**) was assigned the molecular formula C_25_H_33_NO_7_ on the basis of its HR-ESI-MS at *m*/*z* 460.2333 [M + H]^+^ (calcd for C_25_H_33_NO_7_, 460.2330). Comparison of the ^13^C NMR data (Table 2) of 4 with those of calycinumine A [17] indicated a close resemblance. The placements of acetoxyl groups at C-21 and C-4 in **4** were determined from its COSY and HMBC data, respectively. The ROESY experiment (Figure 3) suggested that the relative configuration of **4** is largely consistent with that of yuzurime-type *Daphniphyllum* alkaloids [16], except that the OH-10 was not observed in its ROESY data. The absolute configuration was assigned as 1*S*, 2*R*, 4*R*, 5*S*, 6*S*, 8*S*, 10*S*, 18*S* based on the results of experimental and calculated ECD results (Figure 5) of (1*S*, 2*R*, 4*R*, 5*S*, 6*S*, 8*S*, 10*S*, 18*S*)-**4** and (1*S*, 2*R*, 4*R*, 5*S*, 6*S*, 8*S*, 10*R*, 18*S*)-**4**.

Daphmacropomine E (**5**) was obtained as a white amorphous solid. Its molecular formula was determined as C_26_H_33_NO_7_ by HR-ESI-MS. The 1D NMR spectral data of **5** (Table 2) closely resembled those of daphtenidine C [18], except that the methoxy group at C-22 was replaced by a carboxyl group. HMBC correlations from H-14/H-13 to C-22 (*δ*_C_ 177.7) (Figure 2) confirmed that this carboxyl group was attached to C-1. Additionally, the ROESY correlations (Figure 3) supported the relative stereochemistry of the ring system, which was consistent with that of daphtenidine C [18]. Thus, the structure of **5** was elucidated as a C-22 carboxylic acid analog of daphtenidine C, and it was named daphmacropomine E.

**Scheme 1:**
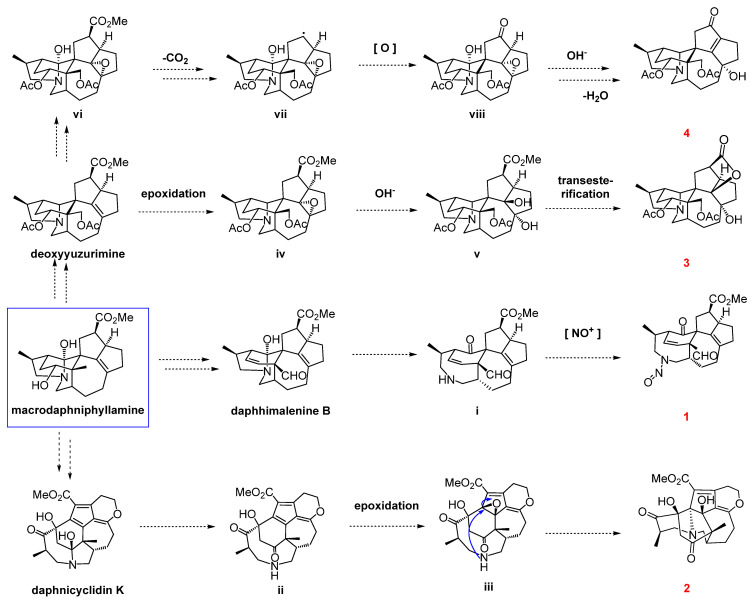
Proposed biosynthetic pathway for compounds **1**–**4**.

The speculative biogenetic pathways for **1**−**4** could be traced back to a yuzurimine-type alkaloid, macrodaphniphyllamine [19]. Compound **1** might then arise from a derivative such as daphhimalenine B [12] via an intermediate (i) involving cleavage of the N-1–C-1 bond, followed by nitrosylation. Compound **2** could be formed from daphnicyclidin K [20] through a key epoxidation step (iii) and subsequent ring cleavage. Further oxidation and reduction steps may lead to deoxyyuzurimine [21], which could then be converted to **3** via epoxidation and intramolecular transesterification. Finally, **4** might be derived from macrodaphniphyllamine through decarboxylation, oxidation, and elimination. It should be noted that these pathways remain hypothetical and are presented primarily to account for the structural relationships among the isolated compounds (Scheme 1).

Following structural elucidation of compounds **1**–**5**, we turned our attention to their biological evaluation. Based on our prior finding that daphmacrimine K induces lysosomal biogenesis, compounds **1**–**4** were tested for their ability to promote lysosomal biogenesis and autophagic flux. The concentration (40 μM) and treatment durations for compounds **3** and **4** were based on our previous study of daphmacrimine K [11], which induces lysosomal biogenesis at 40 μM without significant cytotoxicity.

**Figure 6:**
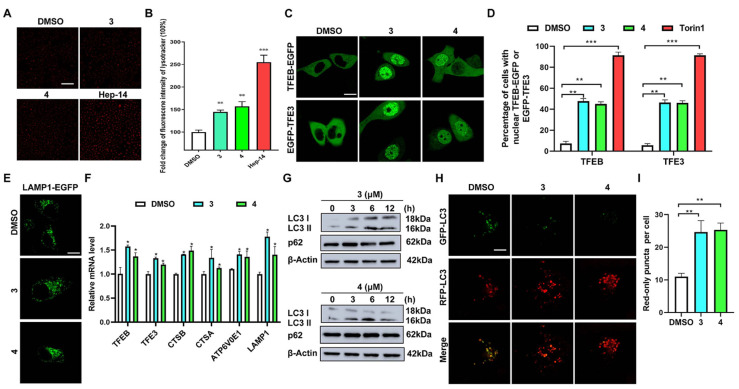
Identification of compounds **3** and **4**, which induce lysosomal biogenesis and promote autophagy. (**A**,**B**) Compounds **3** and **4** induced lysosomal biogenesis in HepG2 cells. Compounds **3** and **4** were tested in HepG2 cells by LysoTracker Red staining. DMSO served as the negative control and Hep-14 (40 μM) as the positive control. Scale bar = 100 μm. (**C**,**D**) Compounds **3** and **4** (40 μM) promoted the nuclear translocation of TFEB and TFE3 in HepG2 cells; scale bar = 10 μm. (**E**) LAMP1-EGFP expression in HepG2 cells following 12 h exposure to compounds **3** or **4** (40 μM), scale bar = 8 μm. (**F**) Compounds **3** and **4** induced the expression of lysosomal genes. The data are presented as the mean ± SD. (**G**) HepG2 cells were treated with the indicated concentrations of compounds 3 and 4 for 0, 3, 6, and 12 h, and expression levels of the proteins were detected by Western blot analysis. (**H**,**I**) RFP-GFP-LC3 puncta imaging (**H**) and quantification (**I**) in HepG2 cells treated with compounds **3** and **4** (40 μM) or DMSO for 12 h; scale bar = 10 μm. * *p* < 0.05, ** *p* < 0.01, *** *p* < 0.001.

To examine whether compounds **1**–**4** promote lysosomal biogenesis, LysoTracker Red staining was used to assess lysosomal induction. The results indicated that compounds **3** and **4** enhanced the intensity of LysoTracker staining by 148.5% and 163.5%, respectively (Figure 6A,B). After treatment with compounds **3** (40 μM) and **4** (40 μM) for 4 h, significant nuclear accumulation of TFEB and TFE3 was observed in HepG2 cells (Figure 6C,D). Real-time quantitative PCR analysis revealed that compounds **3** and **4** upregulated the mRNA levels of several autophagy-lysosome-related genes, including lysosomal tissue hydrolases (CTSA, CTSB) and lysosomal-associated membrane protein 1 (LAMP1) (Figure 6F). These findings suggest that compounds **3** and **4** transcriptionally regulate the expression of multiple autophagy- and lysosome-related genes, thereby enhancing autophagy and lysosomal biogenesis. Notably, compounds **3** and **4** significantly increased LAMP1 expression, which colocalized with LysoTracker Red (Figure 6E). Treatment with either compound **3** or **4** led to enhanced formation of the autophagy marker LC3-II in HepG2 cells (Figure 6G). Furthermore, treatment with either compound **3** or **4** resulted in an increase in RFP-LC3 puncta and a decrease in GFP signal, indicative of autophagy activation (Figure 6H,I).

## 3. Experimental

### 3.1. Plant Materials

The stems and leaves of *D. macropodum Miq*. were collected from Emei County, Sichuan Province, China, in August 2019. The sample was identified by Qi Yu of Kunming Institute of Botany. Voucher specimens (CIBYQ58B016) were deposited at the Germplasm Bank of Wild Species, Kunming Institute of Botany, Chinese Academy of Sciences.

### 3.2. General Experimental Procedures

Optical rotations were determined using an Autopol VI digital polarimeter (Rudolph Research Analytical, Hackettstown, NJ, USA). UV spectra were recorded on a Shimadzu UV2700 spectrophotometer (Shimadzu Corporation, Tokyo, Japan). IR spectra were recorded on a Bruker Vertex-70 infrared spectrometer (Bruker Corporation, Bremen, Germany) with KBr pellets. X-ray diffraction data were collected on a Bruker D8 QUEST diffractometer equipped with Helios MX multilayer optics (Bruker AXS, Karlsruhe, Germany) using Cu Kα radiation. NMR spectra were recorded on Bruker AM-500 or AM-800 MHz spectrometers (Bruker Corporation, Bremen, Germany), using TMS as an internal standard. HRESIMS spectral data was measured using an Agilent 1290 UPLC/6540 Q-TOF mass spectrometer (Agilent Technologies, Santa Clara, CA, USA). Semipreparative HPLC was performed on an Agilent 1260 Infinity II (Agilent Technologies, Waldbronn, Germany) equipped with a YMC-Pack ODS-A column (250 × 10 mm, 5 μm). Column chromatography was performed on Sigel (300–400 mesh, Qingdao Marine Chemical Inc., Qingdao, China), RP-18 (50 μm, Merck, Darmstadt, Germany), and Sephadex LH-20 (GE Healthcare, Chicago, IL, USA).

### 3.3. Extraction and Isolation

The air-dried stems and leaves of 40 kg of *D. macropodum Miq*. were extracted with 95% methanol (100 L × 3) at room temperature to obtain the crude extract (1.0 kg). This extract was then suspended in water, and the pH was adjusted to 2–3 using 3% hydrochloric acid. The EtOAc-extracted acidic phase was adjusted to pH 9–10 with 5% NaOH and extracted with CH_2_Cl_2_ to produce 260 g of alkaloid residue. Subsequent normal-phase silica gel chromatography (300–400 mesh; CH_2_Cl_2_/MeOH, 1:0–0:1) of this residue yielded five major fractions (Frs. 1–5). Fraction 1 (16 g) was further separated on a YMC RP-18 column with a MeOH/H_2_O gradient (1:1 → 1:0) to yield four subfractions (Frs. 1A−1D). Fr. 1A was then subjected to normal-phase silica gel (300–400 mesh; PE/EtOAc, 9:1), affording Frs. 1D1−1D4. From Fr. 1D4 (20 mg), compound **1** (3.0 mg, t_R_ = 22 min) was obtained by semipreparative HPLC (MeCN/H_2_O, 46:54, 3.0 mL/min). Fr. 1C (2.0 g) was purified by normal-phase silica gel (300–400 mesh; PE/EtOAc, 1:0 → 0:1), yielding compounds **3** (5.0 mg) and **4** (6.0 mg). Fr. 1D1 (20.0 mg) was eluted through a Sephadex LH-20 column with 100% MeOH to give compound **2** (7.0 mg). Fr. 4 (20 g) was chromatographed on a silica gel column using petroleum ether–acetone (30:1, *v*/*v*) as the eluent, producing four subfractions (Frs. 4A−4D). Finally, Fr. 4C was purified by silica gel CC (300–400 mesh; CH_2_Cl_2_/MeOH, 10:1) to afford compound **5** (4.0 mg).

### 3.4. Identification of New Compounds

#### 3.4.1. Daphmacropomine A (1)

Colorless crystals (DMSO); m.p. 250–252 °C; ${\left[\alpha \right]}_{D}^{20}$ +8.8 (c 0.13, MeOH); UV (MeOH) λ_max_ (log ε) 210 (3.89) nm; IR (KBr) ν_max_: 3426, 2956, 2926, 2854, 1733, 1630, 1384 cm^−1^; ^1^H and ^13^C NMR data, see Table 1; HRESI-TOFMS *m*/*z* 435.1891 [M + Na]^+^ (calcd. for C_23_H_28_N_2_O_5_Na^+^, 435.1890).

#### 3.4.2. Daphmacropomine B (2)

Colorless crystals (CH_2_Cl_2_-MeOH 4:1, *v*/*v*); m.p. 240–242 °C; ${\left[\alpha \right]}_{D}^{25}$ −5.1 (c 0.06, MeOH); UV (MeOH) λ_max_ (log ε) 332 (4.11) nm; IR (KBr) ν_max_: 3433, 2925, 2855, 1693, 1640, 1139 cm^−1^; ^1^H and ^13^C NMR data, see Table 1; HRESI-TOFMS *m*/*z* 430.1858 [M + H]^+^ (calcd. for C_23_H_28_NO_7_^+^, 430.1860).

#### 3.4.3. Daphmacropomine C (3)

White, amorphous powder; ${\left[\alpha \right]}_{D}^{20}$ −8.3 (c 0.07, MeOH); UV (MeOH) λ_max_ (log ε) 195 nm (3.61), 286 nm (2.69); ECD (MeOH) λ (Δε) 213 (−0.71), 286 (0.15); IR (KBr) ν_max_: 3433, 2962, 2924, 1786, 1786, 1371, 1249, 1046 cm^−1^; ^1^H and ^13^C NMR data, see Table 1; HRESI-TOFMS *m*/*z* [M + H]^+^ 474.2490 (calcd. for C_26_H_36_NO_7_^+^, 474.2486).

#### 3.4.4. Daphmacropomine D (4)

White, amorphous powder; ${\left[\alpha \right]}_{D}^{20}$ +4.0 (c 0.15, MeOH); UV (MeOH) λ_max_ (log ε) 195 nm (3.74), 239 nm (3.82), 303 nm (2.88); ECD (MeOH) λ (Δε) 209 (−2.51), 247 (1.10); IR (KBr) ν_max_: 3440, 2932, 1743, 1633, 1451, 1373, 1246, 1039cm^−1^; ^1^H and ^13^C NMR data, see Table 2; HRESI-TOFMS *m*/*z* [M + H]^+^ 460.2333 (calcd. for C_25_H_34_NO_7_^+^, 460.2330).

#### 3.4.5. Daphmacropomine E (5)

White, amorphous powder; ${\left[\alpha \right]}_{D}^{20}$ −169.0 (c 0.10, MeOH); UV (MeOH) λmax (log ε) 195 nm (4.21), 239 nm (3.75); ECD (MeOH) λ (Δε) 213 (14.54), 242 (−42.90); IR (KBr) ν_max_: 3515, 3439, 2947, 1743, 1710, 1637, 1440, 1368, 1306, 1264, 1239, 1204, 1181, 1039 cm^−1^; ^1^H and ^13^C NMR data, see Table 2; HRESI-TOFMS *m*/*z* [M + H]^+^ 472.2333 (calcd. for C_26_H_34_NO_7_^+^, 472.2330).

### 3.5. Crystallographic Data for Compounds ***1*** and ***2***

Colorless crystals of **1** were generated from DMSO under programmed cooling conditions (4 °C, 6 h). Colorless crystals of **2** were formed from a solvent mixture (CH_2_Cl_2_-MeOH 4:1, *v*/*v*) under programmed cooling conditions (4 °C, 6 h). The X-ray crystal structures for daphmacropomine A (**1**) and daphmacropomine B (**2**) were generated using the same instrument and conditions described in Section 3.2. Their structures were solved by direct methods (SHELXT 2014/5), expanded using different Fourier techniques, and refined by the program and full-matrix least-squares calculations. The crystallographic data for compounds **1** and **2** have been deposited in the Cambridge Crystallographic Data Centre (deposition numbers: CCDC 2,296,227 for **1**, and CCDC 2,296,251 for **2**). Copies of the data can be obtained, free of charge, upon application to the CCDC, 12 Union Road, Cambridge CB2 1EZ, UK (fax: +44(0)-1223-336033 or e-mail: deposit@ccdc.cam.ac.uk).

X-ray Crystallographic Analysis of **1.** Crystallographic Data for compound **1**. 3(C_23_H_28_N_2_O_5_)•2(C_2_H_6_OS), *M* = 1393.67, *a* = 17.7509(9) Å, *b* = 17.7509(9) Å, *c* = 18.6508(10) Å, *α* = 90°, *β* = 90°, *γ* = 120°, *V* = 5089.4(6) Å^3^, *T* = 100.(2) K, space group *R*3, *Z* = 3, *μ*(Cu K*α*) = 1.342 mm^−1^, 22,806 reflections measured, 4194 independent reflections (*R_int_* = 0.0463). The final *R_1_* values were 0.0339 (*I* > 2*σ*(*I*)). The final *wR*(*F*^2^) values were 0.0880 (*I* > 2*σ*(*I*)). The final *R_1_* values were 0.0352 (all data). The final *wR*(*F*^2^) values were 0.0895 (all data). The goodness of fit on *F*^2^ was 1.041. Flack parameter = 0.062(12).

X-ray Crystallographic Analysis of **2.** Crystallographic Data for compound **2**. C_23_H_27_NO_7_, *M* = 429.45, *a* = 9.8294(4) Å, *b* = 10.0193(4) Å, *c* = 20.7668(8) Å, *α* = 90°, *β* = 90°, *γ* = 90°, *V* = 2045.19(14) Å^3^, *T* = 100.(2) K, space group *P*212121, *Z* = 4, *μ*(Cu K*α*) = 0.858 mm^−1^, 34,648 reflections measured, 4035 independent reflections (*R_int_* = 0.0469). The final *R_1_* values were 0.0276 (*I* > 2*σ*(*I*)). The final *wR*(*F*^2^) values were 0.0727 (*I* > 2*σ*(*I*)). The final *R_1_* values were 0.0278 (all data). The final *wR*(*F*^2^) values were 0.0729 (all data). The goodness of fit on *F*^2^ was 1.036. Flack parameter = 0.09(4).

### 3.6. Computational Methods of ECD

Conformational searches for compounds **3**, **4**, and **5** were conducted using CONFLEX based on MMFF94S molecular mechanical force fields. Conformers with populations above 1% were selected and further optimized at the B3LYP/6-31G* level via density functional theory (DFT) using the Gaussian 09 software package. The optimized geometries were verified by frequency analysis, which confirmed the absence of imaginary frequencies. ECD calculations were performed using the TD-DFT method at the B3LYP/6-31G+(d,p) level on the B3LYP/6-31G(d)-optimized geometries with the IEFPCM solvation model (in MeOH). The final calculated ECD spectrum was generated using SpecDis 1.604 with a half-bandwidth (σ) of 0.30 eV.

### 3.7. Evaluation of the Bioactivity

Compounds **1**−**4** were evaluated for inducing lysosomal biogenesis and promoting autophagy. Experimental procedures are provided in the Supplementary Materials.

## 4. Conclusions

Overall, an investigation of *D. macropodum* Miq. stems and leaves led to the isolation of five new alkaloids. Daphmacropomin A (**1**) is the first example of a *Daphniphyllum* alkaloid that features an unprecedented 6/9/7/5/5 ring with a rare *N*-nitroso group. Daphmacropomine B (**2**) is the second *Daphniphyllum* alkaloid and features a highly rearranged 6/6/6/7/5/6-fused skeleton. Notably, daphmacropomines C (**3**) and D (**4**) could induce slight lysosomal biogenesis and promote autophagic flux. This work expands the chemical understanding of structural diversity within the *Daphniphyllum* alkaloids.

## Figures and Tables

**Table 1:** ^1^H and ^13^C NMR data of alkaloids **1**−**3** (*δ* in ppm, *J* in Hz).

Position	1 *^a^*		2 *^b^*		3 *^b^*	
No.	*δ*_H_	*δ*_C_	*δ*_H_	*δ*_C_	*δ*_H_	*δ*_C_
1		206.7		78.8	2.82, d (2.4)	67.5
2	3.26, t (3.9, 1.9)	49.5		210.9	2.08, m	37.2
3a	6.23, dd (10.2, 4.1)	128.2	3.21, d (15.5)	47.8	1.41, m	28.4
3b			2.50, d (15.5)		2.11, m	
4	6.36, dd (10.1, 1.4)	131.8		207.7	5.55, m	70.6
5		60.2		49.7		43.5
6	2.75, m	47.3	3.38, dd (17.5, 9.8)	40.6	2.36, t (6.3)	36.6
7a	3.92, dd (14.8, 3.0)	59.1	2.40, m	53.4	3.07, m	57.5
7b	4.51, dd (14.9, 3.2)		2.42, m		2.97, m	
8		58.7		82.2		46.7
9		140.5		111.9		111.0
10		139.8		158.8		76.7
11a	1.89, dd (24.6, 12.2)	31.0	2.20, dd (17.0, 7.7)	27.6	2.10, m	34.1
11b	1.85, m		2.98, m		1.98, m	
12a	2.23, m	26.9	1.51, m	23.8	1.48, m	27.2
12b	2.07, m		1.96, m		1.97, m	
13a	2.46, d (6.3)	42.1		76.2	3.04, m	38.7
13b	2.52, dd (15.1, 8.7)				1.83, m	
14	2.91, m	41.5		124.1	2.78, d (3.0)	45.6
15	3.49, m	53.7		157.3	3.06, m	52.2
16a	1.31, td (9.3, 3.3)	28.0	2.97, m	29.0	1.82, m	20.1
16b	2.01, m		3.32, dd (20.0, 3.9)		1.40, m	
17a	2.40, m	39.8	3.89, m	65.5	2.27, m	45.5
17b	2.45, m		4.33, dd (10.8, 5.9)		2.27, m	
18	3.00, m	31.9	2.64, m	42.8	2.18, m	38.4
19a	2.60, m	53.9	2.26, d (12.4)	53.8	3.17, dd (12.0, 10.0)	61.9
19b	3.86, dd (13.0, 4.5)		3.10, dd (12.3, 4.5)		2.03, dd (12.0, 10.0)	
20	1.08, d (7.2)	16.9	1.35, d (7.8)	19.7		14.5
21a	10.03, s	205.5	1.31, s	15.0	4.39, d (8.4)	67.4
21b					5.13, d (8.4)	
22		175.0		167.5		176.3
23	3.66, s	51.8	3.65, s	50.9		
4-OAc					2.05, s	20.9 170.1
21-OAc					2.11, s	21.0 170.5
OH-1			4.52, s			
OH-8			4.38, s			
OH-10					8.91, s	

*^a^* 800 MHz (^1^H) and 200 MHz (^13^C) in DMSO-*d*_6_. *^b^* 500 MHz (^1^H) and 125 MHz (^13^C) in CDCl_3_.

**Table 2:** ^1^H and ^13^C NMR data of alkaloids **4**–**5** (*δ* in ppm, *J* in Hz).

	4 *^a^*		5 *^a^*	
No.	*δ*_H_	*δ*_C_	*δ*_H_	*δ*_C_
1		94.0		183.3
2	2.19, m	44.3		57.5
3a	2.23, m	28.5	2.21, m	32.7
3b	1.63, m		2.51, dd (6.1, 13.2)	
4	5.80, dd (10.8, 7.8)	71.2	5.37, dd (6.2, 9.7)	72.1
5		46.0		51.6
6	2.50, m	35.8	2.87, m	44.1
7a	3.31, m	59.2	4.38, d (5.9)	66.6
7b	3.22, d (12.3)			
8		54.8		45.8
9		184.1		132.3
10		82.2		142.7
11a	2.72, m	35.9	2.28, m	25.6
11b	1.99, m		2.00, m	
12a	1.57, m	26.7	2.19, m	22.3
12b	1.57, m		1.97, m	
13a	3.38, d (17.2)	48.6	3.40, dd (4.5, 13.5)	39.5
13b	3.29, d (17.2)		2.80, dd (4.5, 13.5)	
14		202.2	3.57, m	43.6
15		153.8	3.96, m	54.4
16a	2.54, m	23.0	1.84, m	27.7
16b	2.33, m		1.97, m	
17a	2.45, dd (11.9, 7.3)	44.5	2.37, m	43.1
17b	2.32, dd (11.9, 7.3)		2.22, m	
18	2.95, m	35.5	2.17, m	37.8
19a	3.70, t (9.8)	63.3	4.06, dd (15.4, 7.1)	68.5
19b	2.22, m		3.57, dd (15.4, 7.1)	
20	0.95, d (7.3)	14.4	1.21, d (7.2)	16.6
21a	4.11, d (11.9)	65.6	4.88, d (11.5)	65.2
21b	4.40, d (11.9)		4.67, d (11.5)	
22				177.7
4-OAc	2.09, s	21.0 170.2	2.02, s	20.9 170.7
21-OAc	2.09, s	21.1 170.4	1.95, s	20.8 170.2
OH-1	3.26, s			

*^a^* 500 MHz (^1^H) and 125 MHz (^13^C) in C_5_D_5_N.

## Data Availability

Supplementary Materials for this study can be obtained by contacting the corresponding authors via e-mail.

## Supplementary Materials

The following supporting information can be downloaded at https://www.mdpi.com/article/10.3390/molecules31111943/s1, Figures S1–S9: 1D/2D NMR, HR-ESIMS, IR, and UV spectra of compound **1**; Figures S10–S18: 1D/2D NMR, HR-ESIMS, IR, and UV spectra of compound **2**; Figures S19–S27: 1D/2D NMR, HR-ESIMS, IR, and UV spectra of compound **3**; Figure S28: ECD spectrum of compound **3**; Figures S29–S38: 1D/2D NMR, HR-ESIMS, IR, and UV spectra of compound **4**; Figures S39–S48: 1D/2D NMR, HR-ESIMS, IR, and UV spectra of compound **5**; Figure S49: View of the hydrogen-bonded motif of compound **1**; Figure S50: View of the hydrogen-bonded motif of compound **2**. References [22,23,24] have been cited in Supplementary Materials.
