# Supplementary material for: Daphmacropomines A–E: Five Daphniphyllum Alkaloids from Daphniphyllum macropodum Miq
Source: Molecules. 2026 Jun 3;31(11):1943. doi: 10.3390/molecules31111943 (PMC13258748; doi:10.3390/molecules31111943)
Supplement: Supplementary file 1 [file molecules-31-01943-s001.zip › SI.pdf]

Supporting Information (SI)

Daphmacropomines A–E, five *Daphniphyllum* alkaloids from *Daphniphyllum macropodum* Miq.

Lili Xu,[1,2] Zhenpeng Niu,[1,2] Yu Zhang,[1,2] Hong Liang,[1,2] Qian Zhao,[1,2] Sheng Li,[1,2] Duozhi Chen,[1,2] Xiao Ding,[1,2,3,4*] Xiaojiang Hao[1,2,3,4,5*]

[1] State Key Laboratory of Phytochemistry and Plant Resources in West China, Kunming Institute of Botany, Chinese Academy of Sciences, Kunming, 650201, China

[2] University of Chinese Academy of Sciences, Beijing, 100049, China

[3] Research Unit of Chemical Biology of Natural Anti-Virus Products, Chinese Academy of Medical Sciences, Beijing, 100730, China

[4] Yunnan Characteristic Plant Extraction Laboratory, Kunming, 650106, China

[5] Xinjiang Technical Institute of Physics and Chemistry, Chinese Academy of Sciences, Urumqi, 830011, China

To whom correspondence should be addressed. Tel: +86-871-65223263. Fax: +86-871-65223070.

E-mail: haoxj@mail.kib.ac.cn; dingxiao@mail.kib.ac.cn.

List of Supporting Information

Evaluation of the bioactivity S4
Figure S1 ^{1}H NMR (800MHz) spectrum of compound **1** in DMSO-d6 .... S6
Figure S2 . ^{13}C NMR (200 MHz) spectrum of compound **1** in DMSO-d_6 .... S7
Figure S3 HSQC (800 MHz) spectrum of compound **1** in DMSO-d_6 .... S8
Figure S4 ^{1}H-^{1}H COSY (800 MHz) spectrum of compound **1** in DMSO-d_6 .... S9
Figure S5 HMBC (800 MHz) spectrum of compound **1** in DMSO-d_6 .... S10
Figure S6 ROESY (800 MHz) spectrum of compound **1** in DMSO-d_6 .... S11
Figure S7 HR-ESI-MS spectrum of compound **1** S12
Figure S8 IR (KBr disk) spectrum of compound **1** S13
Figure S9 UV spectrum of compound **1** S14
Figure S10 ^{1}H NMR (500MHz) spectrum of compound **2** in $CDCl_3$ S15
Figure S11 ^{13}C NMR (125MHz) spectrum of compound **2** in $CDCl_3$ S16
Figure S12 HSQC (500 MHz) spectrum of compound **2** in $CDCl_3$ S17
Figure S13 ^{1}H-^{1}H COSY (500 MHz) spectrum of compound **2** in $CDCl_3$ S18
Figure S14 HMBC (500 MHz) spectrum of compound **2** in $CDCl_3$ S19
Figure S15 ROESY (500 MHz) spectrum of compound **2** in $CDCl_3$ S20
Figure S16 HR-ESI-MS spectrum of compound **2** S21
Figure S17 IR (KBr disk) spectrum of compound **2** S22
Figure S18 UV spectrum of compound **2** S23
Figure S19 ^{1}H NMR (500MHz) spectrum of compound **3** in C_5D_5N S24
Figure S20 ^{13}C NMR (125 MHz) spectrum of compound **3** in C_5D_5N S25
Figure S21 HSQC (500 MHz) spectrum of compound **3** in C_5D_5N S26
Figure S22 ^{1}H-^{1}H COSY (500 MHz) spectrum of compound **3** in C_5D_5N S27
Figure S23 HMBC (500 MHz) spectrum of compound **3** in C_5D_5N S28
Figure S24 ROESY (500 MHz) spectrum of compound **3** in C_5D_5N S29
Figure S25 HR-ESI-MS spectrum of compound **3** S30
Figure S26 IR (KBr disk) spectrum of compound **3** S31
Figure S27 UV spectrum of compound **3** S32
Figure S28 ECD spectrum of compound **3** S33
Figure S29 ^{1}H NMR (500MHz) spectrum of compound **4** in C_5D_5N S34
Figure S30 ^{13}C NMR (125 MHz) spectrum of compound **4** in C_5D_5N S35

Figure S31 HSQC (500 MHz) spectrum of compound **4** in C_5D_5N S36

Figure S32 ^{1}H-^{1}H COSY (500 MHz) spectrum of compound **4** in C_5D_5N S37

Figure S33 HMBC (500 MHz) spectrum of compound **4** in C_5D_5N S38

Figure S34 ROESY (500 MHz) spectrum of compound **4** in C_5D_5N S39

Figure S35 HR-ESI-MS spectrum of compound **4** S40

Figure S36 IR (KBr disk) spectrum of compound **4** S41

Figure S37 UV spectrum of compound **4** S42

Figure S38 ECD spectrum of compound **4** S43

Figure S39 ^{1}H NMR (500MHz) spectrum of compound **5** in C_5D_5N S44

Figure S40 ^{13}C NMR (125 MHz) spectrum of compound **5** in C_5D_5N S45

Figure S41 HSQC (500 MHz) spectrum of compound **5** in C_5D_5N S46

Figure S42 ^{1}H-^{1}H COSY (500 MHz) spectrum of compound **5** in C_5D_5N S47

Figure S43 HMBC (500 MHz) spectrum of compound **5** in C_5D_5N S48

Figure S44 ROESY (500 MHz) spectrum of compound **5** in C_5D_5N S49

Figure S45 HR-ESI-MS spectrum of compound **5** S50

Figure S46 IR (KBr disk) spectrum of compound **5** S51

Figure S47 UV spectrum of compound **5** S52

Figure S48 ECD spectrum of compound **5** S53

Figure S49 View of the hydrogen-bonded motif of daphmacropomine A (**1**). S54

Figure S50 View of the hydrogen-bonded motif of daphmacropomine B (**2**). S55

Evaluation of the bioactivity

Cell culture. HepG2 cells were cultured in DMEM, supplemented with 10% FBS at 37℃ in a 5% CO_2 atmosphere.

Transfection of plasmid. Cells were transfected with EGFP-TFE3, TFEB-EGFP, RFP-GFP-LC3, and LAMP1-EGFP using Lipofectamine 3000 (Life Technologies, US) according to the manufacturer's instructions. Subsequently, the cells were incubated for 24 h post transfection and then subjected to confocal microscopy.

Screening for compounds that induce lysosomal biogenesis. HepG2 cells were seeded in 96-well plates and grown to 85% confluence. Next, the cells were treated with 40 μM of the different compounds for 3 h in triplicate, DMSO and Hep14 were used as the negative and positive controls, respectively. After the treatment period, 50 nM LysoTracker Red DND-99 (Thermo Fisher Scientific, L7528) was added to the medium, and the cells were incubated for 30 min.[1, 2] Subsequently, the medium was replaced with fresh medium, and the plates were imaged with Array Scan Infinity (Cellomics, ArrayScan VTI HCS). All compounds were tested at the indicated concentrations in triplicate.

Western blot analysis. Standard western blot analysis was performed following a procedure outlined by Wei et al [3].

Statistical analysis. All the data were analyzed with GraphPad Prism 9.0 (GraphPad Software). The data are presented as the mean ± SD. An unpaired Student's *t*-test (two-sided) was used to compare the compound treatment group with the control group. $p < 0.05$ was considered to indicate statistical significance. $p > 0.05$ was considered not significant (n.s.).

References

1. Niu, Z.P.; Tang, G.H.; Wang, X.N.; Yang, X.; Zhao, Y.Q.; Wang, Y.Y; Liu, Q.; Zhang, F.; Zhao, Y.H.; Ding, X.; Hao, X.J. Trigonochinene E promotes lysosomal biogenesis and enhances autophagy via TFEB/TFE3 in human degenerative NP cells against oxidative stress. *Phytomedicine*, 2023, **112**, 154720.

2. Ding, X; Yang, X.; Zhao, Y.Q.; Wang, Y.Y.; Fei, J.; Niu, Z.P.; Dong, X.X.;Wang, X.N.; Liu, B., Li, H.M.; Hao, X.J.; Zhao, Y.H. Identification of active natural products that induce lysosomal biogenesis by lysosome-based screening and biological evaluation. *Heliyon*. 2022, **8**, e11179.

3. Wei, R.R.; Zhao,Y.Q.; Wang, J.; Yang, X.; Li, S.L.; Wang, Y.Y.; Yang, X.; Fei, J.M.; Hao, X.J.; Zhao, Y.H.; Gui, L.M.; Ding, X. Tagitinin C induces ferroptosis through PERK-Nrf2-HO-1 signaling pathway in colorectal cancer cells. *Int. J. Biol. Sci.* 2021, **17**, 2703–271.

Figure S1 ^{1}H NMR (800MHz) spectrum of compound **1** in DMSO-d_6

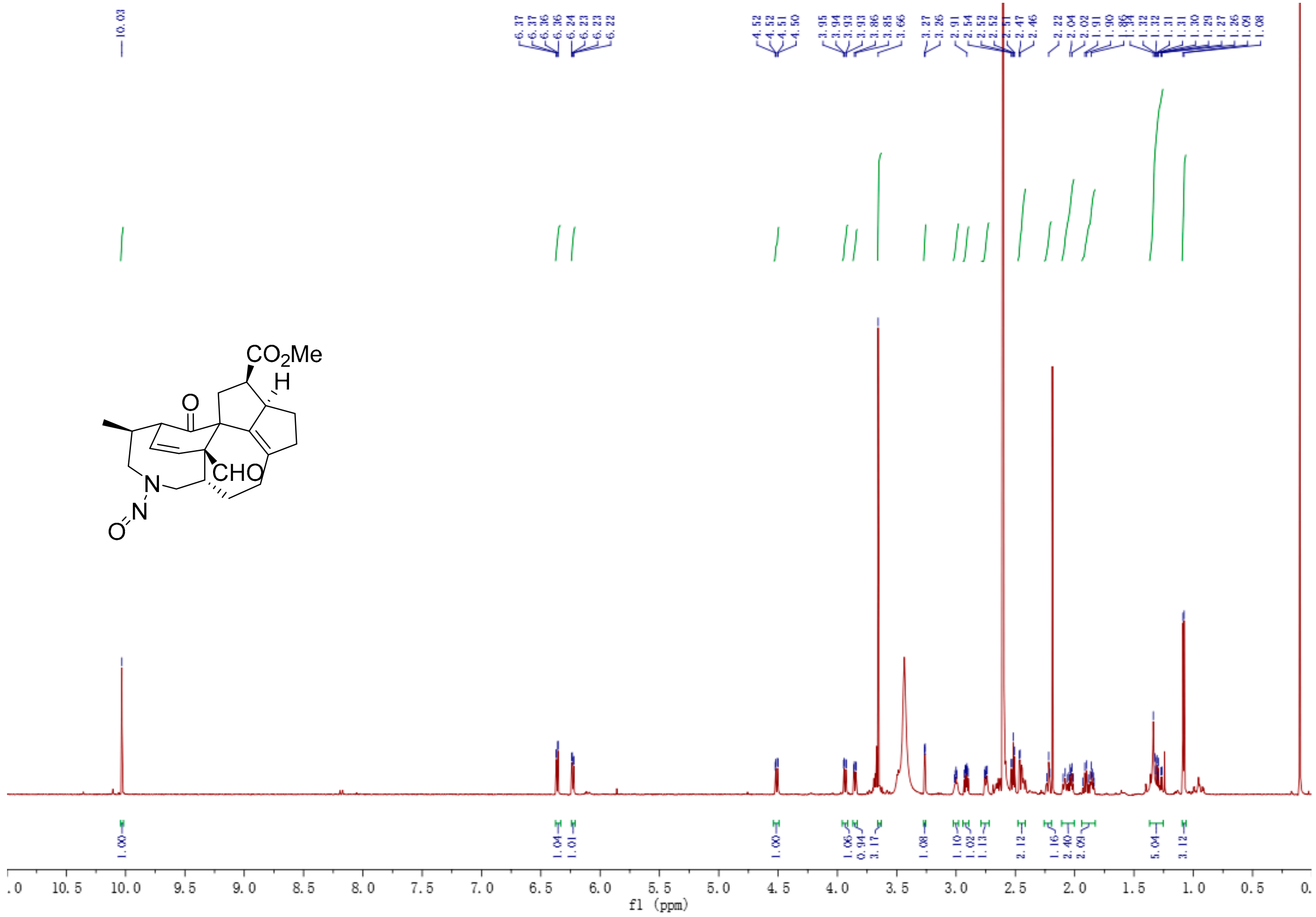

Figure S2 .^{13}C NMR (200 MHz) spectrum of compound **1** in DMSO-d_6

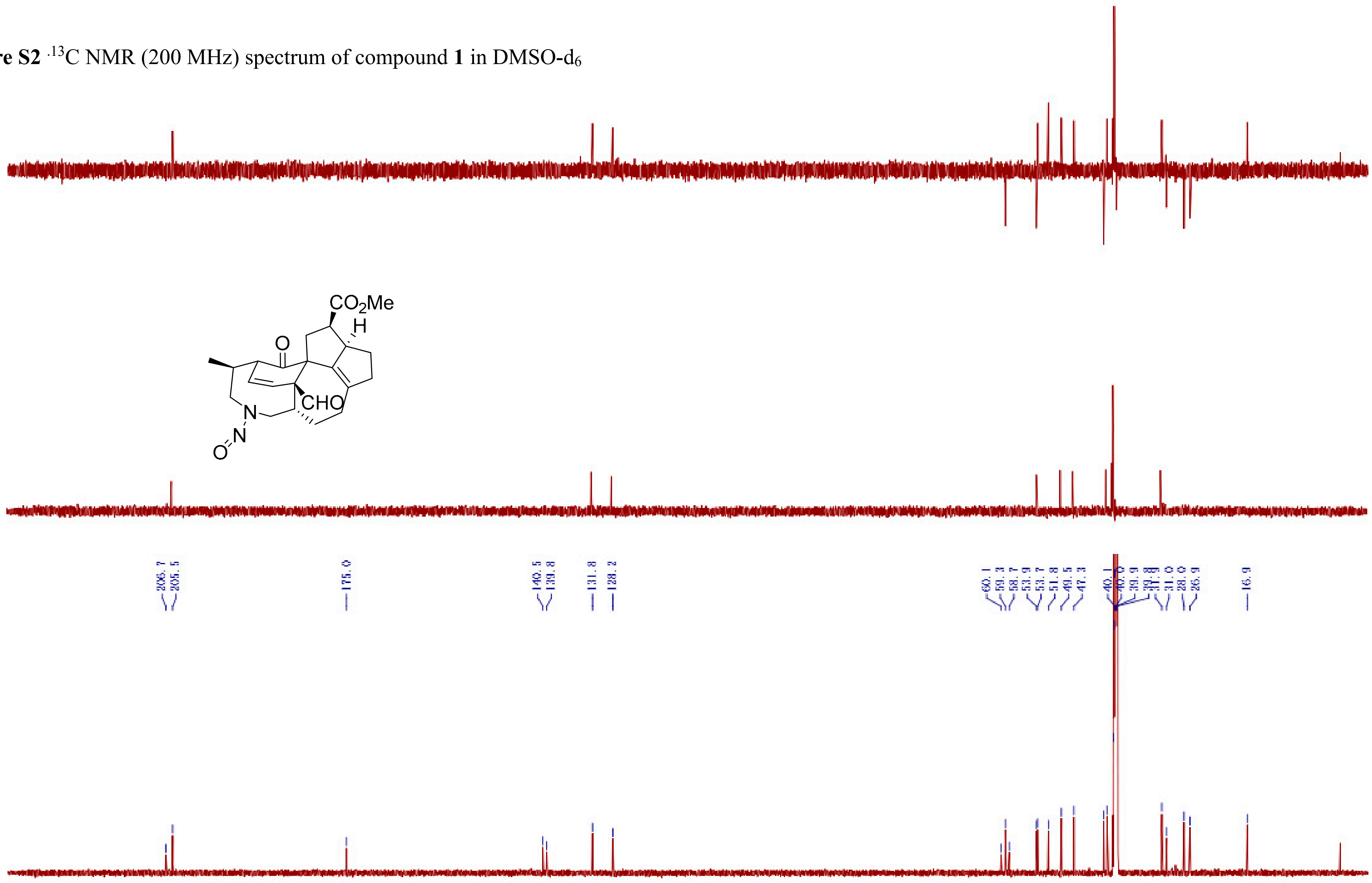

Figure S3 HSQC (800 MHz) spectrum of compound **1** in DMSO-d6

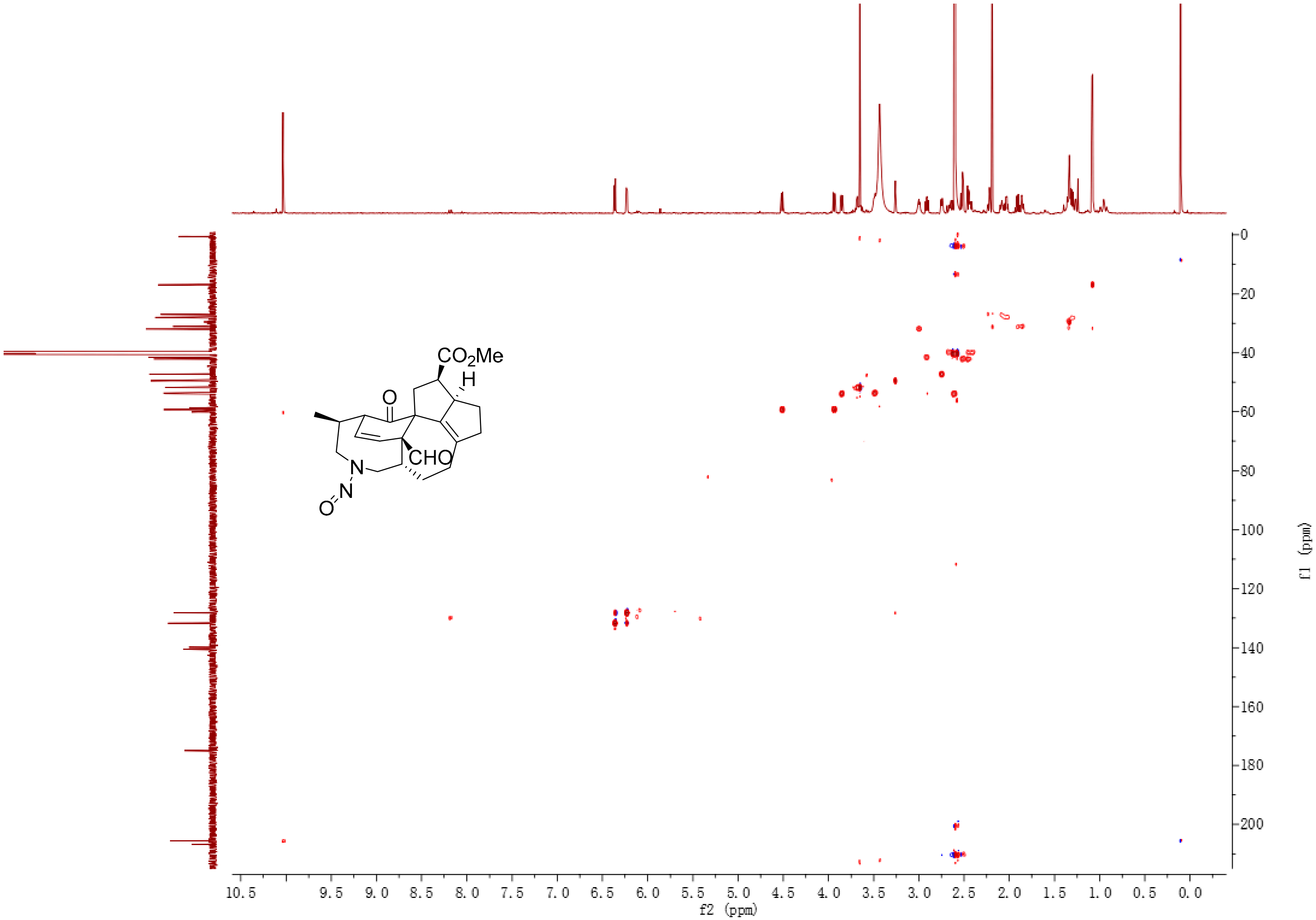

Figure S4 ^{1}H-^{1}H COSY (800 MHz) spectrum of compound **1** in DMSO-d_6

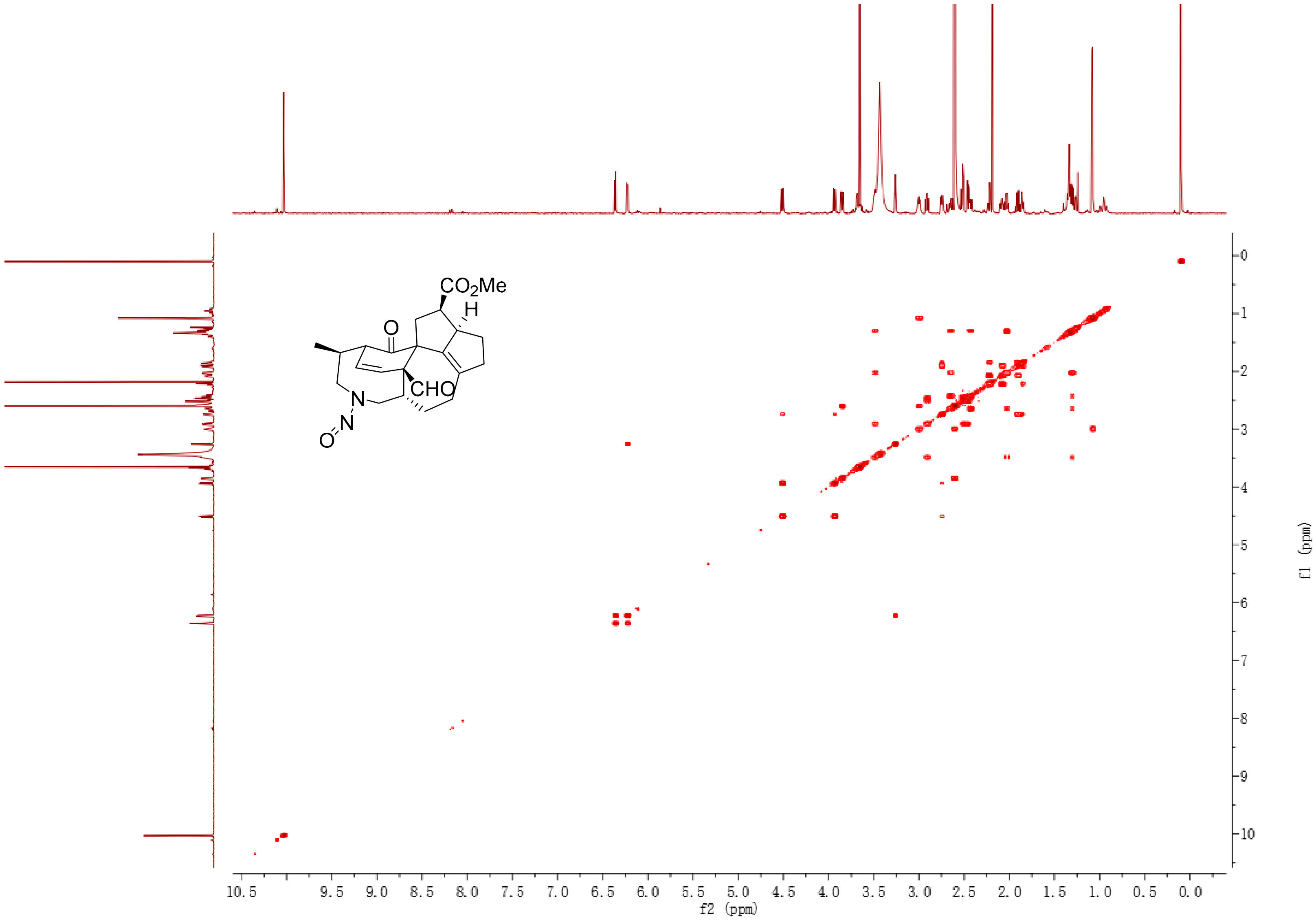

Figure S5 HMBC (800 MHz) spectrum of compound **1** in DMSO-d$_6$

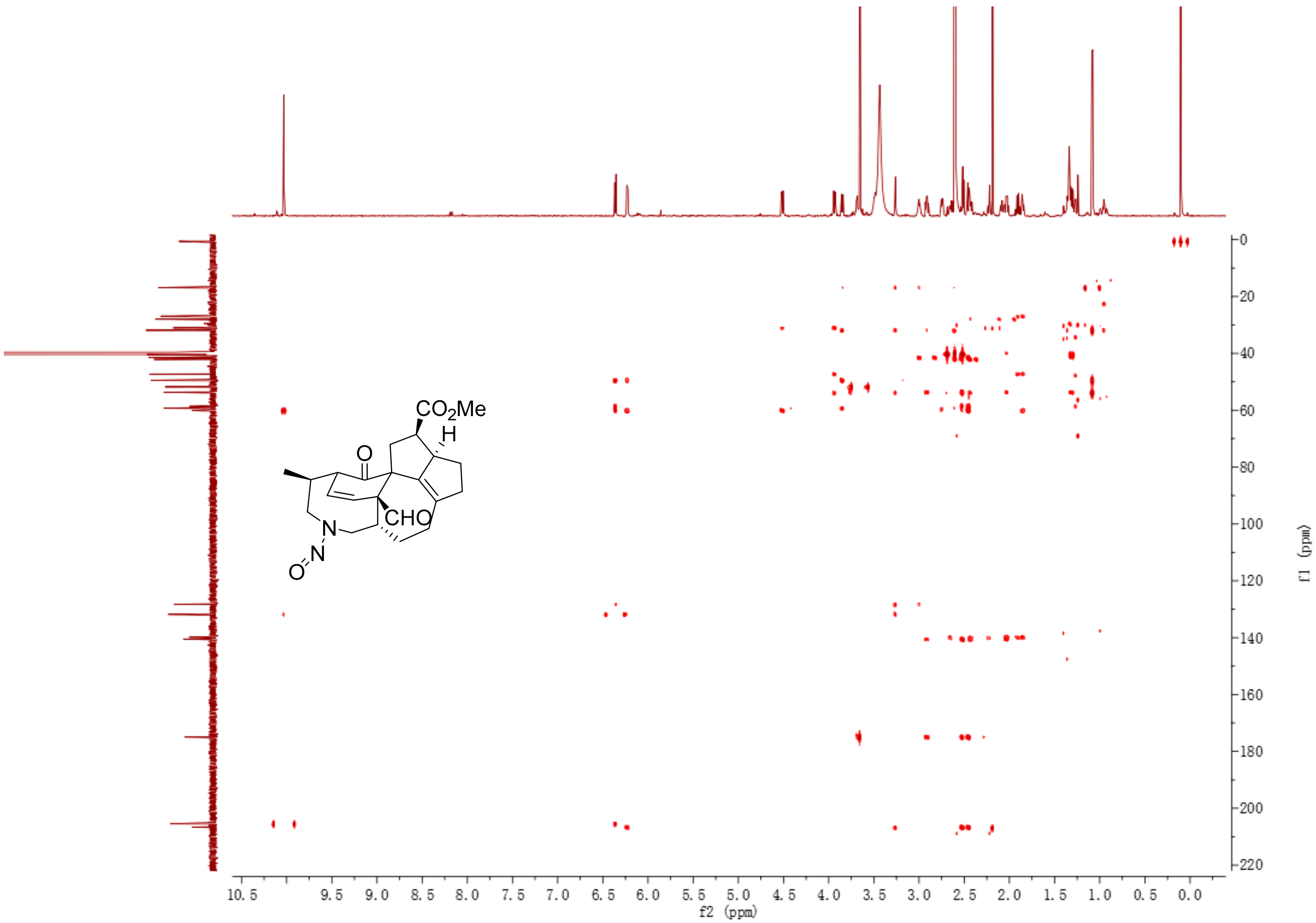

Figure S6 ROESY (800 MHz) spectrum of compound **1** in DMSO-d_6

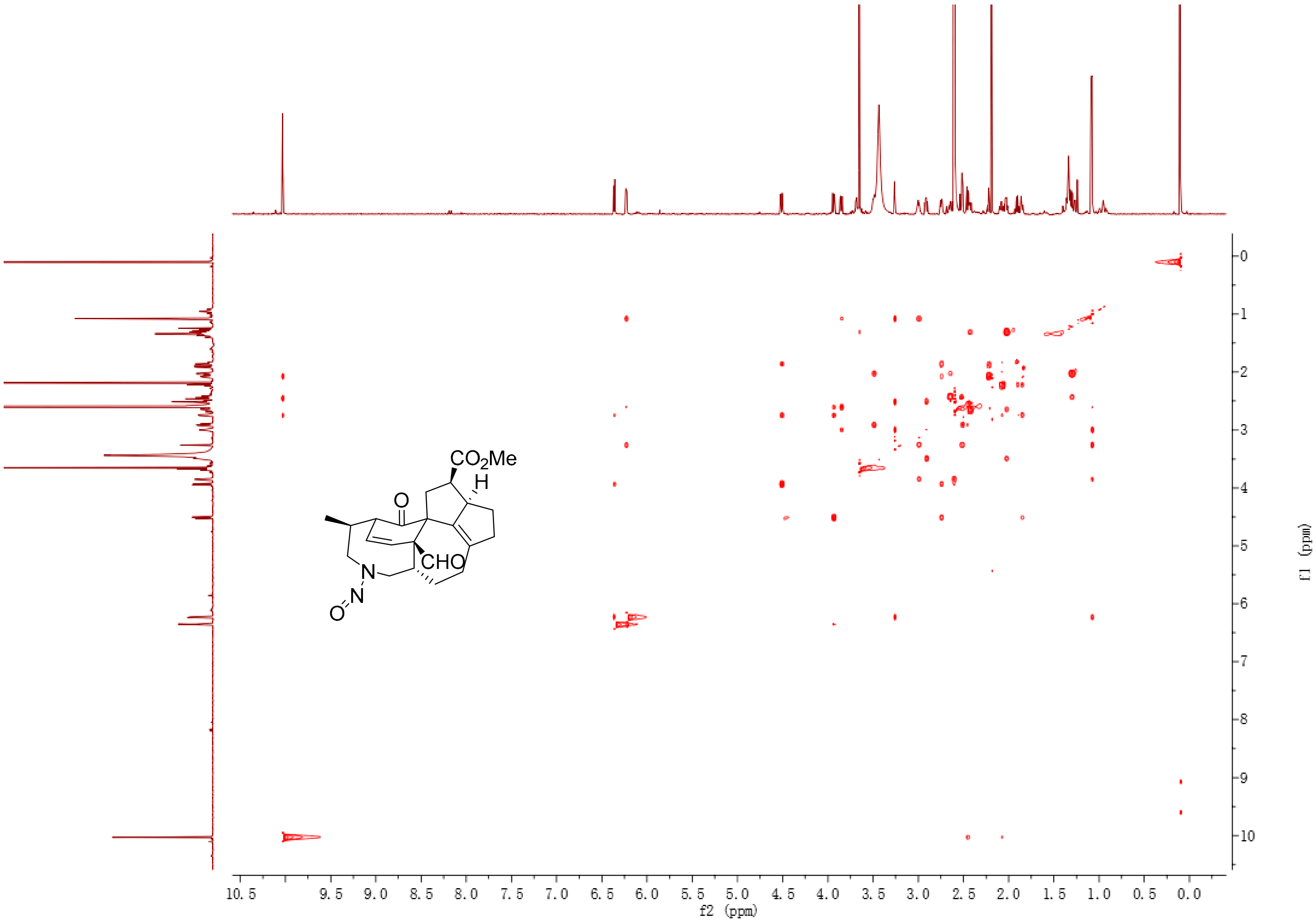

Figure S7 HR-ESI-MS spectrum of compound **1**

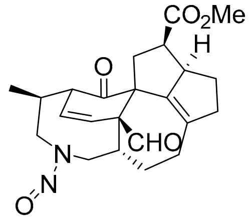

User Spectra

Fragmentor Voltage	Collision Energy	Ionization Mode
135	0	ESI

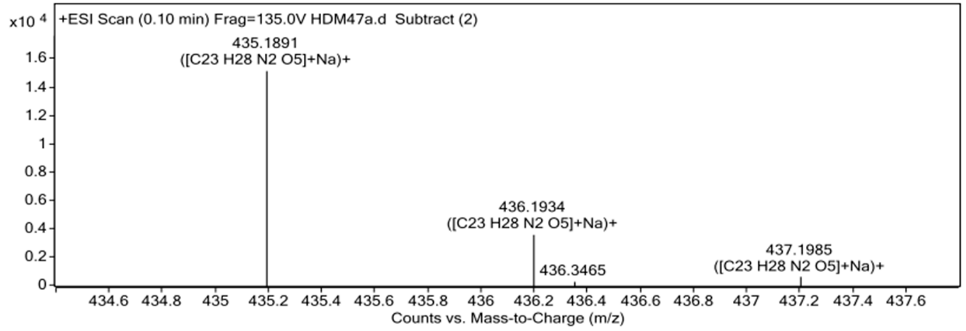

Peak List

m/z	z	Abund	Formula	Ion
383.2091	1	17216.69		
384.2131	1	5847.78		
430.2351	1	5903.47		
435.1891	1	15197.32	C23 H28 N2 O5	(M+Na)+
458.2656	1	53821.99		
459.2682	1	15354.26		
481.2436	1	7769.22		
514.3277	1	10259.23		
515.3317	1	4995.57		
847.3886	1	5453.09		

Formula Calculator Element Limits

Element	Min	Max
C	3	120
H	0	240
O	0	30
N	0	5

Formula Calculator Results

Formula	CalculatedMass	CalculatedMz	Mz	Diff. (mDa)	Diff. (ppm)	DBE
C23 H28 N2 O5	412.1998	435.1890	435.1891	-0.10	-0.23	11.0000

Figure S8 IR (KBr disk) spectrum of compound **1**

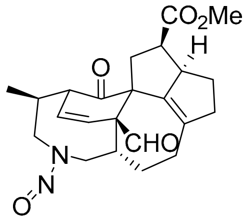

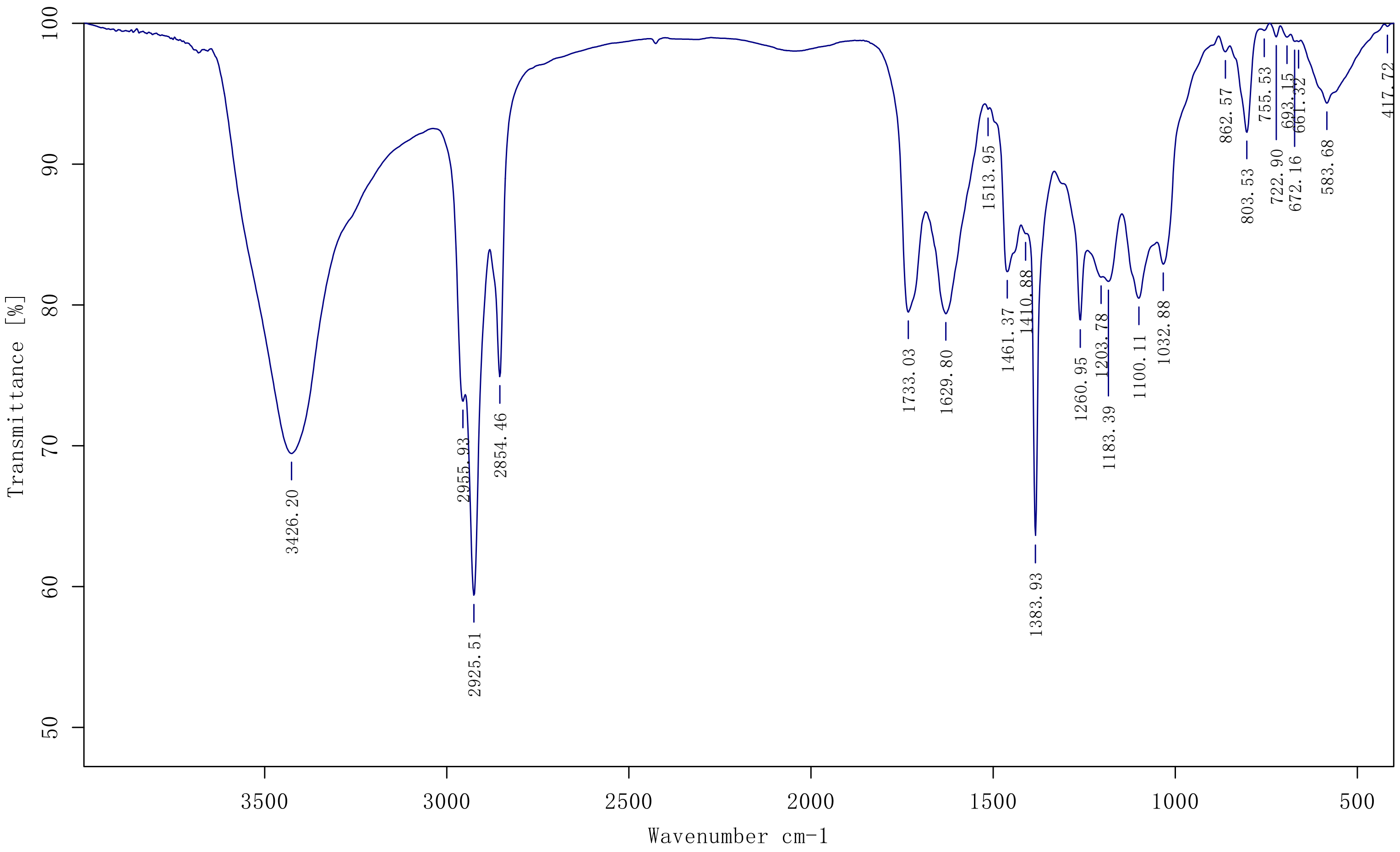

Sample Name: HDM47a
Sample Form: KBr
Path of File: E:\data
Date of Measurement: 2023/5/19

Resolution: 4
Aperture Setting: 6 mm
Number of Background Scans: 16
Number of Sample Scans: 16

Beamsplitter Setting: KBr
Source Setting: MIR
Instrument Type: BRUKER VERTEX 70
Soft Version: OPUS8.1

Figure S9 UV spectrum of compound **1**

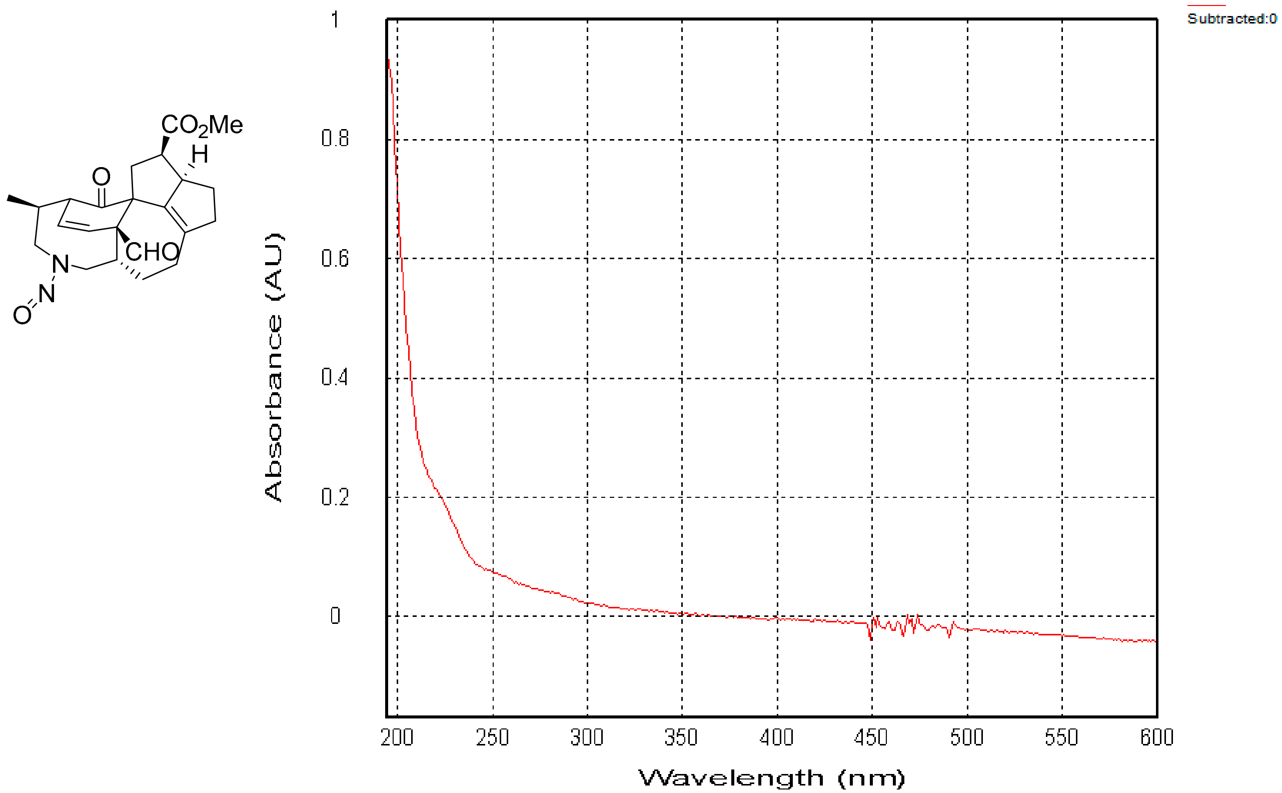

Figure S10 ^{1}H NMR (500MHz) spectrum of compound **2** in $CDCl_3$

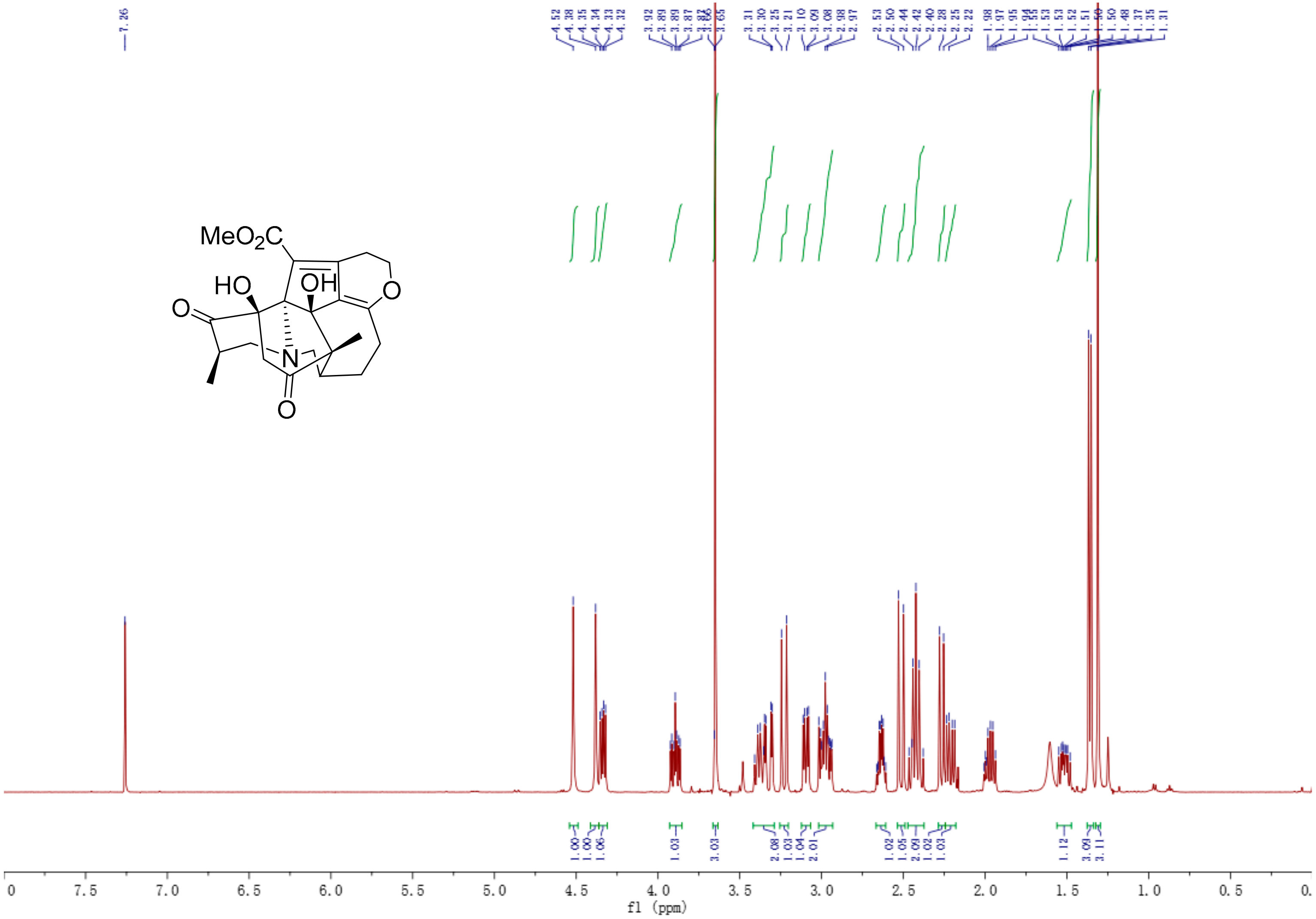

Figure S11 ^{13}C NMR (125MHz) spectrum of compound **2** in $CDCl_3$

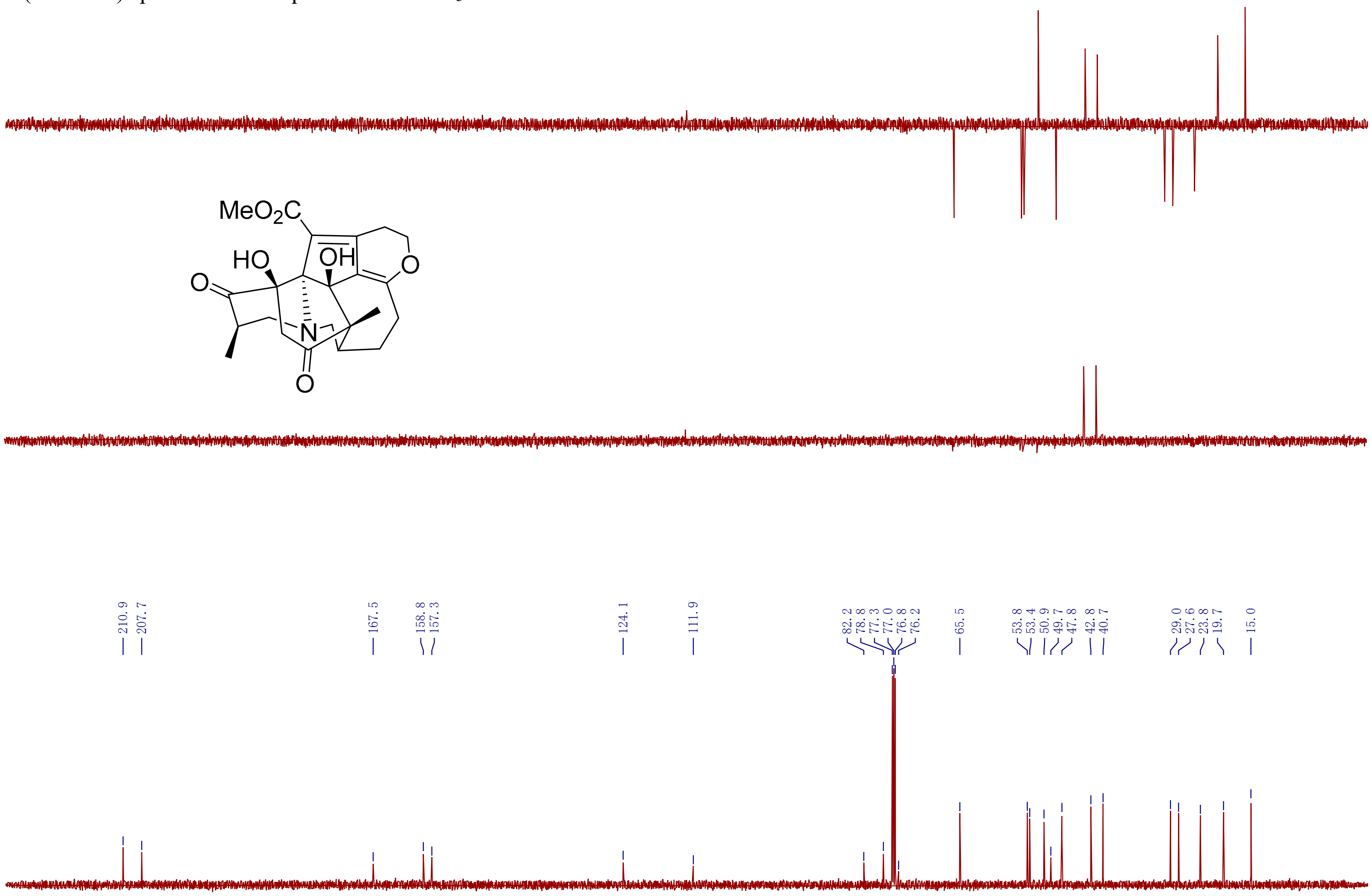

Figure S12 HSQC (500 MHz) spectrum of compound **2** in $CDCl_3$

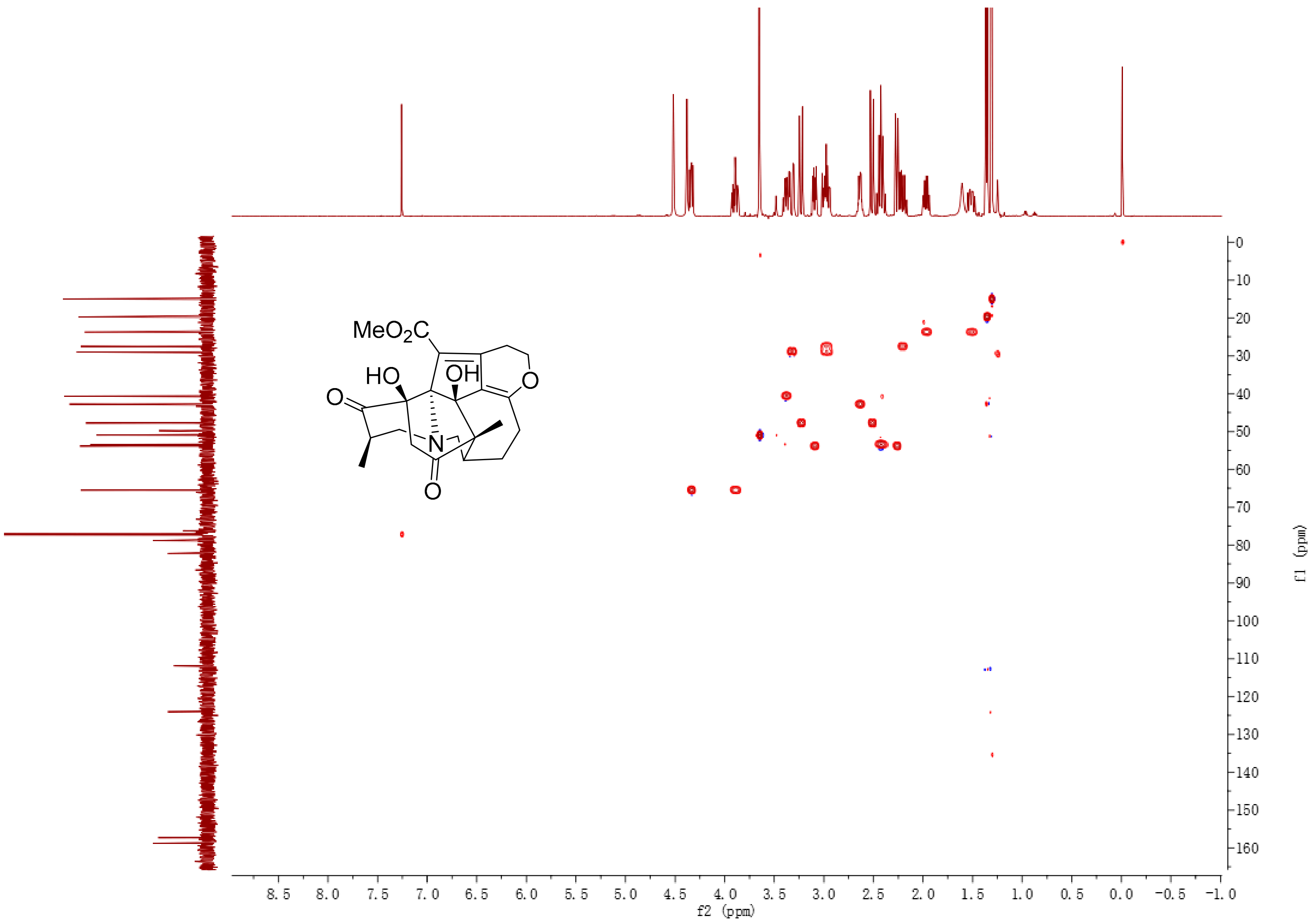

Figure S13 ^1H-^1H COSY (500 MHz) spectrum of compound **2** in $CDCl_3$

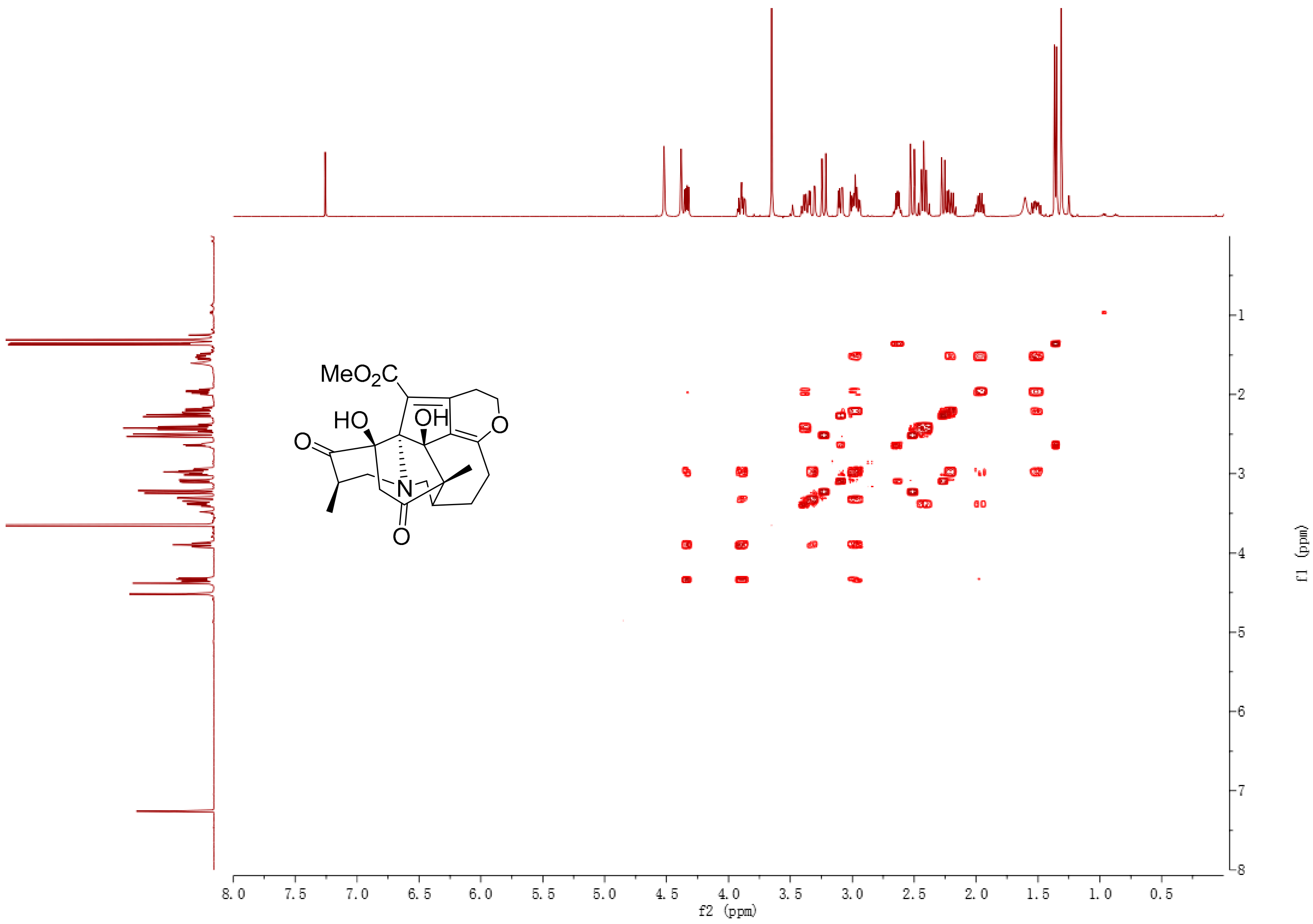

Figure S14 HMBC (500 MHz) spectrum of compound **2** in $CDCl_3$

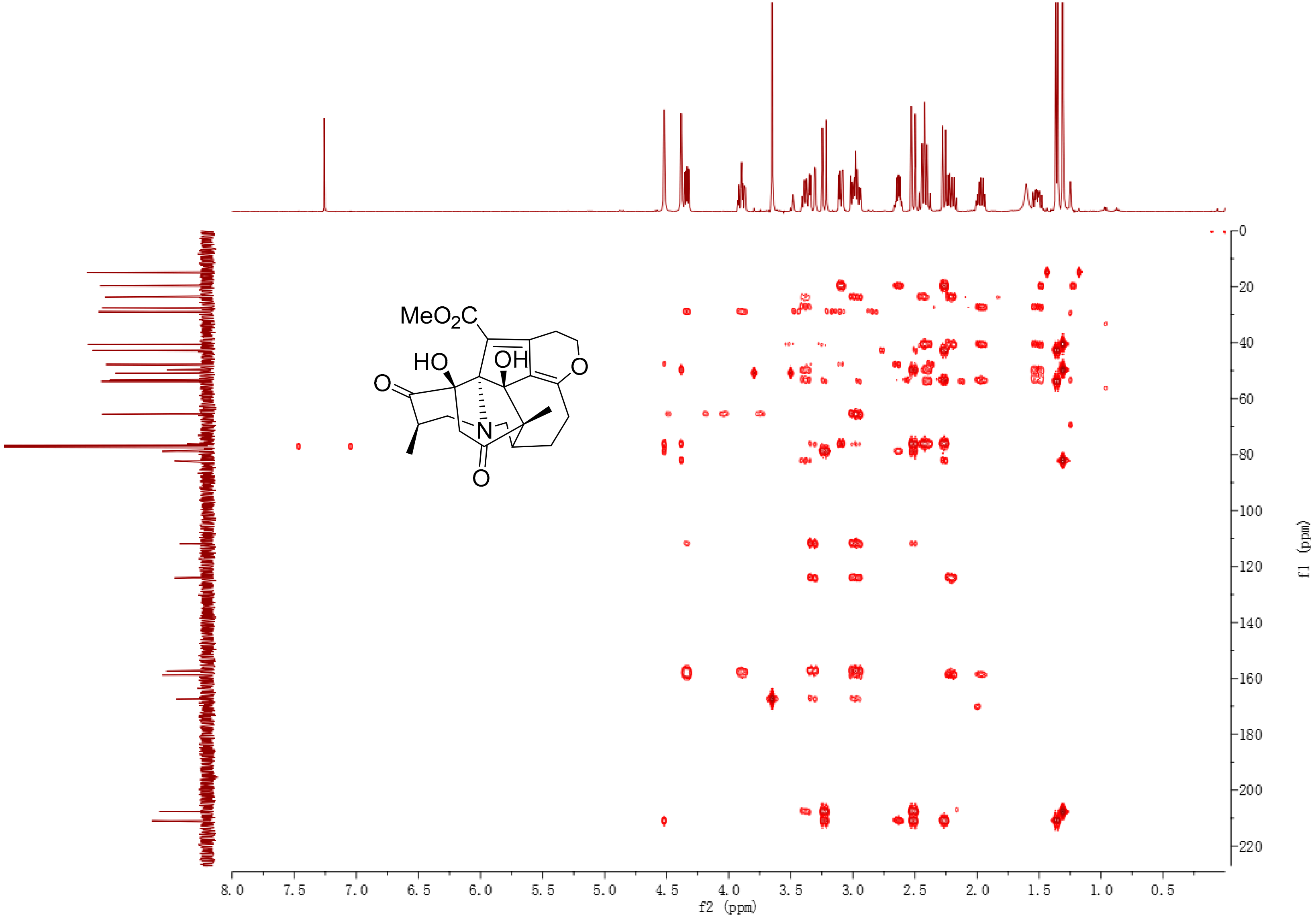

Figure S15 ROESY (500 MHz) spectrum of compound **2** in $CDCl_3$

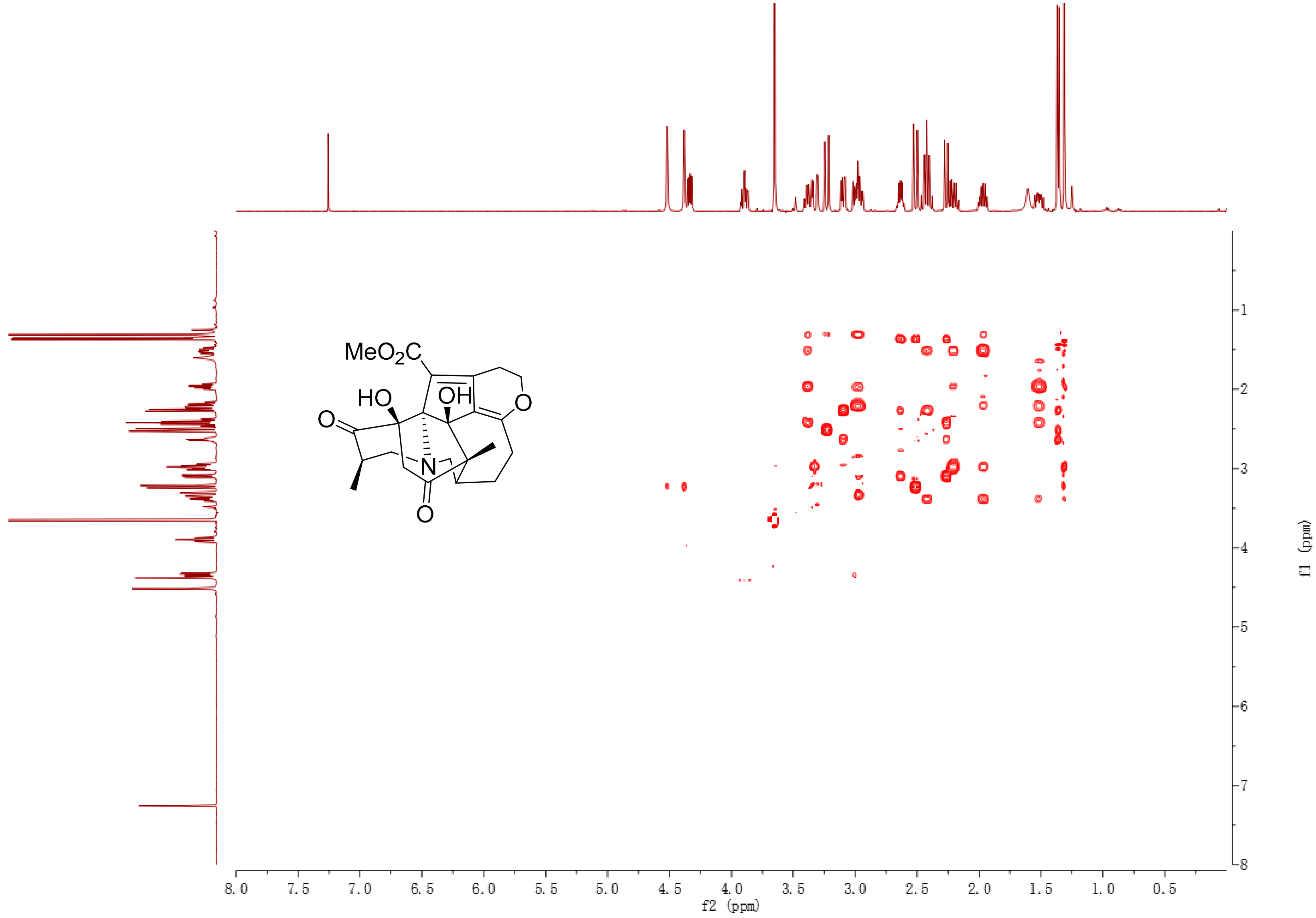

Figure S16 HR-ESI-MS spectrum of compound **2.**

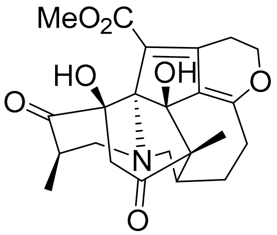

User Spectra

Fragmentor Voltage	Collision Energy	Ionization Mode
135	0	ESI

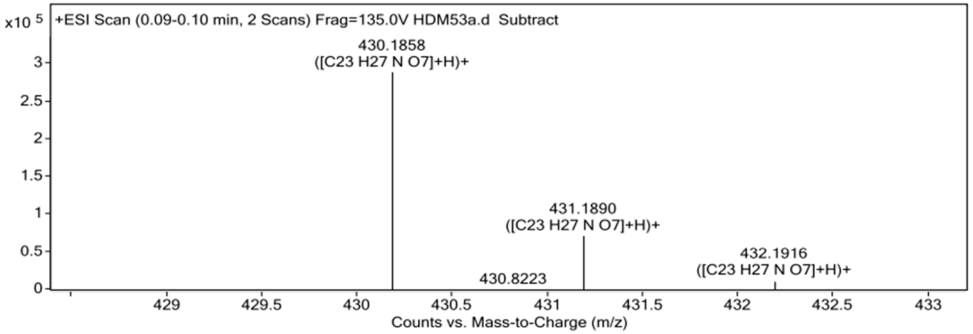

Peak List

m/z	z	Abund	Formula	Ion
79.0213	1	1814.01		
107.0315	1	1354.28		
122.0804	1	2044.06		
138.055	1	5218.59		
150.1118	1	6126.81		
430.1858	1	288726.5	C23 H27 N O7	(M+H)+
431.189	1	72031.63	C23 H27 N O7	(M+H)+
432.1916	1	12341.08	C23 H27 N O7	(M+H)+
433.1945	1	1208.6	C23 H27 N O7	(M+H)+
458.2161	1	1732.85		

Formula Calculator Element Limits

Element	Min	Max
C	3	60
H	0	200
O	0	30
N	0	5

Formula Calculator Results

Formula	CalculatedMass	CalculatedMz	Mz	Diff. (mDa)	Diff. (ppm)	DBE
C23 H27 N O7	429.1788	430.1860	430.1858	0.20	0.46	11.0000

Figure S17 IR (KBr disk) spectrum of compound **2**.

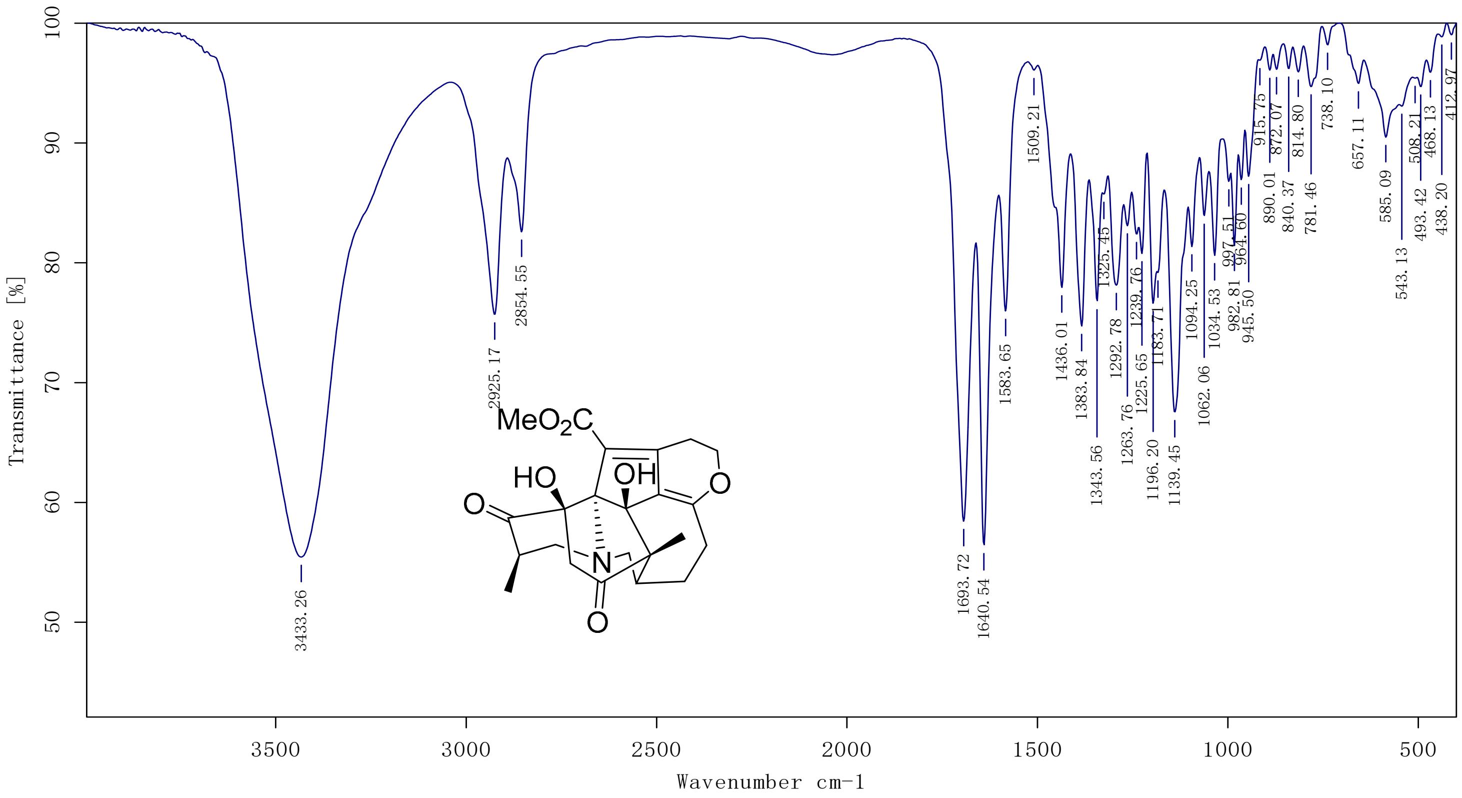

Sample Name: HDM53a
Sample Form: KBr
Path of File: E:\data
Date of Measurement: 2023/2/23

Resolution: 4
Aperture Setting: 6 mm
Number of Background Scans: 16
Number of Sample Scans: 16

Beamsplitter Setting: KBr
Source Setting: MIR
Instrument Type: BRUKER VERTEX 70
Soft Version: OPUS8.1

Figure S18 UV spectrum of compound **2.**

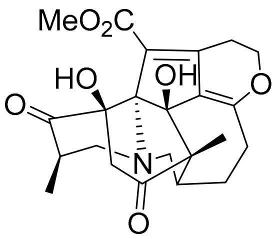

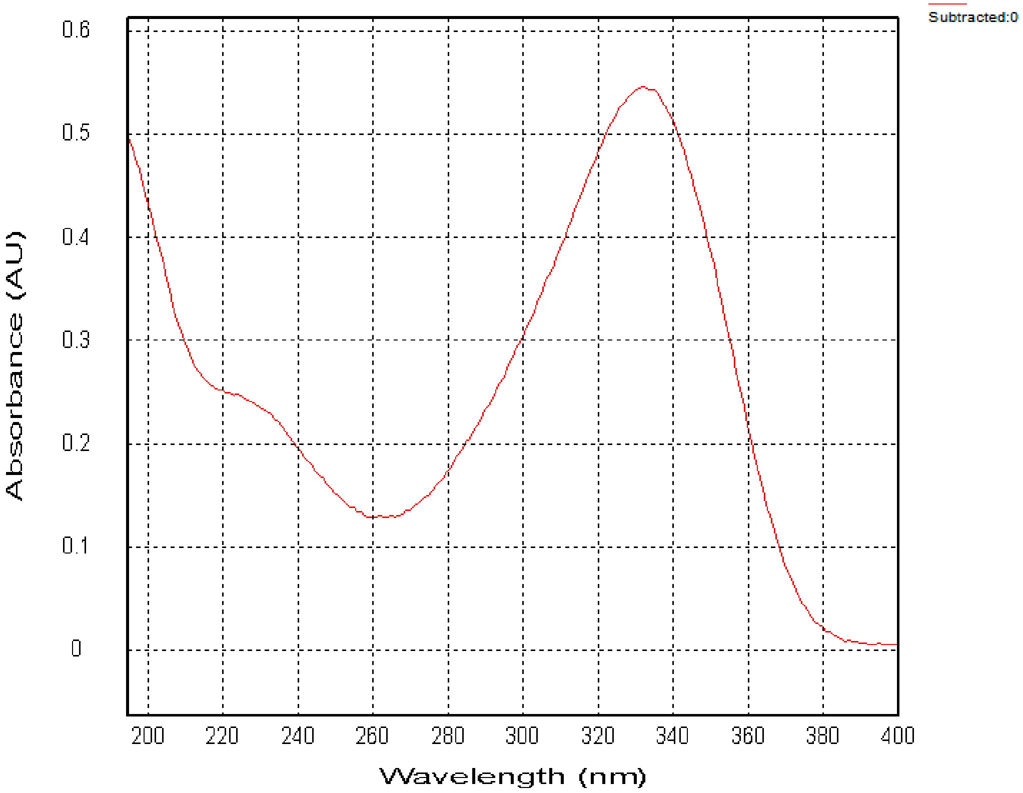

Figure S19 ^{1}H NMR (500MHz) spectrum of compound **3** in C_5D_5N

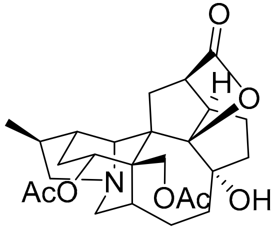

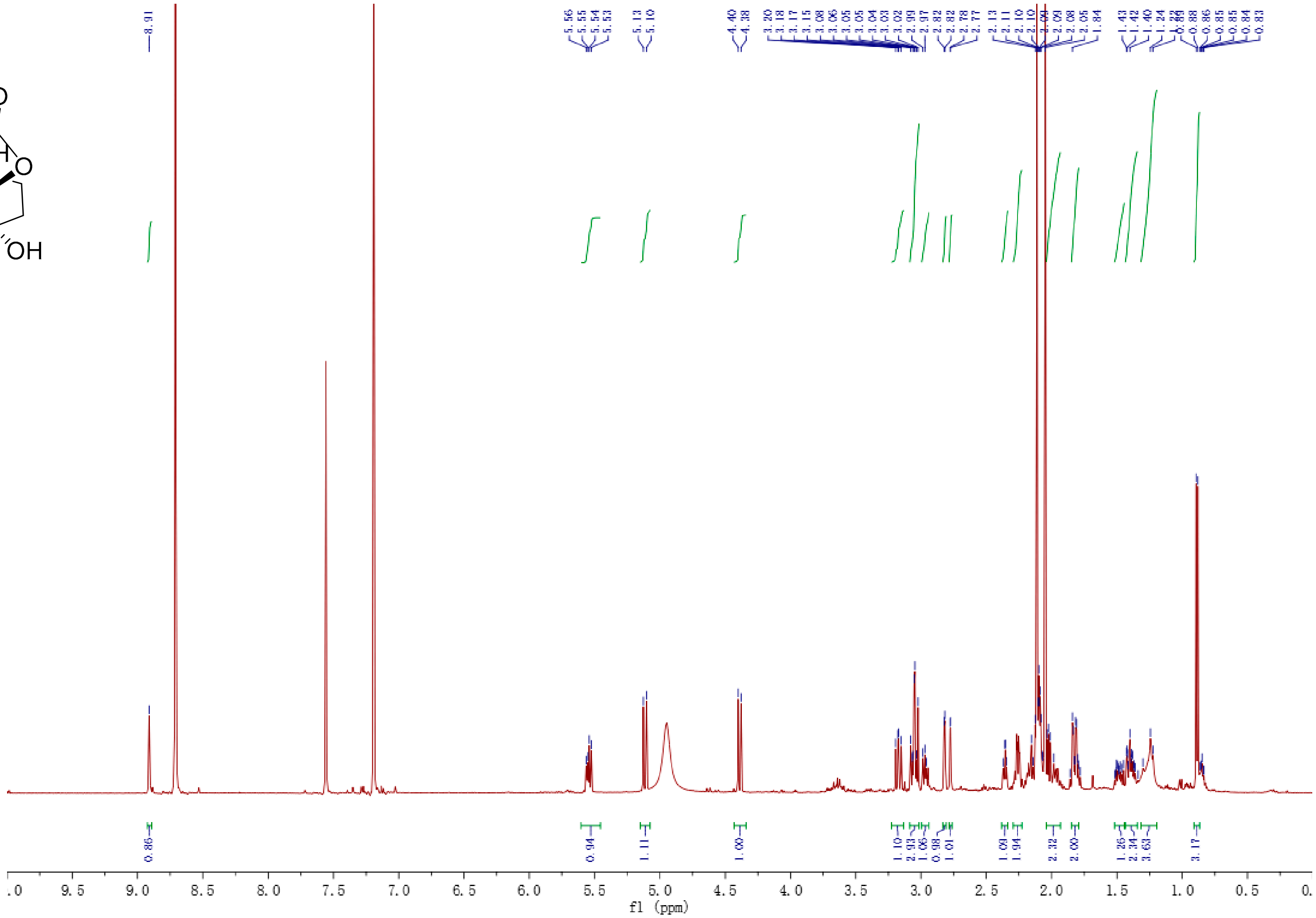

Figure S20 ^{13}C NMR (125 MHz) spectrum of compound **3** in C_5D_5N

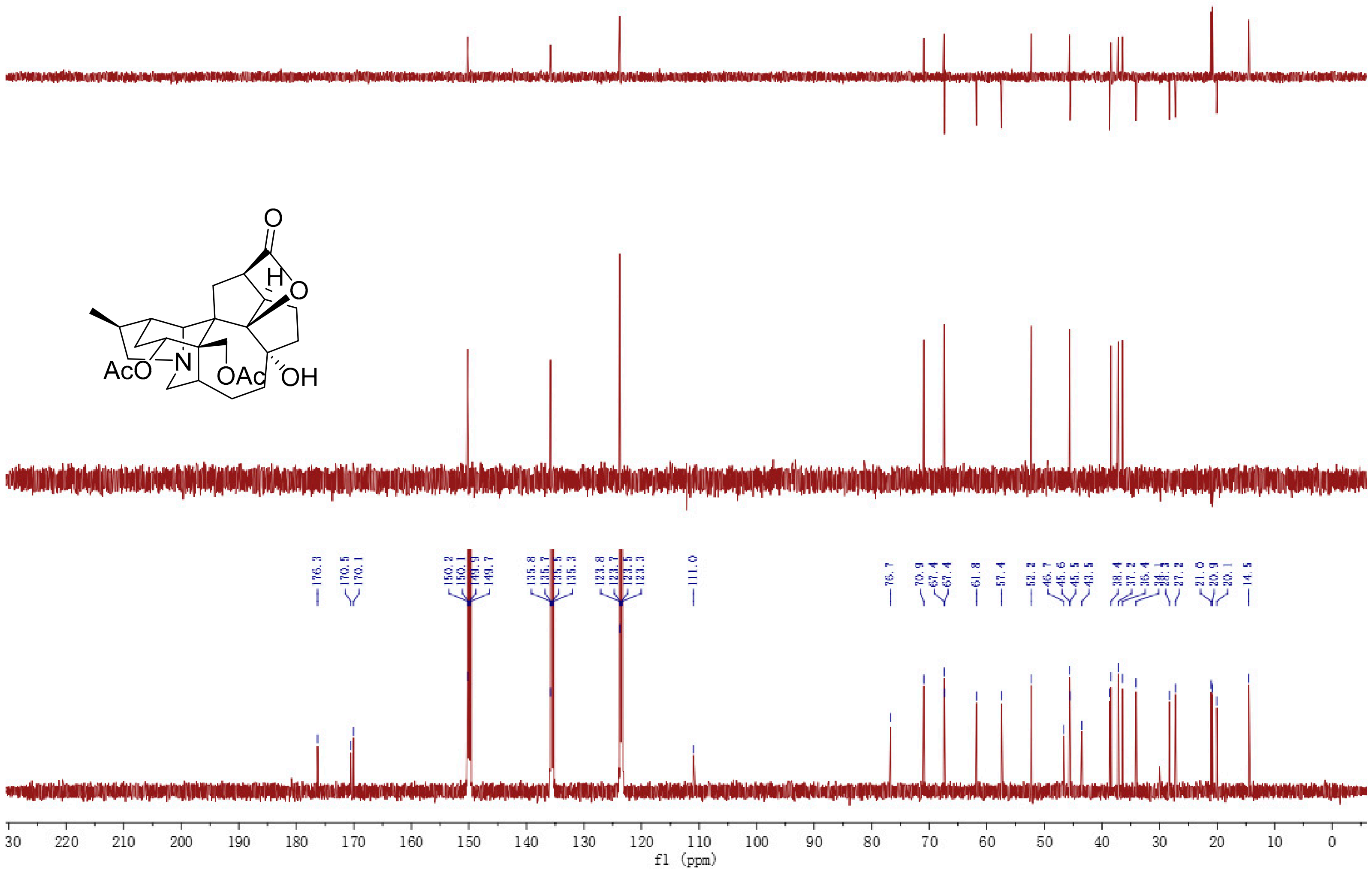

Figure S21 HSQC (500 MHz) spectrum of compound **3** in C_5D_5N

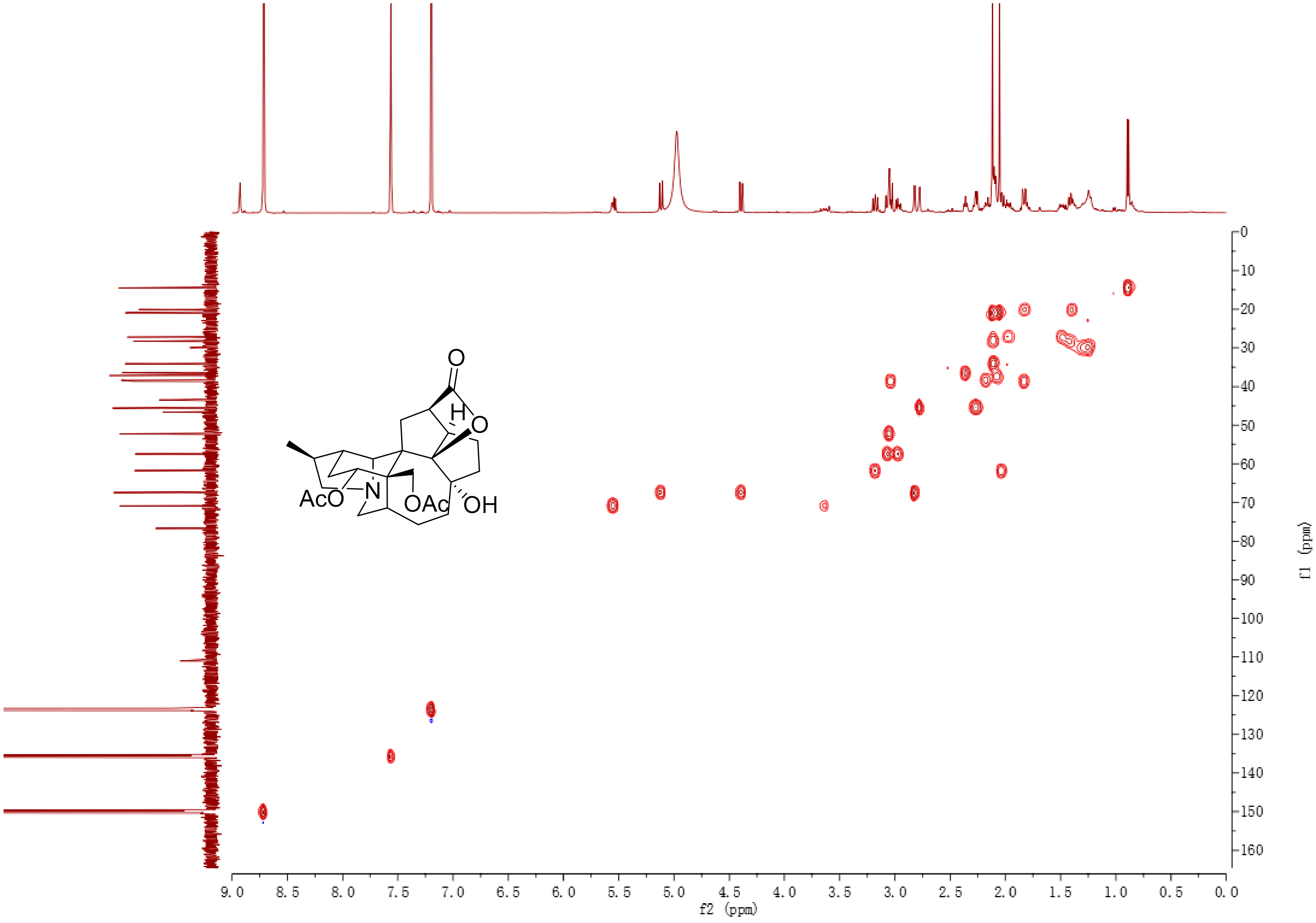

Figure S22 ^{1}H-^{1}H COSY (500 MHz) spectrum of compound **3** in C_5D_5N

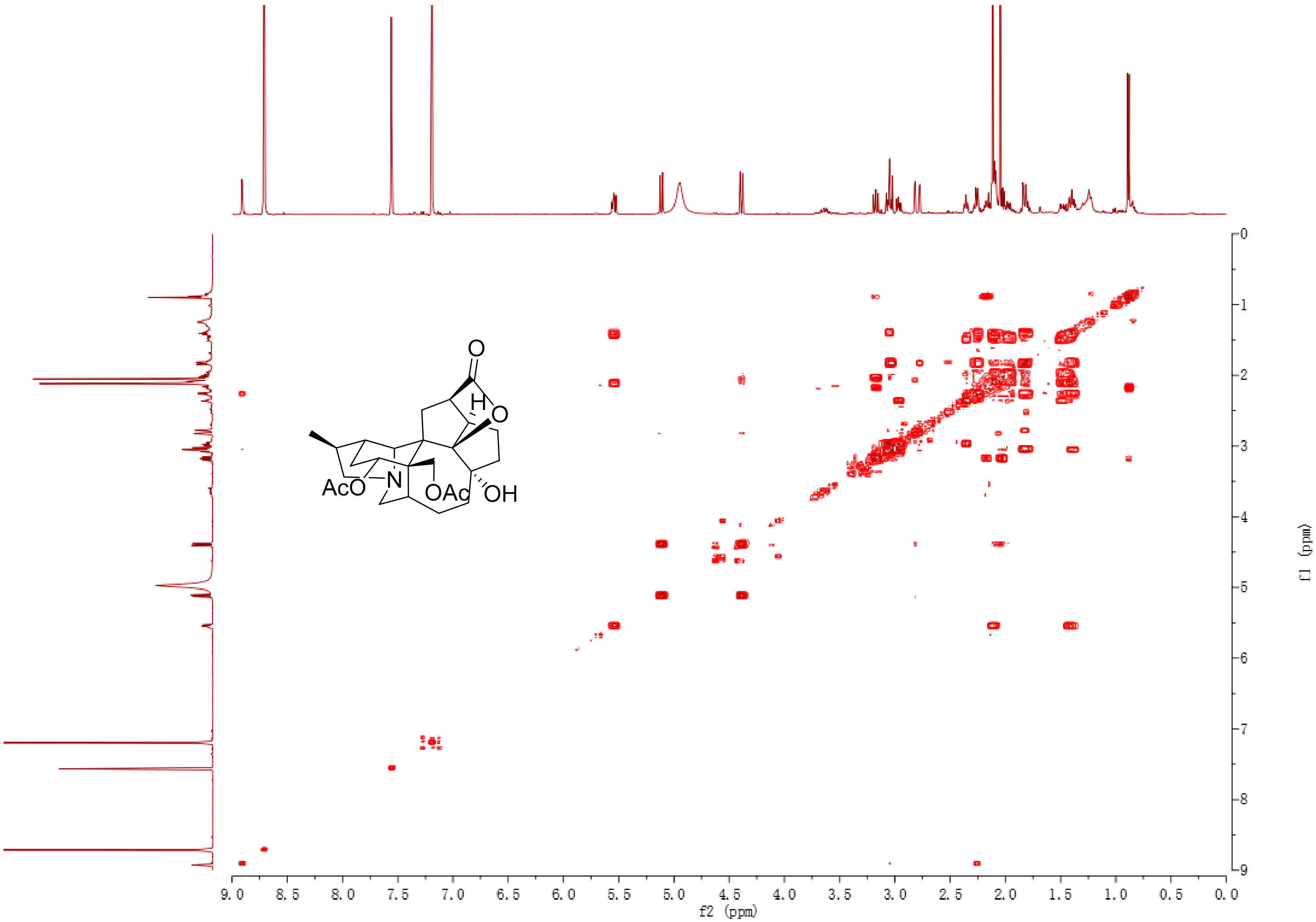

Figure S23 HMBC (500 MHz) spectrum of compound **3** in C_5D_5N

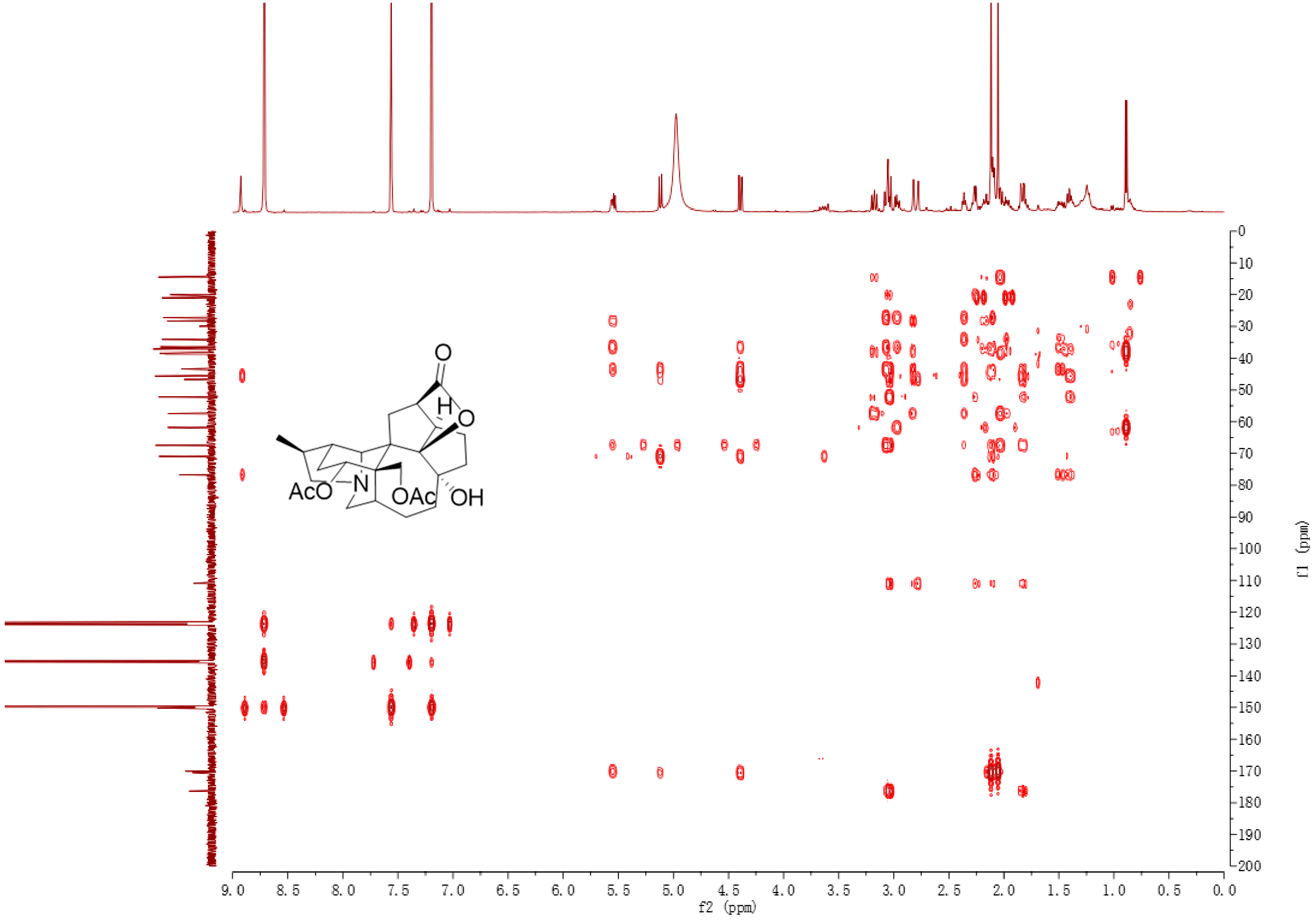

Figure S24 ROESY (500 MHz) spectrum of compound **3** in C_5D_5N

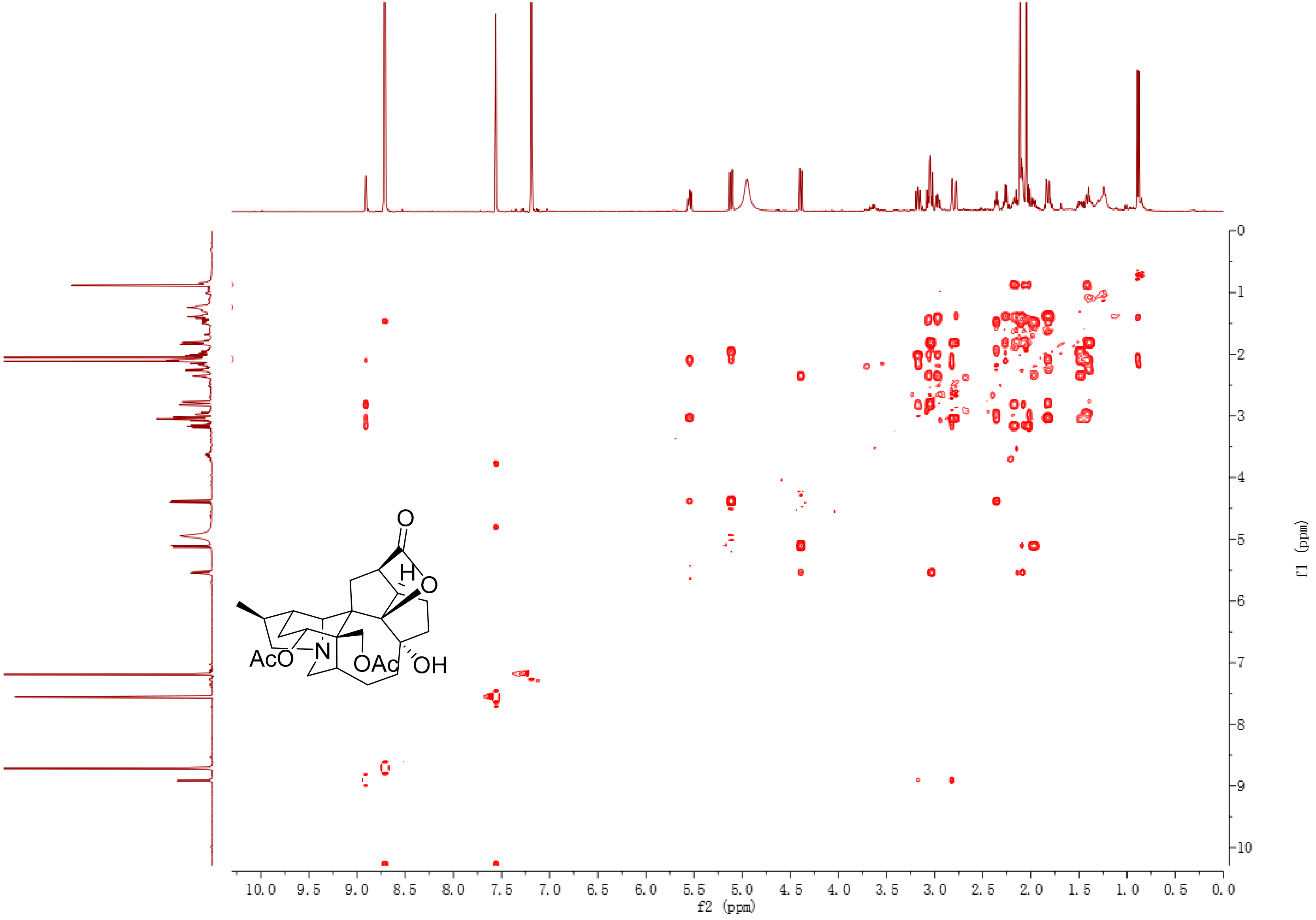

Figure S25 HR-ESI-MS spectrum of compound **3**

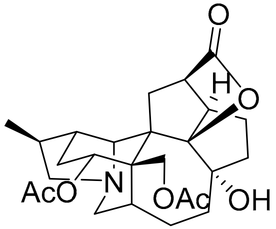

User Spectra

Fragmentor Voltage	Collision Energy	Ionization Mode
135	0	ESI

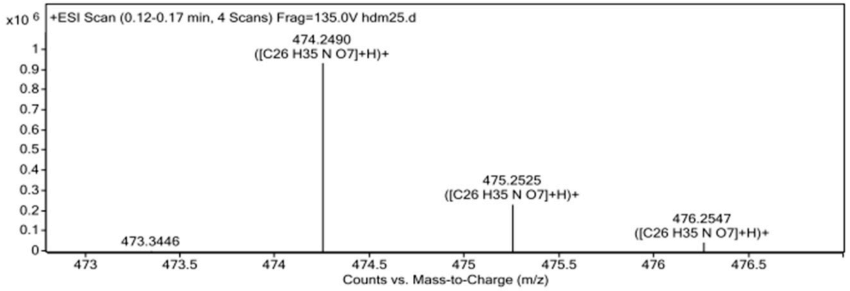

Peak List

m/z	z	Abund	Formula	Ion
79.0213	1	80878.47		
121.0509	1	80576.34		
280.1909	1	59593.27		
460.233	1	27808.28		
468.3905	1	25105.37		
470.4102	1	32461.55		
474.249	1	934520.13	C26 H35 N O7	(M+H)+
475.2525	1	236321.7	C26 H35 N O7	(M+H)+
476.2547	1	46313.09	C26 H35 N O7	(M+H)+
922.0098	1	95659.21		

Formula Calculator Element Limits

Element	Min	Max
C	3	60
H	0	120
O	0	30
N	0	5

Formula Calculator Results

Formula	CalculatedMass	CalculatedMz	Mz	Diff. (mDa)	Diff. (ppm)	DBE
C26 H35 N O7	473.2414	474.2486	474.2490	-0.40	-0.84	10.0000

Figure S26 IR (KBr disk) spectrum of compound **3**.

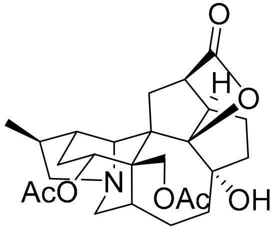

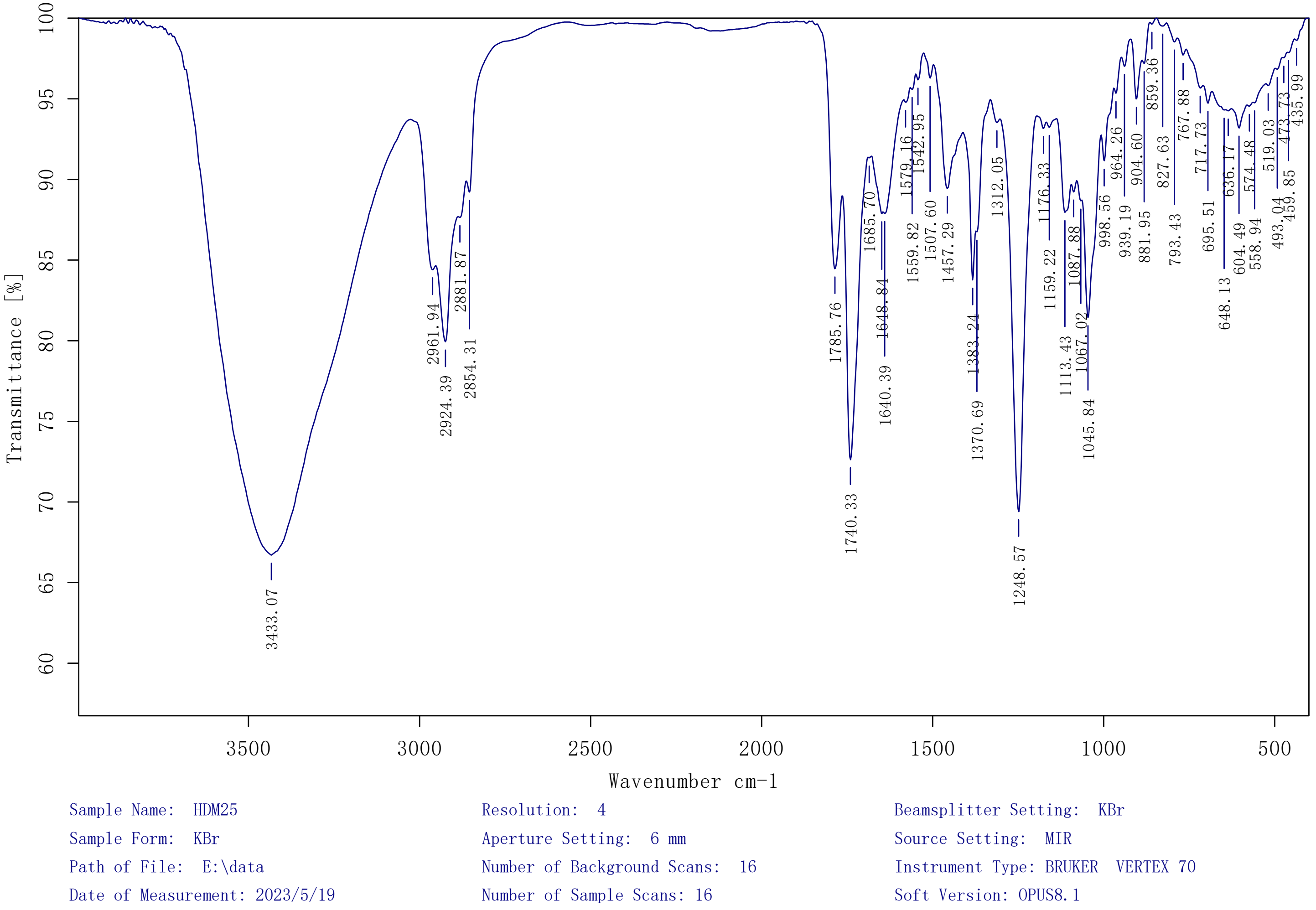

Figure S27 UV spectrum of compound **3**

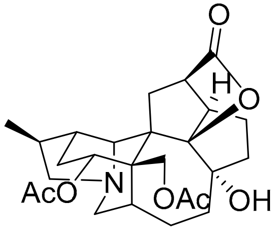

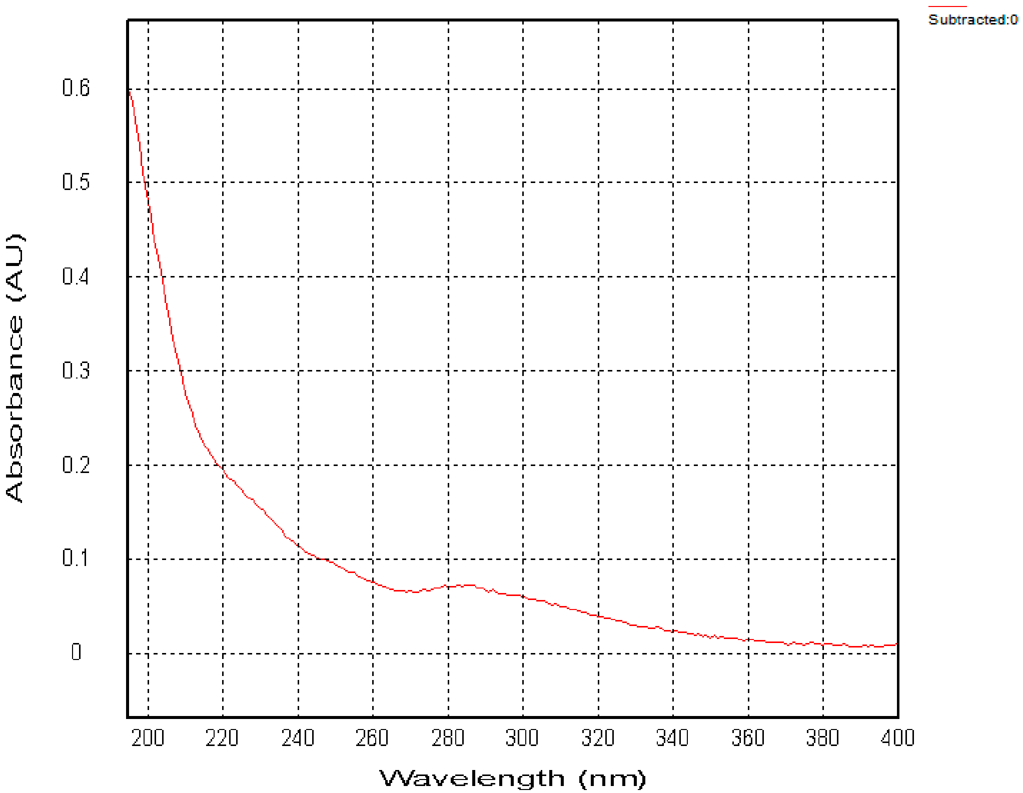

Figure S28 ECD spectrum of compound **3**

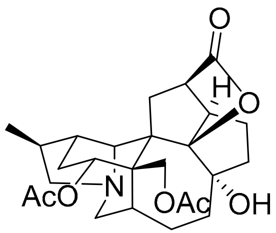

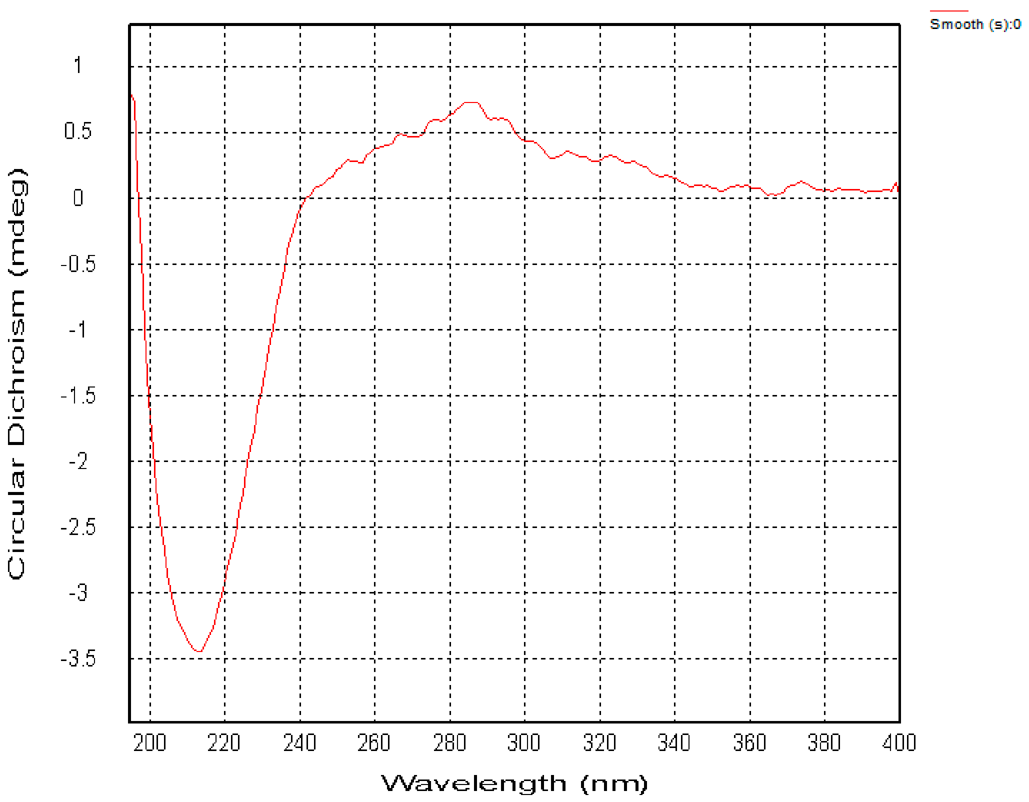

Figure S29 ^{1}H NMR (500MHz) spectrum of compound **4** in C_5D_5N

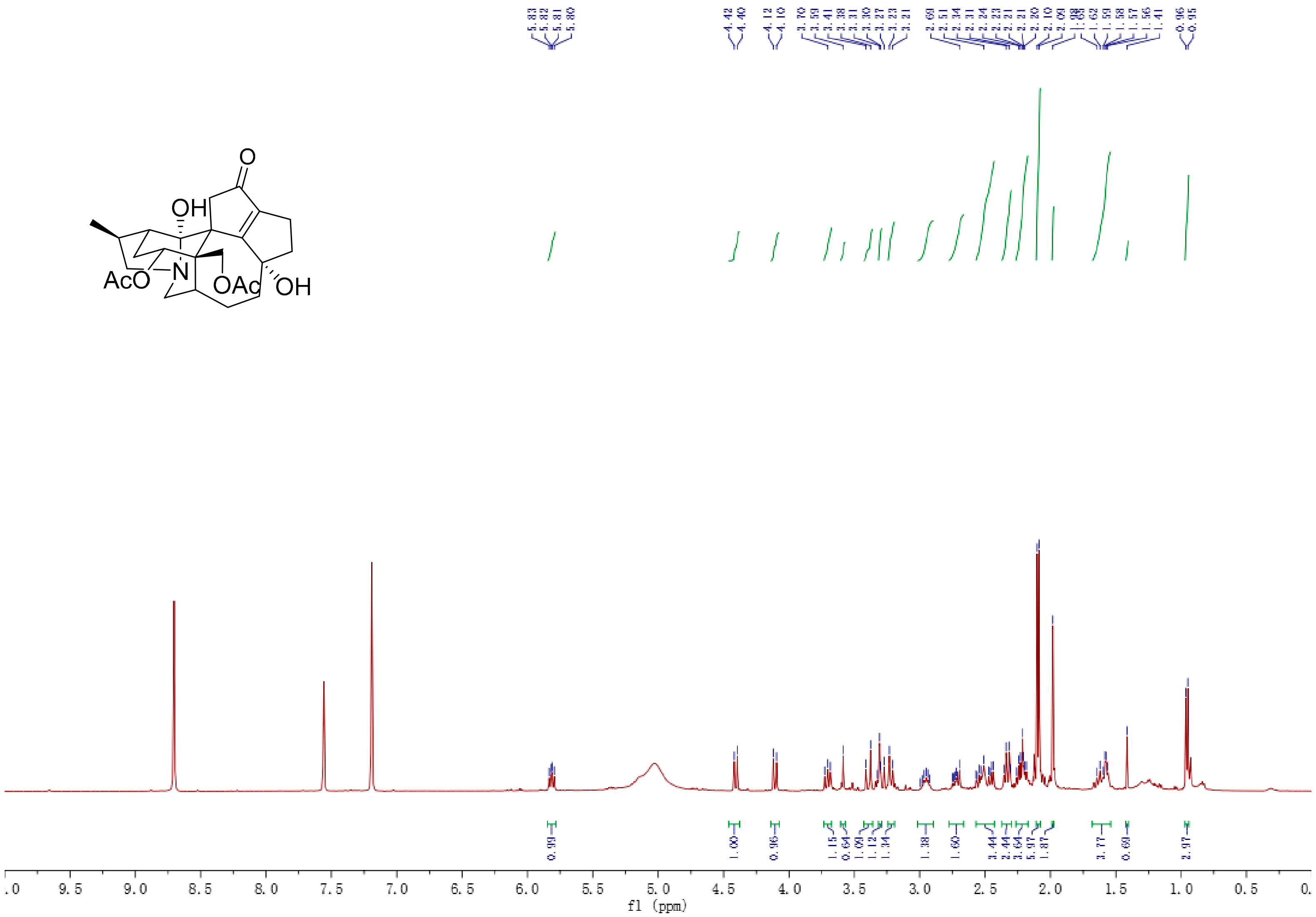

Figure S30 ^{13}C NMR (125 MHz) spectrum of compound **4** in C_5D_5N

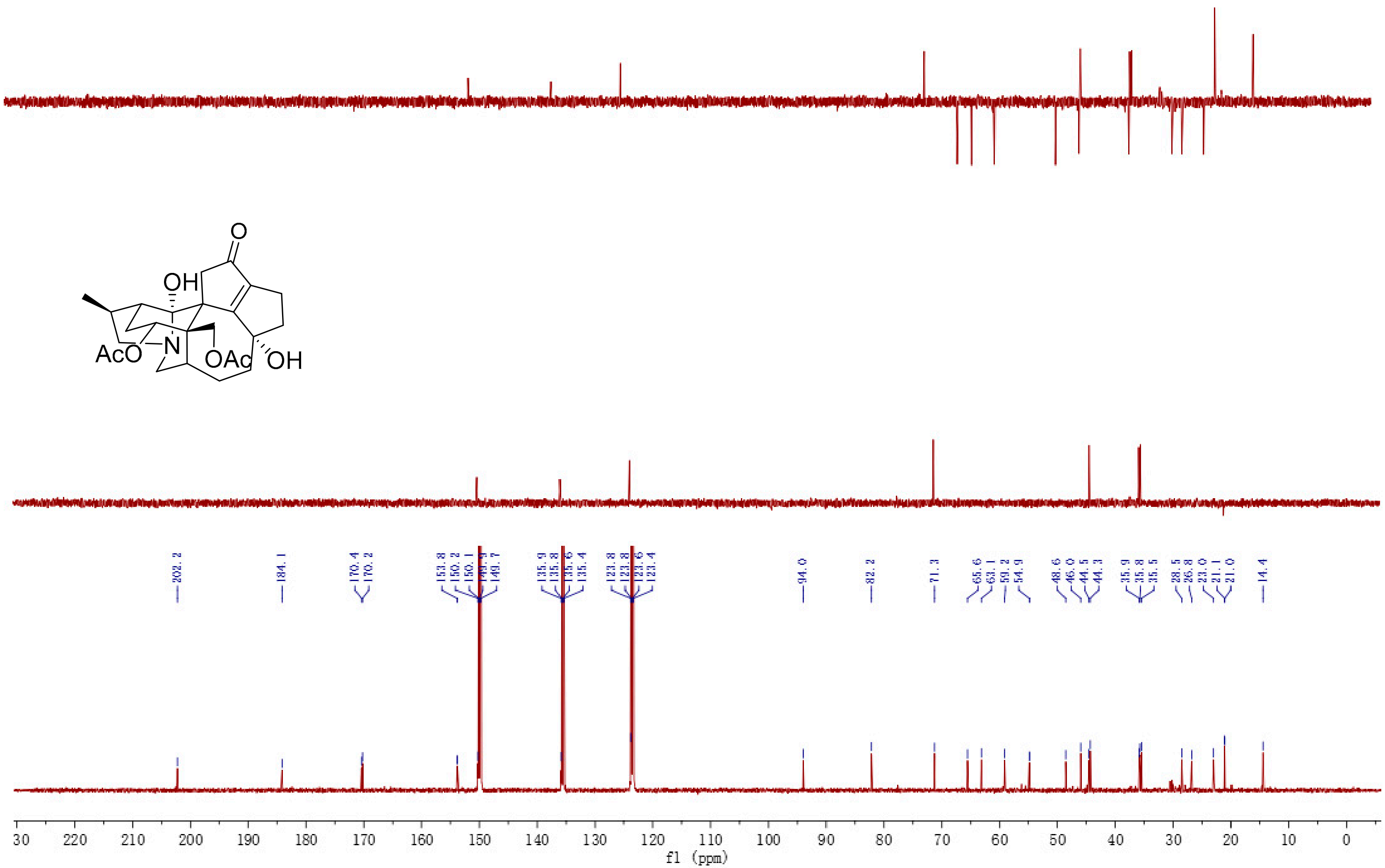

Figure S31 HSQC (500 MHz) spectrum of compound **4** in C_5D_5N

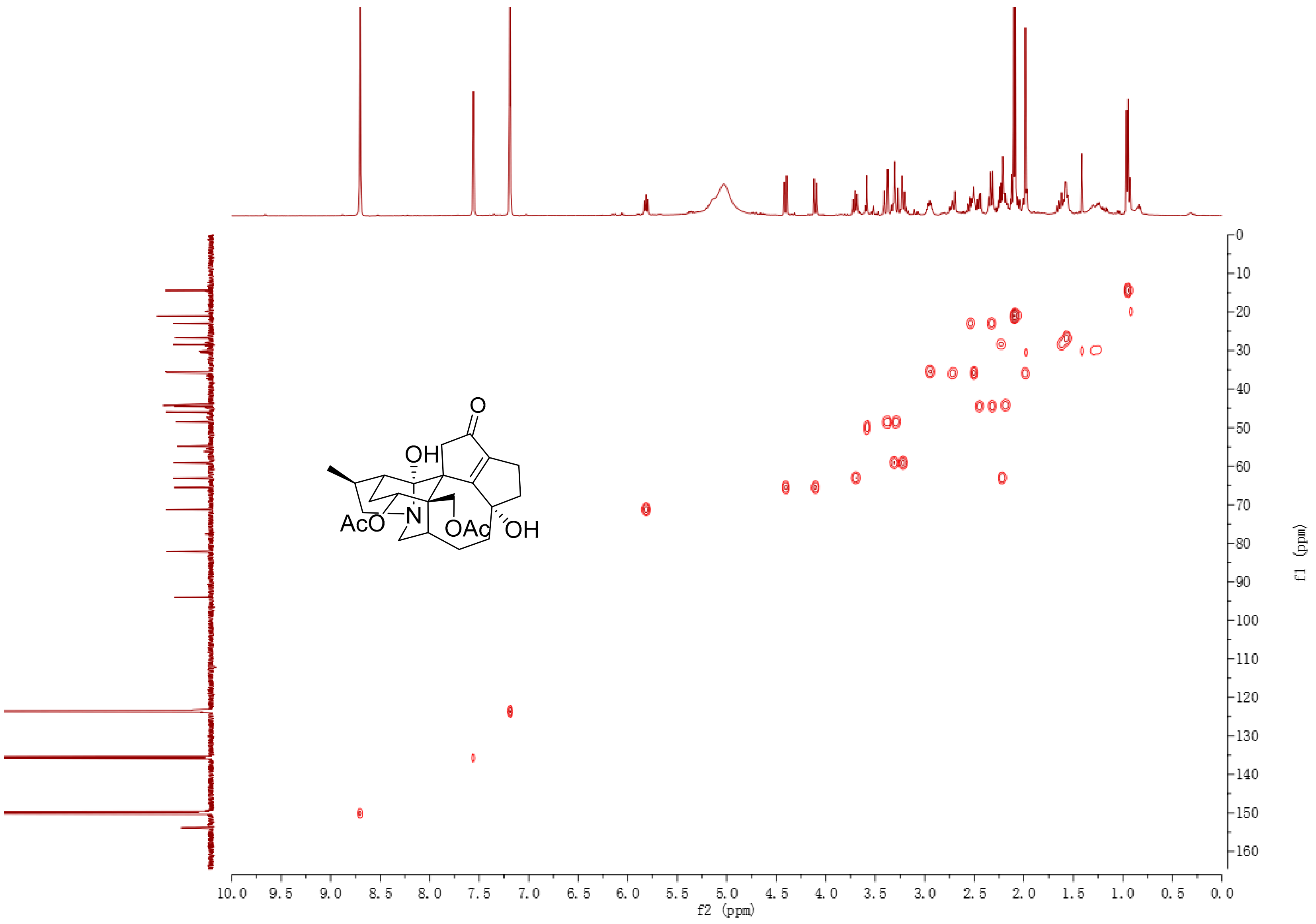

Figure S32 ^1H-^1H COSY (500 MHz) spectrum of compound **4** in C_5D_5N

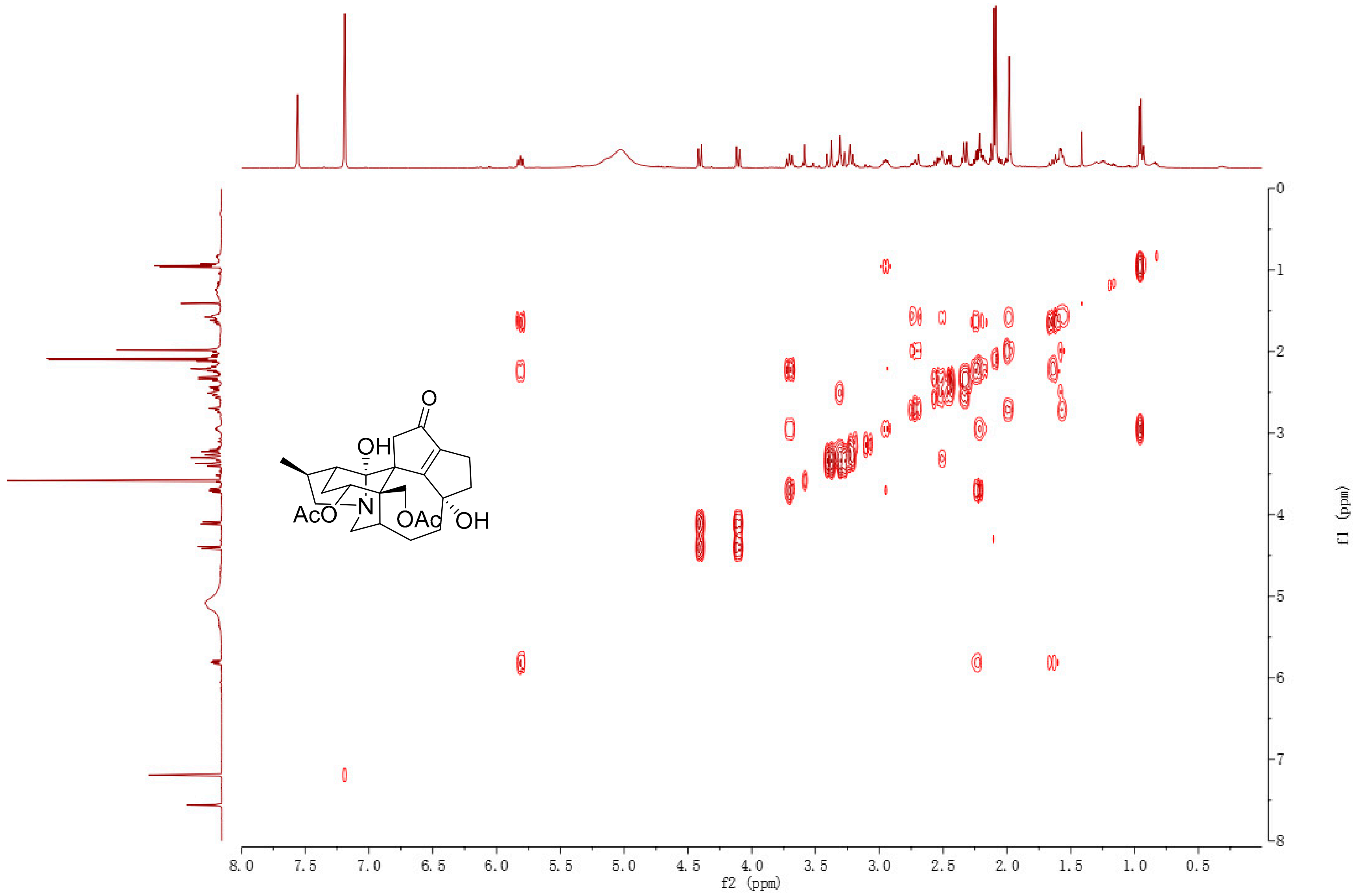

Figure S33 HMBC (500 MHz) spectrum of compound **4** in C_5D_5N

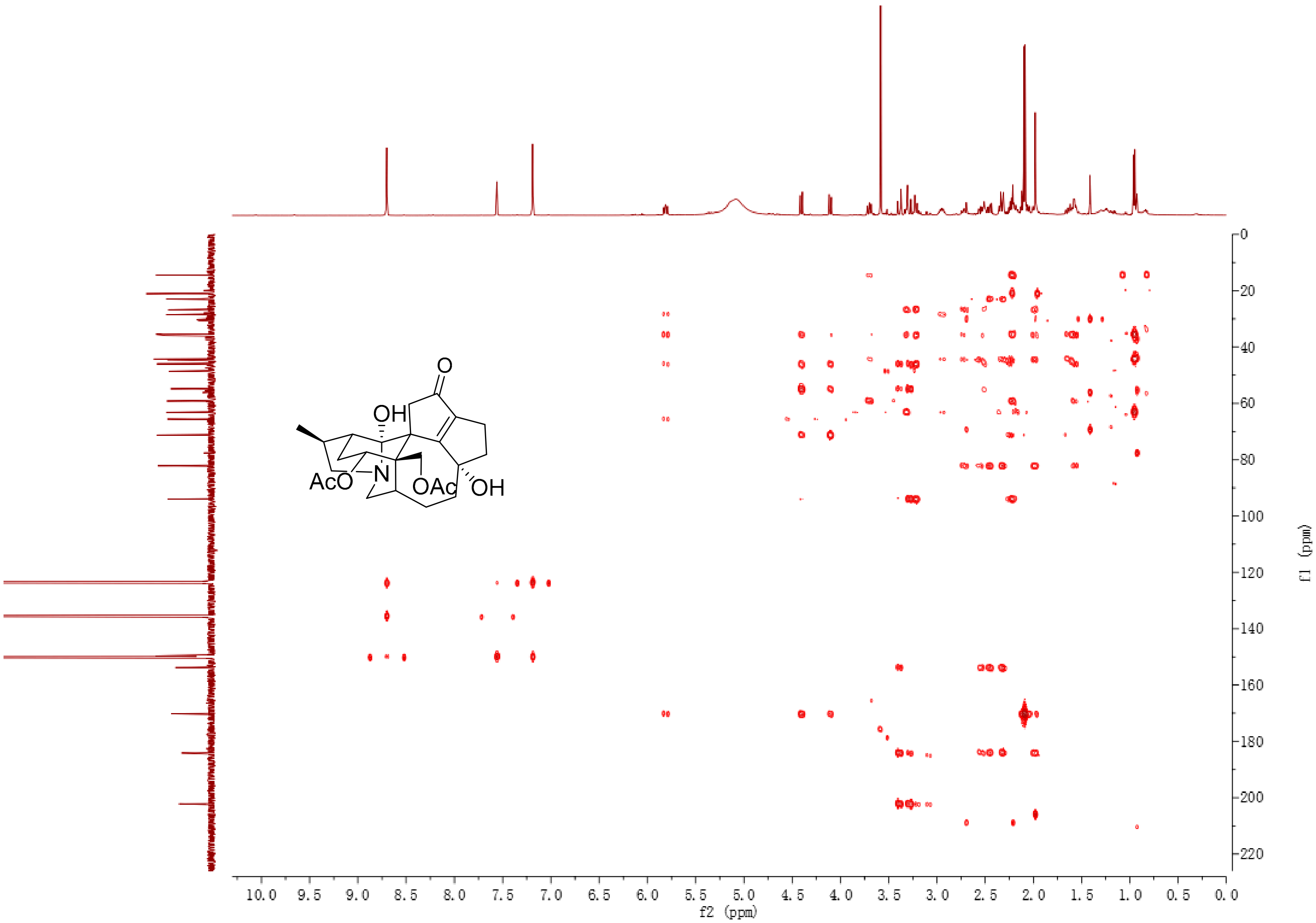

Figure S34 ROESY (500 MHz) spectrum of compound **4** in C_5D_5N

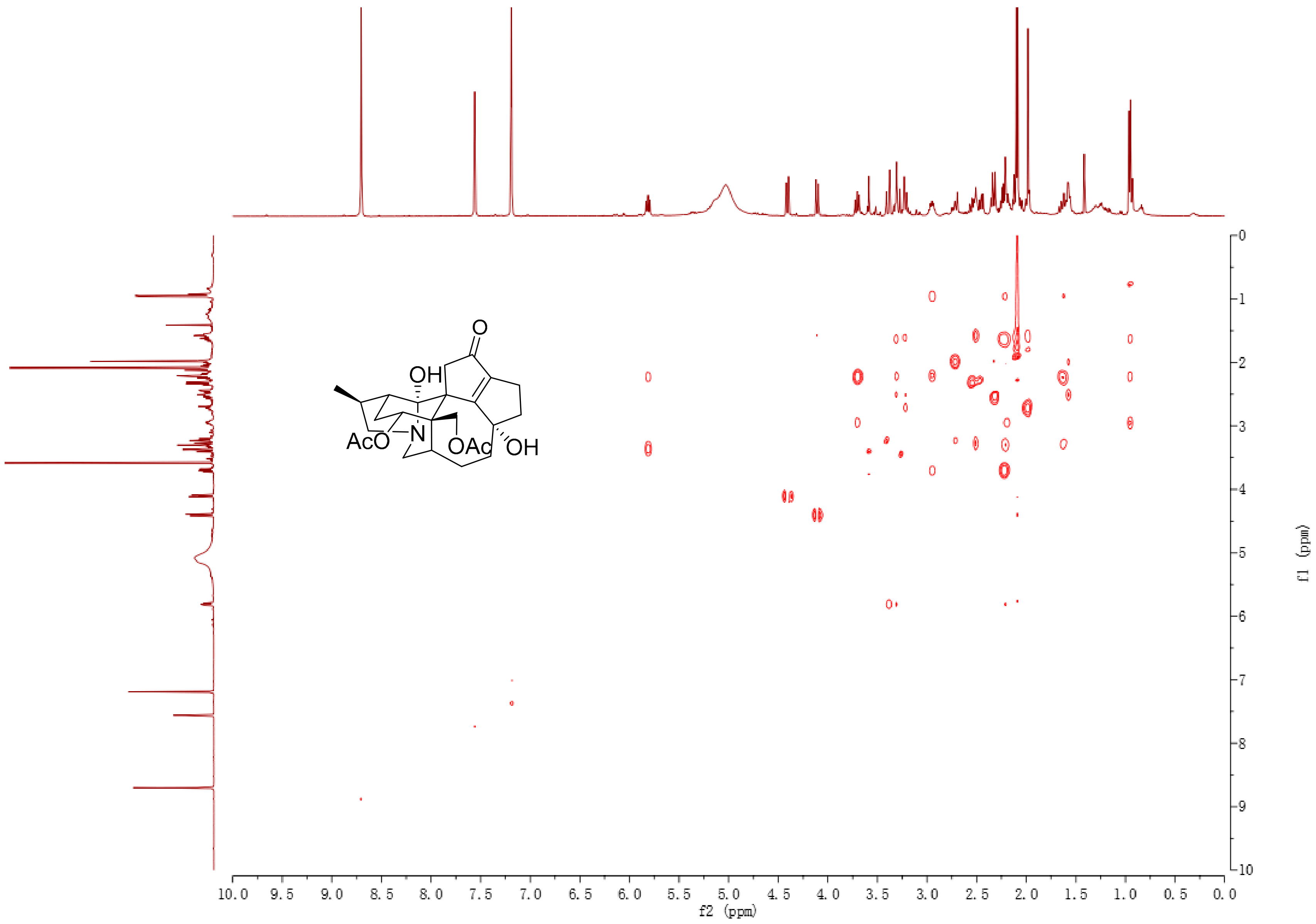

Figure S35 HR-ESI-MS spectrum of compound **4**

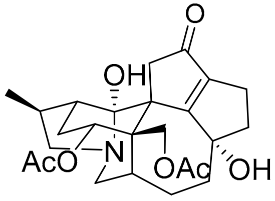

User Spectra

Fragmentor Voltage	Collision Energy	Ionization Mode
135	0	ESI

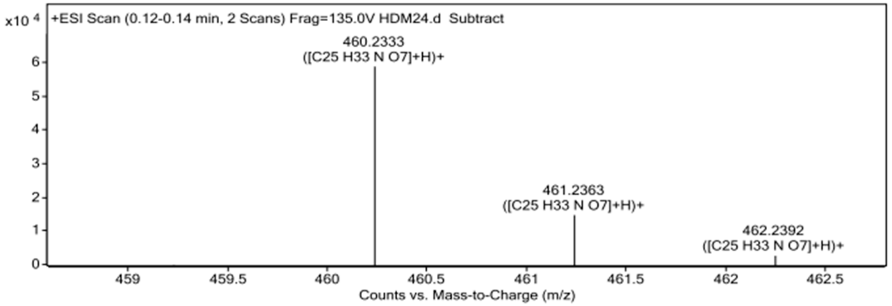

Peak List

m/z	z	Abund	Formula	Ion
85.0808	1	11835.3		
402.2271	1	13688.43		
403.2331	1	2674.7		
442.2214	1	3507.74		
460.2333	1	58990.14	C25 H33 N O7	(M+H)+
461.2363	1	15019.16	C25 H33 N O7	(M+H)+
462.2392	1	2932.23	C25 H33 N O7	(M+H)+
482.215	1	4266.11		
486.2488	1	3418.46		
941.4397	1	2903.23		

Formula Calculator Element Limits

Element	Min	Max
C	3	60
H	0	120
O	0	30
N	0	5

Formula Calculator Results

Formula	CalculatedMass	CalculatedMz	Mz	Diff. (mDa)	Diff. (ppm)	DBE
C25 H33 N O7	459.2257	460.2330	460.2333	-0.30	-0.65	10.0000

Figure S36 IR (KBr disk) spectrum of compound **4**

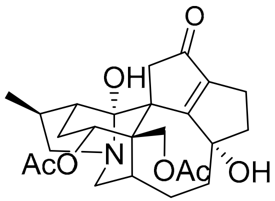

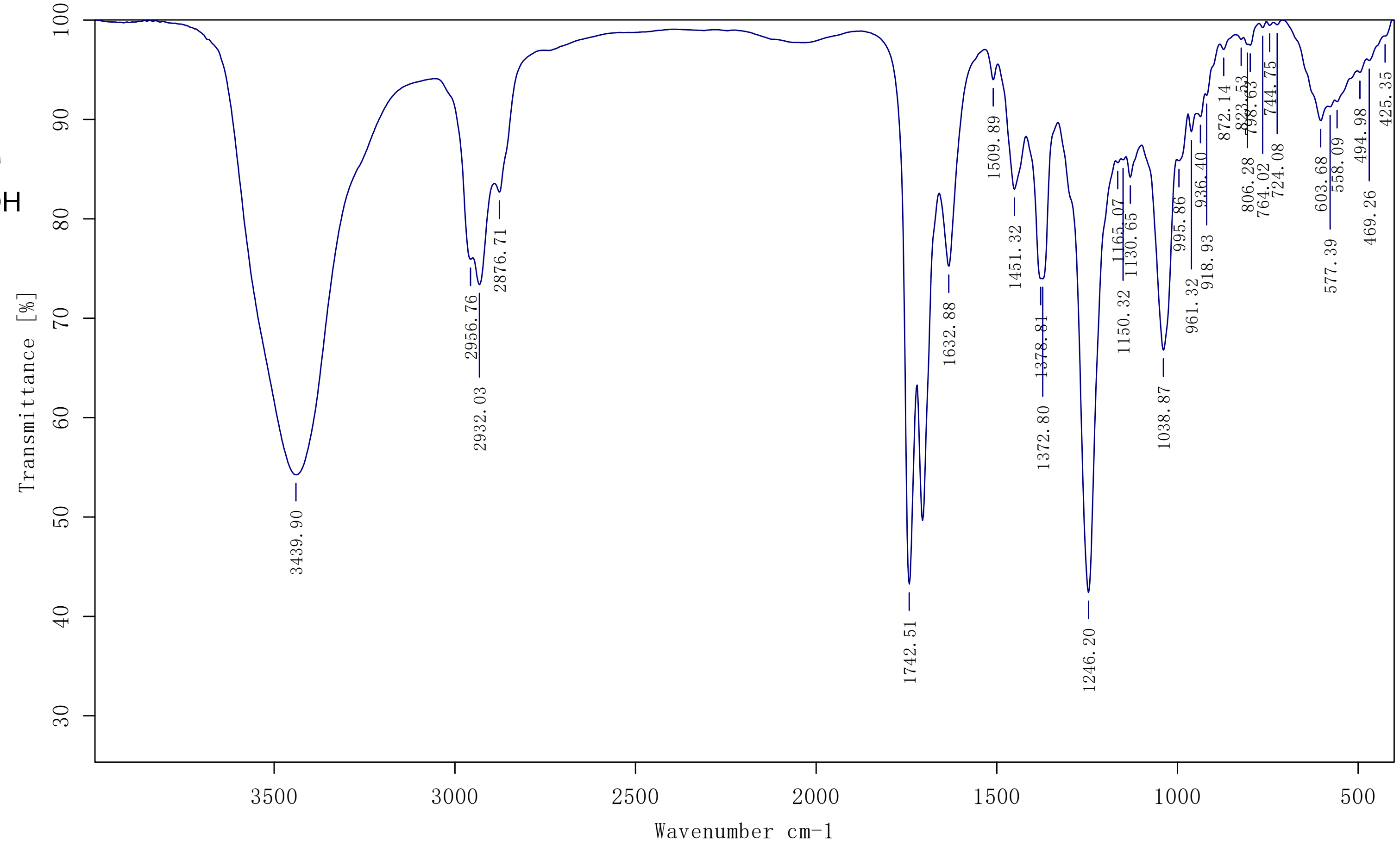

Sample Name: HDM24
Sample Form: KBr
Path of File: E:\data
Date of Measurement: 2023/5/19

Resolution: 4
Aperture Setting: 6 mm
Number of Background Scans: 16
Number of Sample Scans: 16

Beamsplitter Setting: KBr
Source Setting: MIR
Instrument Type: BRUKER VERTEX 70
Soft Version: OPUS8.1

Figure S37 UV spectrum of compound **4**

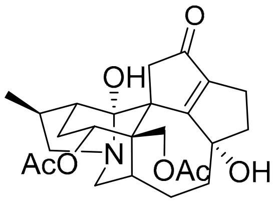

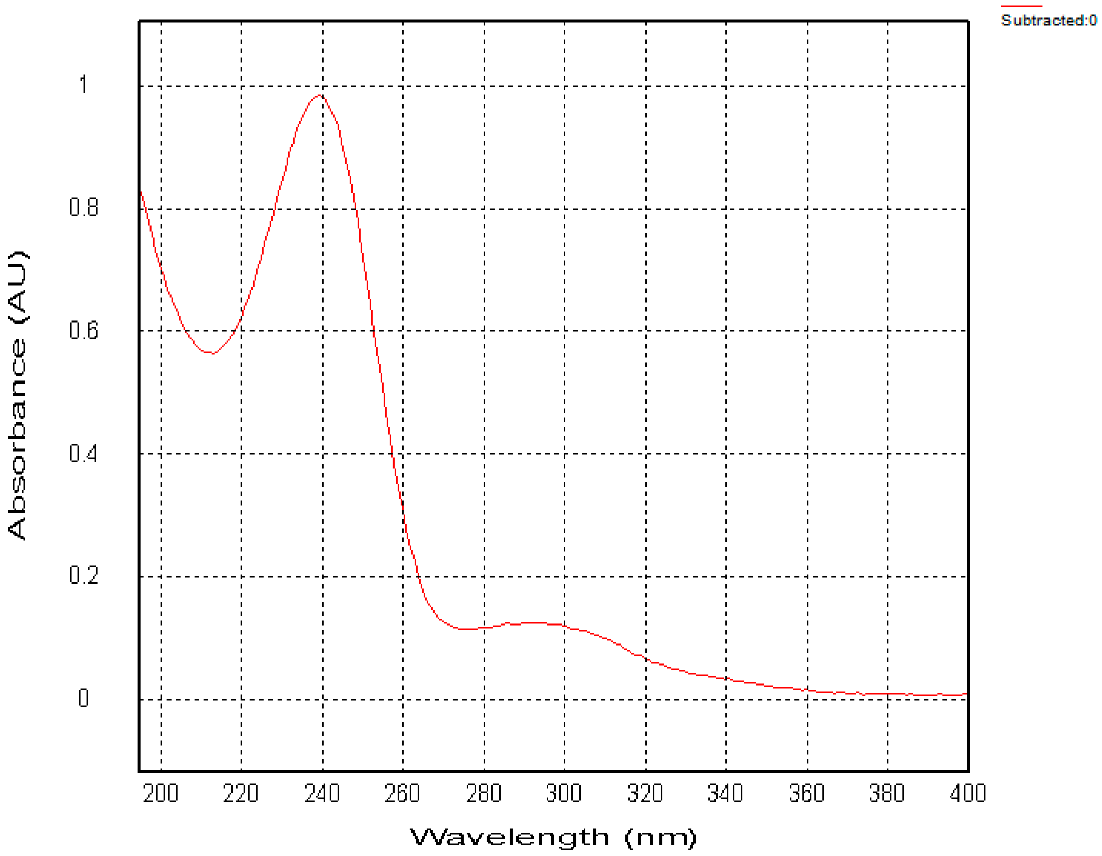

Figure S38 ECD spectrum of compound **4**

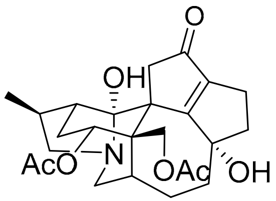

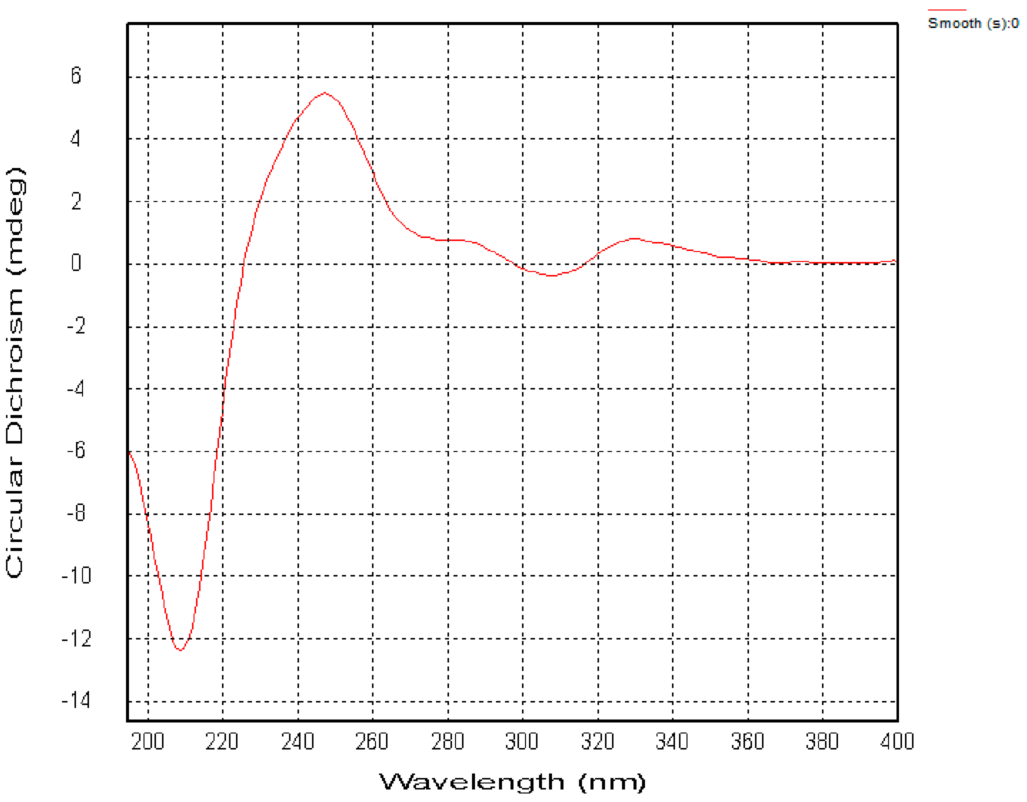

Figure S39 ^{1}H NMR (500MHz) spectrum of compound **5** in C_5D_5N

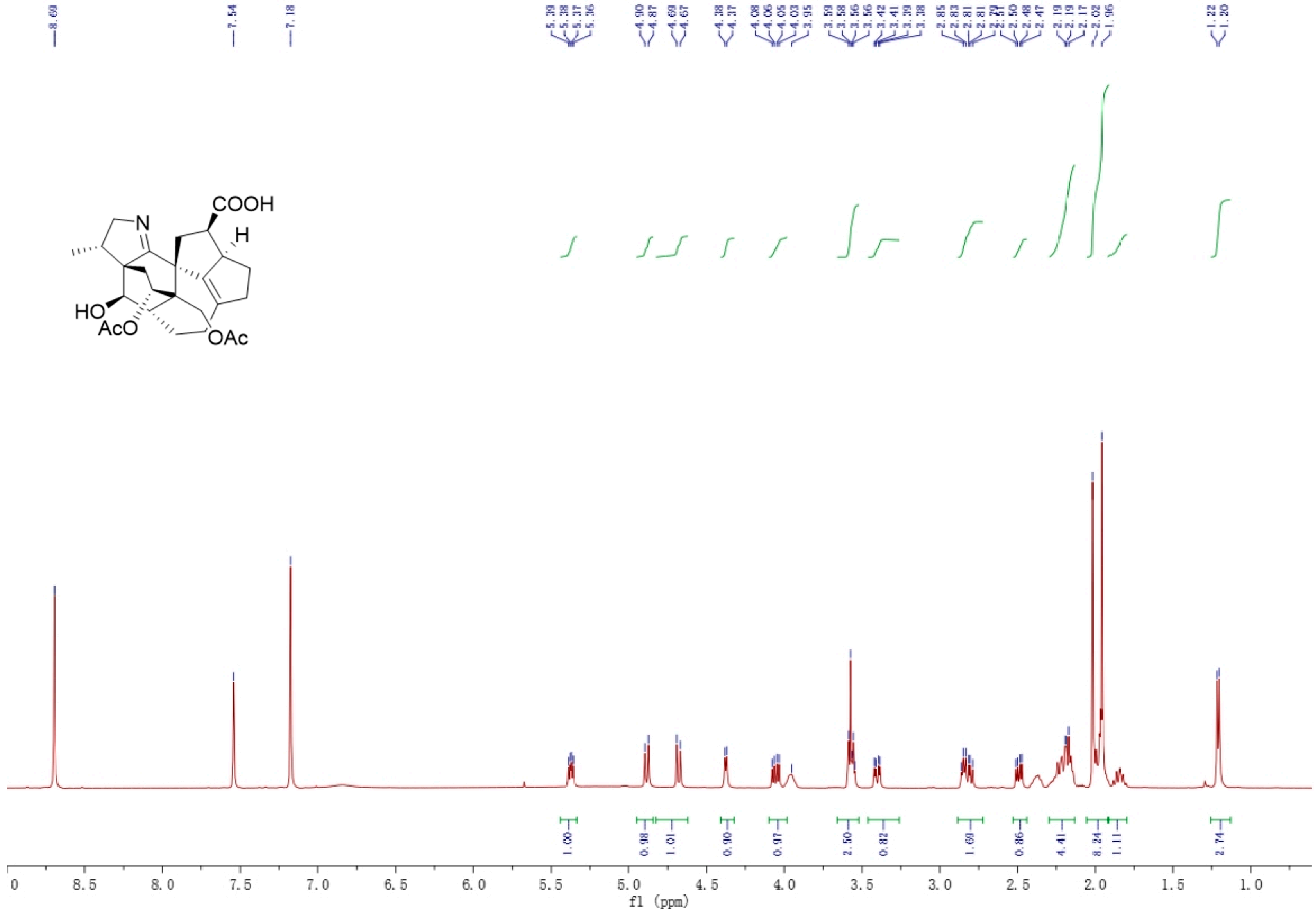

Figure S40 ^{13}C NMR (500MHz) spectrum of compound **5** in C_5D_5N

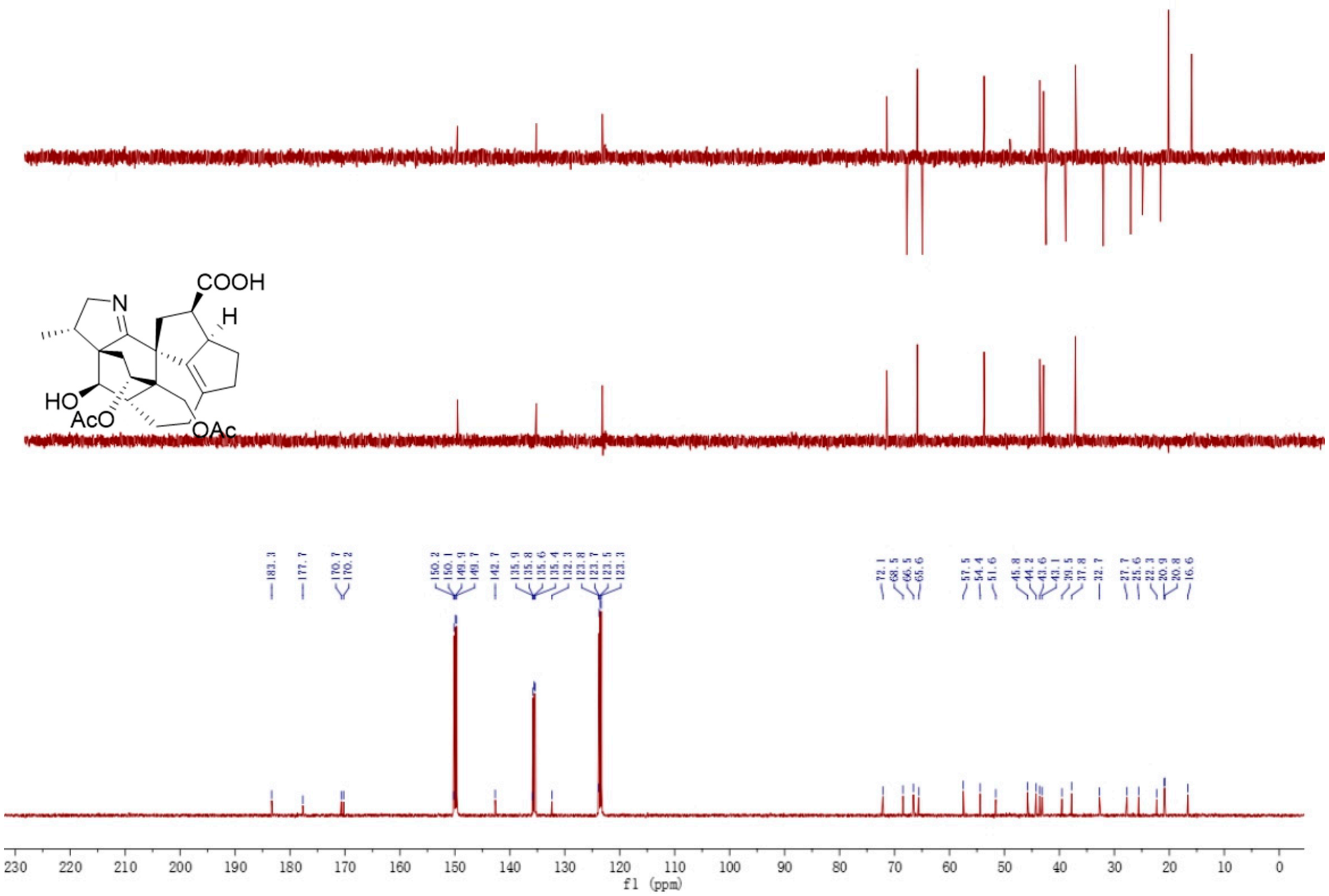

Figure S41 HSQC (500 MHz) spectrum of compound **5** in C_5D_5N

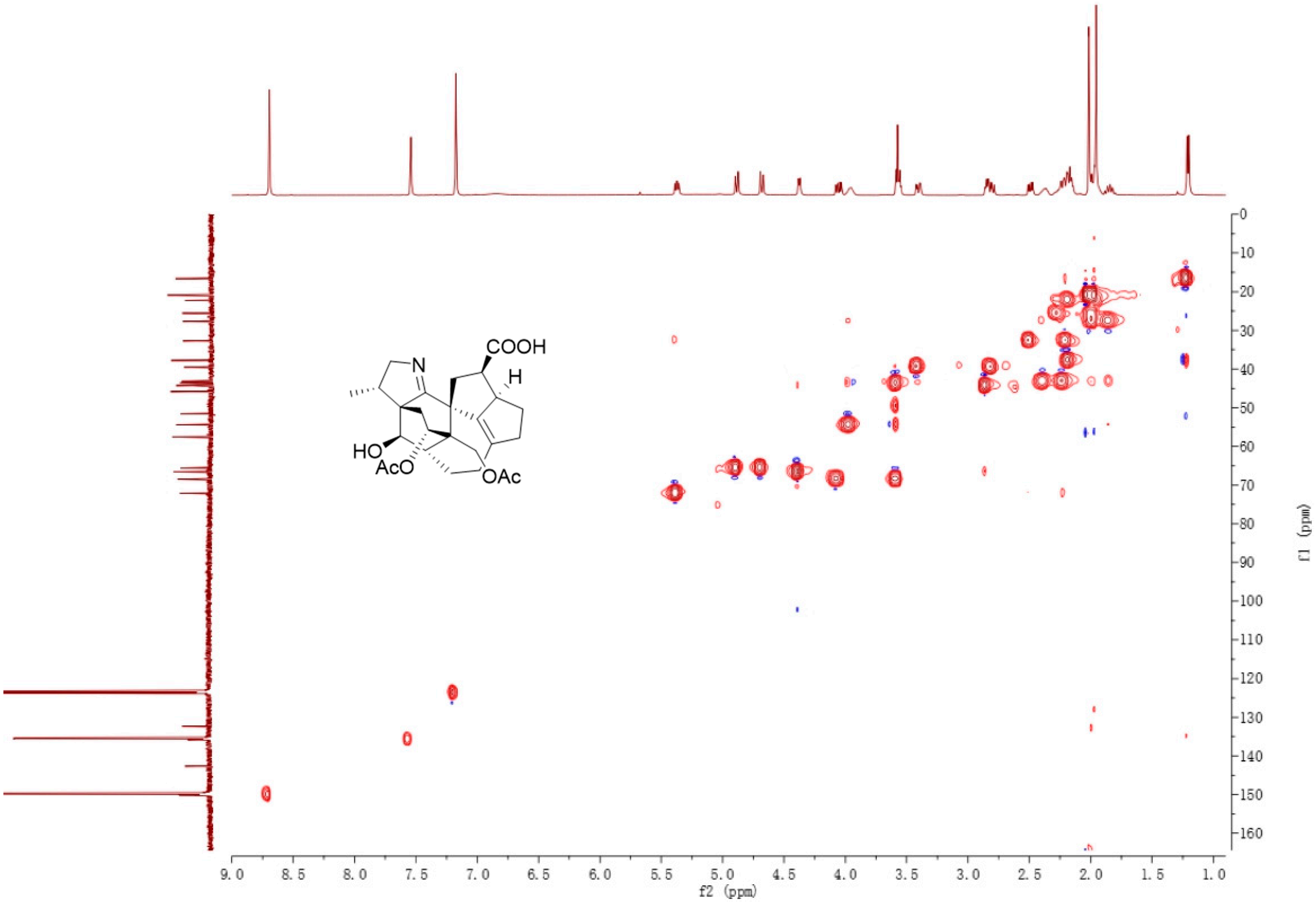

Figure S42 ^{1}H-^{1}H COSY (500 MHz) spectrum of compound **5** in C_5D_5N

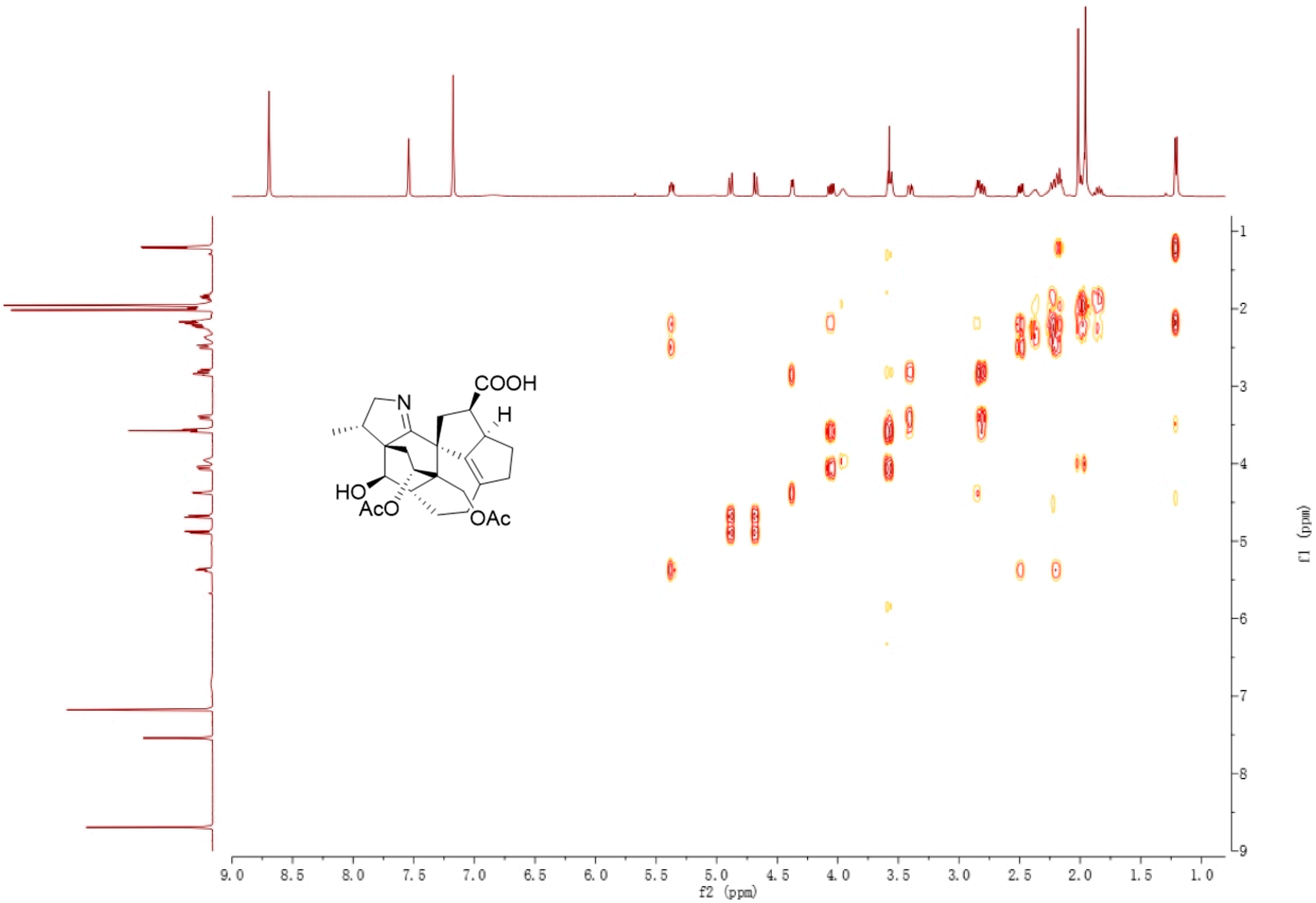

Figure S43 HMBC (500 MHz) spectrum of compound **5** in C_5D_5N

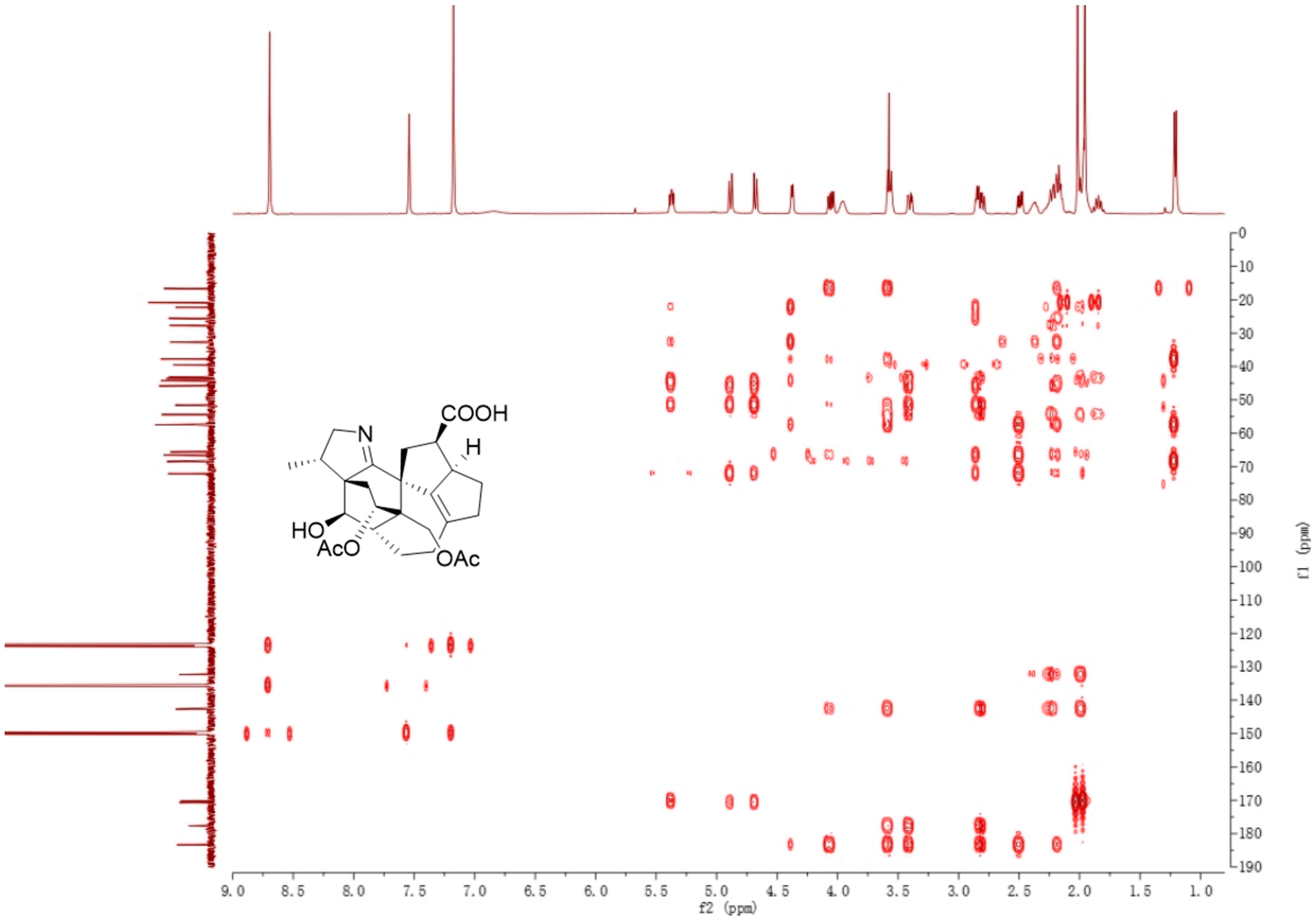

Figure S44 ROESY (500 MHz) spectrum of compound **5** in C_5D_5N

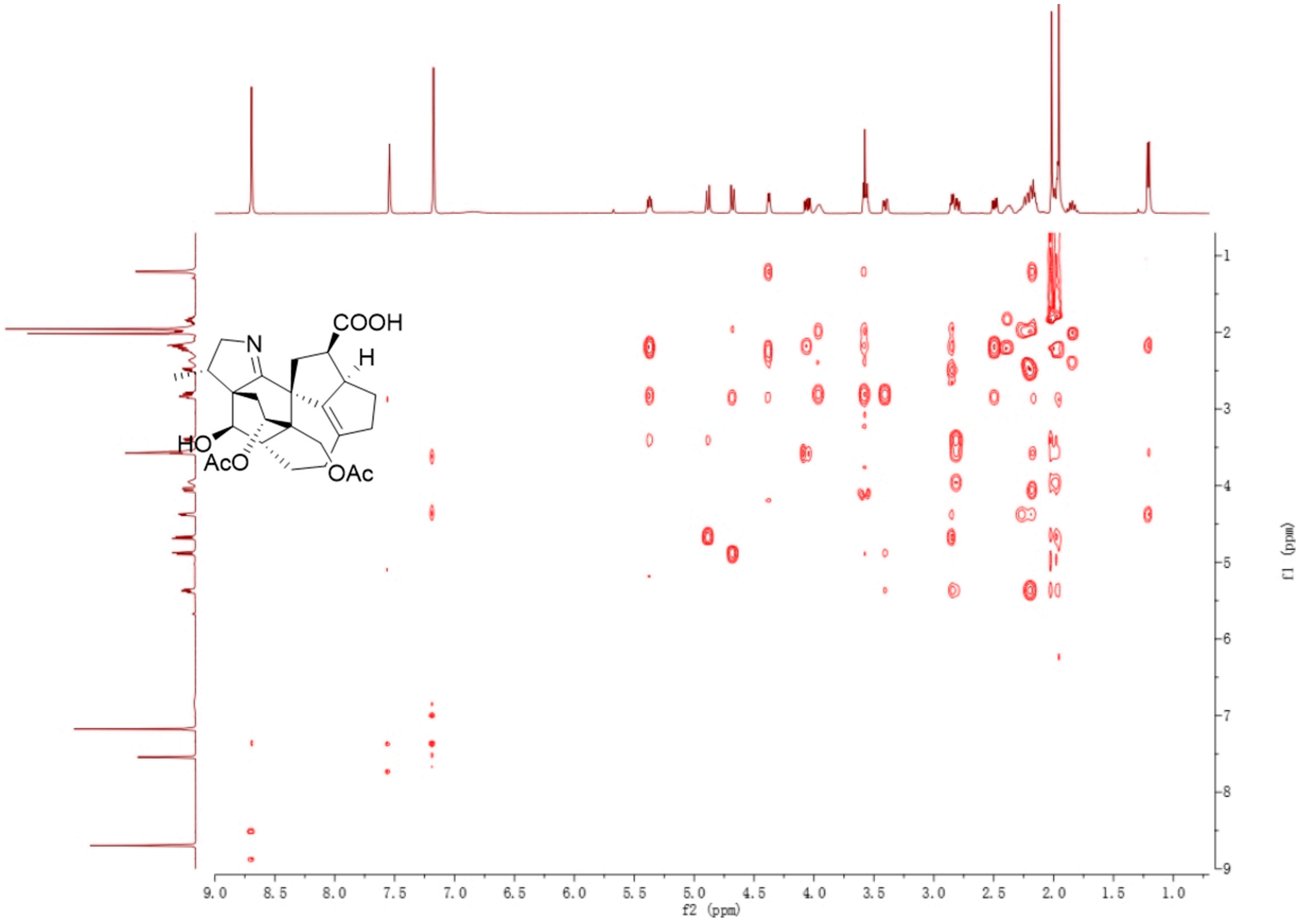

Figure S45 HR-ESI-MS spectrum of compound **5**

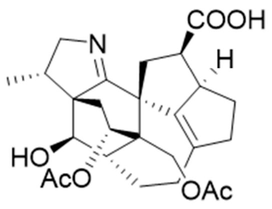

User Spectra

Fragmentor Voltage	Collision Energy	Ionization Mode
135	0	ESI

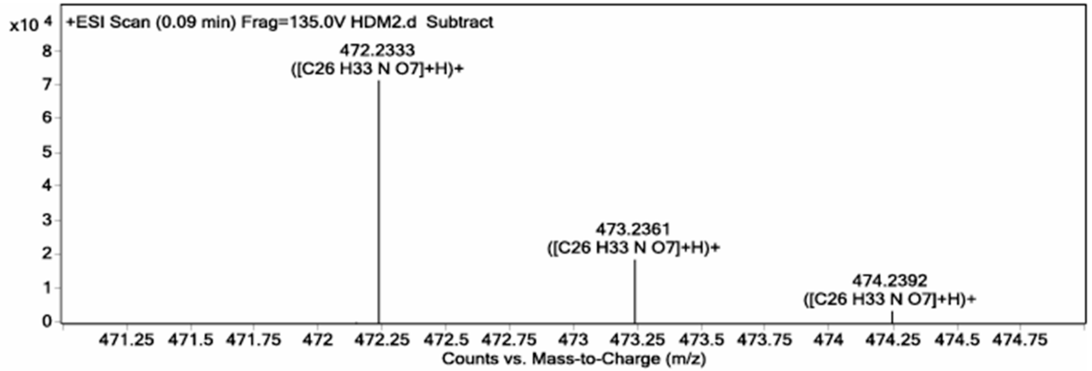

Peak List

m/z	z	Abund	Formula	Ion
85.0811	1	8359.46		
86.0846	1	957.51		
89.5072	1	1214.52		
122.0534	1	918.2		
414.2258	1	1850.32		
453.1672	1	942.76		
472.2333	1	71606.95	C26 H33 N O7	(M+H)+
473.2361	1	18662.82	C26 H33 N O7	(M+H)+
474.2392	1	3485.05	C26 H33 N O7	(M+H)+
494.2149	1	1621.7		

Formula Calculator Element Limits

Element	Min	Max
C	3	60
H	0	200
O	0	30
N	0	5

Formula Calculator Results

Formula	CalculatedMass	CalculatedMz	Mz	Diff. (mDa)	Diff. (ppm)	DBE
C26 H33 N O7	471.2257	472.2330	472.2333	-0.30	-0.64	11.0000

Figure S46 IR (KBr disk) spectrum of compound **5**

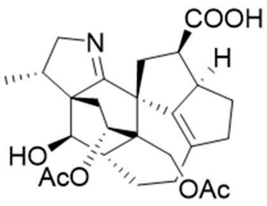

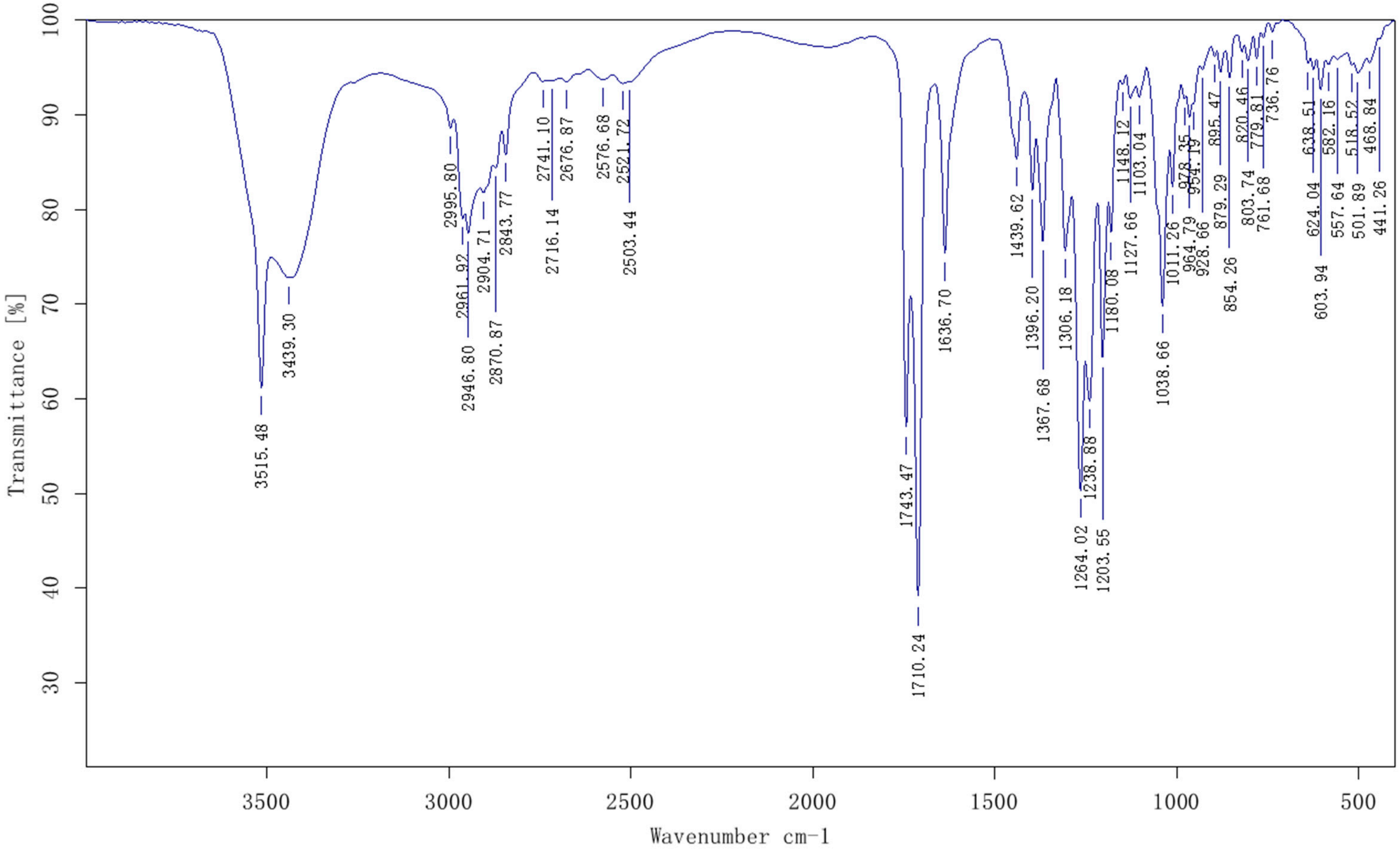

Sample Name: HDM2
Sample Form: KBr
Path of File: E:\data
Date of Measurement: 2024/7/23

Resolution: 4
Aperture Setting: 6 mm
Number of Background Scans: 16
Number of Sample Scans: 16

Beamsplitter Setting: KBr
Source Setting: MIR
Instrument Type: BRUKER VERTEX 70
Soft Version: OPUS8.1

Figure S47 UV spectrum of compound **5**

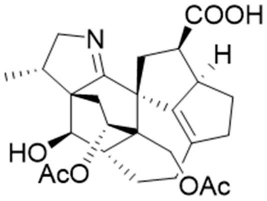

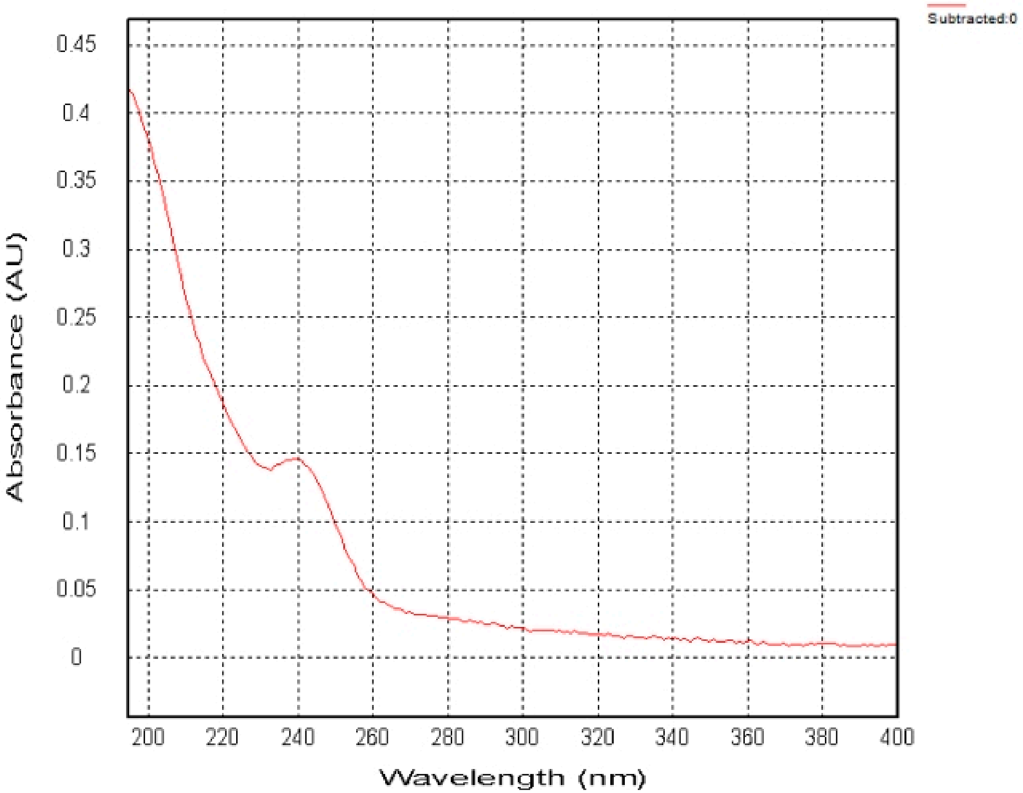

Figure S48 ECD spectrum of compound **5**

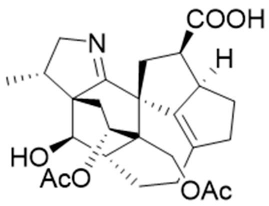

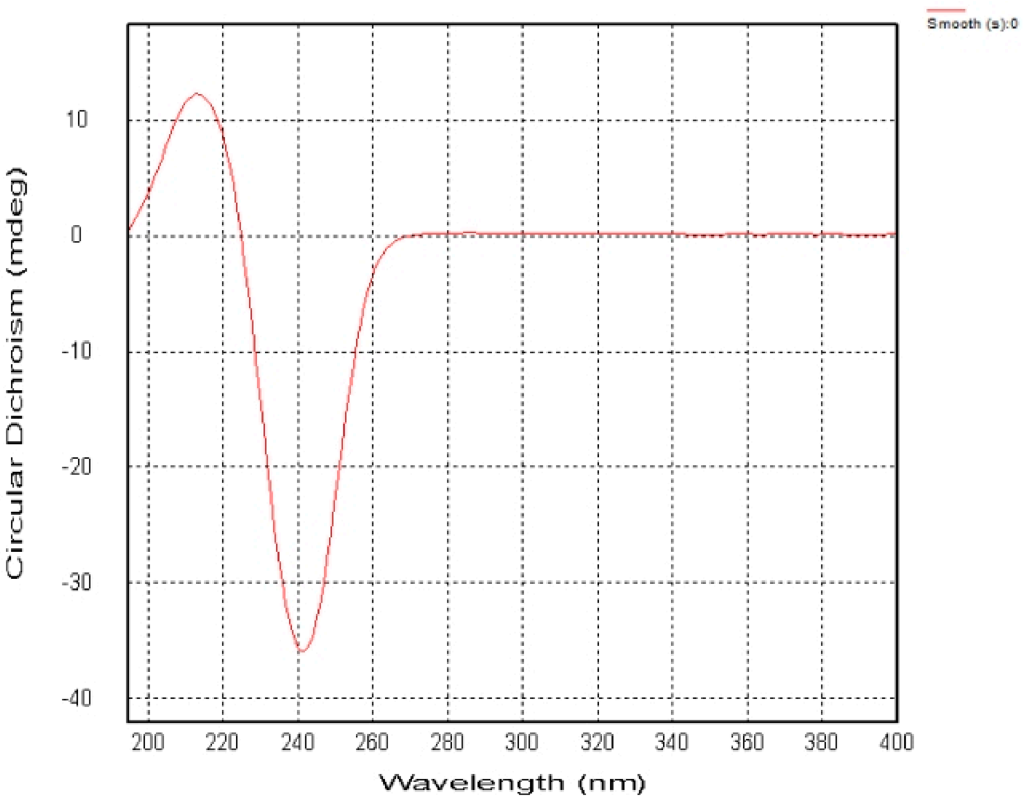

Figure S49 View of the hydrogen-bonded motif of daphmacropomine A (**1**)

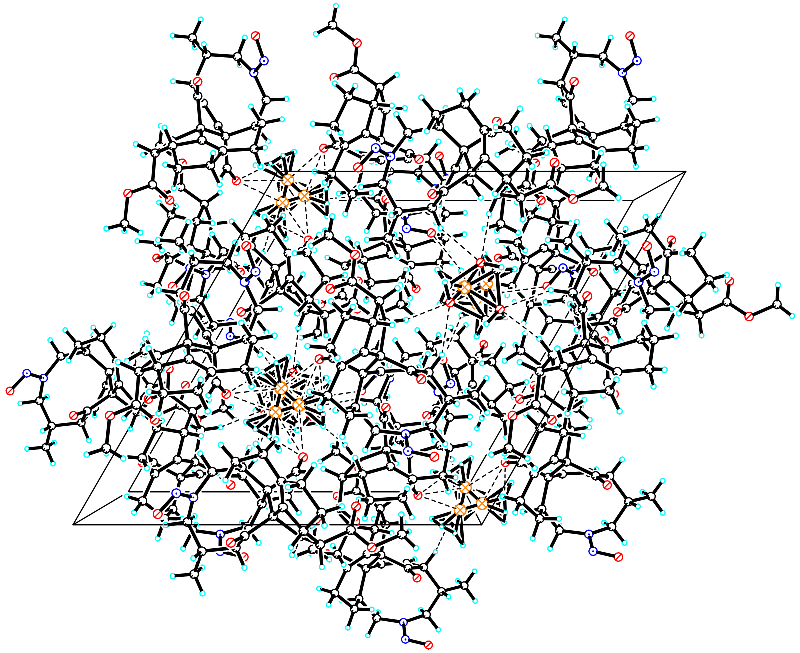

View of the hydrogen-bonded motif of daphmacropomine A (**1**).
Hydrogen-bonds are shown as dashed lines.

Figure S50 View of the hydrogen-bonded motif of daphmacropomine B (**2**)

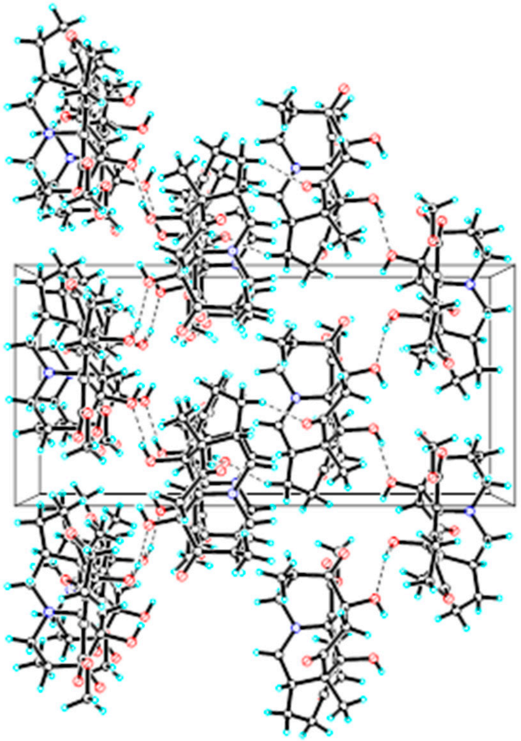

View of the hydrogen-bonded motif of daphmacropomine B (**2**).
Hydrogen-bonds are shown as dashed lines.
