# Supplementary figures and images for: Daphmacropomines A–E: Five Daphniphyllum Alkaloids from Daphniphyllum macropodum Miq
Source: Molecules. 2026 Jun 3;31(11):1943. doi: 10.3390/molecules31111943 (PMC13258748; doi:10.3390/molecules31111943)

## Slide 1
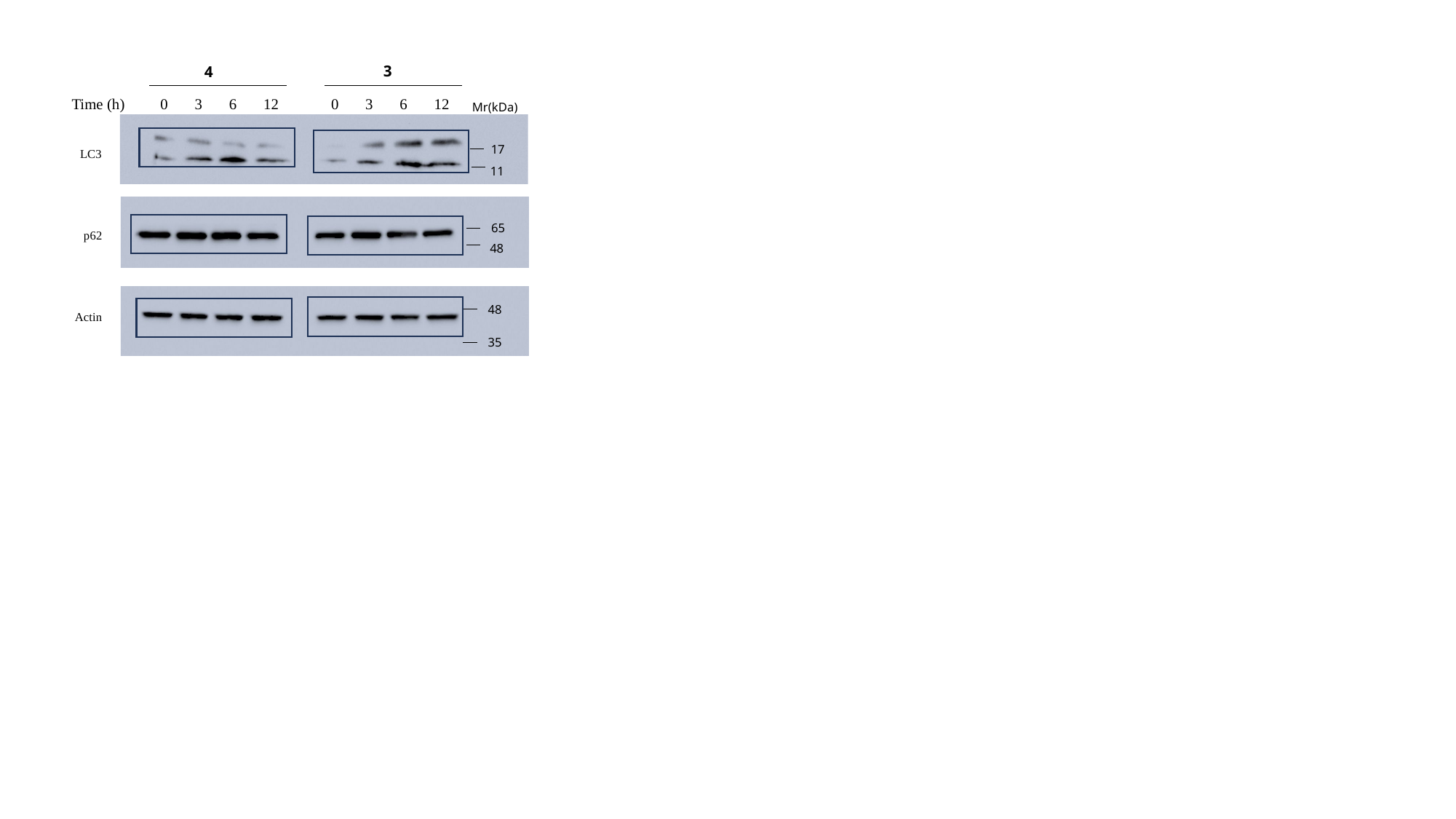

3
4
0 3 6 12
Time (h)
0 3 6 12
Mr(kDa)
17
11
LC3
65
48
p62
48
35
Actin

Supplement: Supplementary file 1 [file molecules-31-01943-s001.zip › original Western Blot Images.pptx]
